# The global, regional, and national burden of inflammatory bowel disease in 195 countries and territories, 1990–2017: a systematic analysis for the Global Burden of Disease Study 2017

**DOI:** 10.1016/S2468-1253(19)30333-4

**Published:** 2019-10-21

**Authors:** GBD 2017 Inflammatory Bowel Disease Collaborators, Sudabeh Alatab, Sadaf G Sepanlou, Kevin Ikuta, Homayoon Vahedi, Catherine Bisignano, Saeid Safiri, Anahita Sadeghi, Molly R Nixon, Amir Abdoli, Hassan Abolhassani, Vahid Alipour, Majid A H Almadi, Amir Almasi-Hashiani, Amir Anushiravani, Jalal Arabloo, Suleman Atique, Ashish Awasthi, Alaa Badawi, Atif A A Baig, Neeraj Bhala, Ali Bijani, Antonio Biondi, Antonio M Borzì, Kristin E Burke, Félix Carvalho, Ahmad Daryani, Manisha Dubey, Aziz Eftekhari, Eduarda Fernandes, João C Fernandes, Florian Fischer, Arvin Haj-Mirzaian, Arya Haj-Mirzaian, Amir Hasanzadeh, Maryam Hashemian, Simon I Hay, Chi L Hoang, Mowafa Househ, Olayinka S Ilesanmi, Nader Jafari Balalami, Spencer L James, Andre P Kengne, Masoud M Malekzadeh, Shahin Merat, Tuomo J Meretoja, Tomislav Mestrovic, Erkin M Mirrakhimov, Hamid Mirzaei, Karzan A Mohammad, Ali H Mokdad, Lorenzo Monasta, Ionut Negoi, Trang H Nguyen, Cuong T Nguyen, Akram Pourshams, Hossein Poustchi, Mohammad Rabiee, Navid Rabiee, Kiana Ramezanzadeh, David L Rawaf, Salman Rawaf, Nima Rezaei, Stephen R Robinson, Luca Ronfani, Sonia Saxena, Masood Sepehrimanesh, Masood A Shaikh, Zeinab Sharafi, Mehdi Sharif, Soraya Siabani, Ali Reza Sima, Jasvinder A Singh, Amin Soheili, Rasoul Sotoudehmanesh, Hafiz Ansar Rasul Suleria, Berhe E Tesfay, Bach Tran, Derrick Tsoi, Marco Vacante, Adam B Wondmieneh, Afshin Zarghi, Zhi-Jiang Zhang, Mae Dirac, Reza Malekzadeh, Mohsen Naghavi

## Abstract

**Background:**

The burden of inflammatory bowel disease (IBD) is rising globally, with substantial variation in levels and trends of disease in different countries and regions. Understanding these geographical differences is crucial for formulating effective strategies for preventing and treating IBD. We report the prevalence, mortality, and overall burden of IBD in 195 countries and territories between 1990 and 2017, based on data from the Global Burden of Diseases, Injuries, and Risk Factors Study (GBD) 2017.

**Methods:**

We modelled mortality due to IBD using a standard Cause of Death Ensemble model including data mainly from vital registrations. To estimate the non-fatal burden, we used data presented in primary studies, hospital discharges, and claims data, and used DisMod-MR 2.1, a Bayesian meta-regression tool, to ensure consistency between measures. Mortality, prevalence, years of life lost (YLLs) due to premature death, years lived with disability (YLDs), and disability-adjusted life-years (DALYs) were estimated. All of the estimates were reported as numbers and rates per 100 000 population, with 95% uncertainty intervals (UI).

**Findings:**

In 2017, there were 6·8 million (95% UI 6·4–7·3) cases of IBD globally. The age-standardised prevalence rate increased from 79·5 (75·9–83·5) per 100 000 population in 1990 to 84·3 (79·2–89·9) per 100 000 population in 2017. The age-standardised death rate decreased from 0·61 (0·55–0·69) per 100 000 population in 1990 to 0·51 (0·42–0·54) per 100 000 population in 2017. At the GBD regional level, the highest age-standardised prevalence rate in 2017 occurred in high-income North America (422·0 [398·7–446·1] per 100 000) and the lowest age-standardised prevalence rates were observed in the Caribbean (6·7 [6·3–7·2] per 100 000 population). High Socio-demographic Index (SDI) locations had the highest age-standardised prevalence rate, while low SDI regions had the lowest age-standardised prevalence rate. At the national level, the USA had the highest age-standardised prevalence rate (464·5 [438·6–490·9] per 100 000 population), followed by the UK (449·6 [420·6–481·6] per 100 000). Vanuatu had the highest age-standardised death rate in 2017 (1·8 [0·8–3·2] per 100 000 population) and Singapore had the lowest (0·08 [0·06–0·14] per 100 000 population). The total YLDs attributed to IBD almost doubled over the study period, from 0·56 million (0·39–0·77) in 1990 to 1·02 million (0·71–1·38) in 2017. The age-standardised rate of DALYs decreased from 26·5 (21·0–33·0) per 100 000 population in 1990 to 23·2 (19·1–27·8) per 100 000 population in 2017.

**Interpretation:**

The prevalence of IBD increased substantially in many regions from 1990 to 2017, which might pose a substantial social and economic burden on governments and health systems in the coming years. Our findings can be useful for policy makers developing strategies to tackle IBD, including the education of specialised personnel to address the burden of this complex disease.

**Funding:**

Bill & Melinda Gates Foundation.

## Introduction

Inflammatory bowel disease (IBD) is characterised by non-infectious chronic inflammation of the gastrointestinal tract, and primarily includes Crohn's disease (which can affect any segment of the gastrointestinal tract from the mouth to the anus), ulcerative colitis (which is limited to the colonic mucosa), and indeterminate colitis.^1^ Crohn's disease and ulcerative colitis both commonly present with abdominal pain and diarrhoea. Rectal bleeding occurs more frequently with ulcerative colitis than with Crohn's disease, but patients with Crohn's disease often experience weight loss and perianal disease.^2^ The peak of IBD's occurrence, followed by a chronic relapsing pattern, usually happens in the second to fourth decade of life, the most productive time of adulthood.^1^ IBD can adversely affect all aspects of an individual's life.^1^ Although the cause of IBD is still not completely understood, IBD is suggested to be a result of uncontrolled immune response to a trigger in genetically prone individuals.^1^ The role of environmental factors either as the triggers or causes of the uncontrolled immune response continues to be debated.^1^

**Research in context**

**Evidence before this study**

Inflammatory bowel disease (IBD) imposes health and economic burdens on communities worldwide, and substantially reduces patients' quality of life. It is estimated that more than 3 million people in the USA and Europe have IBD, and its prevalence is estimated to exceed 0·3% in North America, Oceania, and many countries in Europe. Evidence from systematic reviews points to a changing epidemiology of IBD, with stable or decreasing incidence in North America and Europe, and increasing incidence in newly industrialised countries. Most of these studies have originated in high-income countries, and countries with lower socioeconomic status have produced few, if any, population-based studies that report prevalence, incidence, or IBD-related deaths. These studies also do not include estimation over time and usually do not include estimates for years of life lost (YLLs), years lived with disability (YLDs), or disability-adjusted life-years (DALYs) across either countries or geographically related regions.

**Added value of this study**

The Global Burden of Diseases, Injuries, and Risk Factors Study (GBD) 2017 used an integrated modelling approach to estimate not only the epidemiological parameters for regions with available data, but also for countries and territories, as well as regions in which sufficient data are not available. GBD 2017 provided estimates of the burden of IBD for seven super-regions, 21 regions, and 195 countries and territories from 1990 to 2017. There has been no previous dedicated and detailed publication of GBD methods and estimates for IBD. We report the burden of IBD, including prevalence, mortality, YLDs, and DALYs, by age, sex, and Socio-demographic Index (SDI) from 1990 to 2017, using all available data and based on standardised GBD methods at the global, regional, and national levels. In addition to exploring country-level variation in the burden of IBD by development level, we assessed the temporal patterns and changes in geographical patterns of the burden of IBD. We believe this analysis is the most comprehensive picture of IBD burden to date.

**Implication of all the available evidence**

Between 1990 and 2017, the global number of prevalent cases of IBD increased. After many years of sharp increases in IBD incidence in North America and western European countries, and because of improved survival, a pattern of increased prevalence emerged in these regions. An alarming trend for health systems is the observed rise in prevalence in newly industrialised countries. The full effect of this rise might not yet have been fully appreciated, because IBD symptoms persist throughout life, producing prominent disability and morbidity. The information provided in this study could be crucial for researchers, clinicians, and health policy makers to prepare their clinical infrastructure and educate specialised personnel to be able to confront the burden of this complex, and socially and economically costly disease. Moreover, the rising pattern of IBD provides a unique opportunity for researchers to focus on identifying the environmental risk factors contributing to IBD.

Traditionally, IBD has been regarded as a disease of high-income nations, but a shift in the epidemiological pattern has been reported, indicating stabilising incidence in high-income countries with a high burden and prevalence, and a rapid rise in newly industrialised countries in South America, eastern Europe, Asia, and Africa.^3^ These rapid changes call for global estimates to provide insight into the burden and trends. Moreover, defining the varying incidence, prevalence, and prognosis of IBD in different geographical regions might provide researchers with clues to the cause of the disease.^4^

We report the IBD burden in 195 countries and territories from 1990 to 2017, based on the most recent Global Burden of Diseases, Injuries, and Risk Factors Study (GBD) 2017 estimates in terms of prevalence, mortality, years lived with disability (YLDs), years of life lost (YLLs), and disability-adjusted life-years (DALYs).

## Methods

### Overview

This study is part of GBD 2017, which, to the best of our knowledge, is the most comprehensive and systematic effort to estimate the burden of diseases, injuries, and risk factors at global, regional, and national levels to date. GBD 2017 estimated 359 diseases and injuries; 282 causes of death; and 84 behavioural, environmental and occupational, and metabolic risk factors. The detailed methods are published elsewhere.^3^, ^5^, ^6^

Crohn's disease and ulcerative colitis (ie, the two types of IBD we investigated) are diagnosed by endoscopy, imaging studies, or biopsy in a patient with relevant clinical signs and symptoms. In some cases of IBD, neither Crohn's disease nor ulcerative colitis can be definitely diagnosed, and a diagnosis of indeterminate colitis is applied indefinitely or until definitive features of Crohn's disease or ulcerative colitis are identifiable.^7^ International Classification of Disease version 10 (ICD-10) codes were K50 for Crohn's disease, K51 for ulcerative colitis, and K52 for indeterminate colitis.

### Mortality estimates

To model IBD mortality, we used the causes of death database, which includes data from vital registration and verbal autopsy data. The data processing for the causes of death data has been described previously.^5^ We marked data as outliers if garbage code redistribution and noise reduction in combination with small sample sizes resulted in unreasonable cause fractions, as well as data that violated well-established time or age trends.

We modelled deaths due to IBD with a standard Cause of Death Ensemble model (CODEm), using the causes of death database and location-level covariates as inputs. We hybridised separate global and data-rich models for each sex to obtain unadjusted results. We then finalised and adjusted estimates to be consistent with all-cause mortality levels for each age–sex–year location using the cause of death correct procedure (CODCorrect) to reach final YLLs due to IBD.^5^ The method for propagating uncertainty was similar to that used in previous GBD papers.^5^ The distribution of every step in the computation process was stored in 1000 draws that were used for every other step in the process. Final estimates were computed using the mean estimate across 1000 draws, and the 95% uncertainty intervals (UIs) were specified on the basis of the 25th and 975th ranked values across all 1000 draws.

The percentage change between any 2 years of estimates in GBD was calculated at the draw level. Every one of 1000 draws for 2017 was compared with the corresponding draw for 1990 to generate 1000 percent change draws. The mean of the draws and the 25th and 975th ordered draws were then used as the mean, lower UI limit, and upper UI limit for reporting the percentage change.

### Non-fatal estimates

To estimate the non-fatal burden of IBD, we used two separate databases, one for Crohn's disease and another for ulcerative colitis. Both included data from literature, hospital discharges, and claims data (the latter available only from the USA in 2000, 2010, and 2012; further information on IBD data is provided in the appendix, p 21). Claims data link multiple inpatient and outpatient claims to a single individual; prevalent cases were extracted if an individual had at least one inpatient or outpatient encounter with an appropriate ICD code as any diagnosis. Data from hospital discharges were adjusted using correction factors from claims, converting encounters to estimates of cases, correcting for some facilities providing only primary diagnostic codes, and estimating outpatient cases from inpatient cases. Literature data came from a systematic review done for GBD 2016.^8^ In brief, this systematic review of literature was done to capture studies of the prevalence and incidence of IBD. Studies were excluded if they were not representative of the national population, or if they had insufficient or inappropriate sampling methods. Reviews were excluded from the search results.

The prevalence and incidence data described earlier were entered into separate models for ulcerative colitis and Crohn's disease in DisMod-MR 2.1, a Bayesian meta-regression tool, as the main method of estimation, ensuring consistency between rates of prevalence, incidence, remission, and cause of death for each non-fatal condition. Outputs from DisMod were then adjusted to account for IBD due to indeterminate colitis.

For both ulcerative colitis and Crohn's disease, the DisMod models used prevalence and incidence data, as described above. Reference data were claims from the USA from 2012, and study-level covariates were used to mark data from literature, hospital discharges, US claims in 2000 and 2010, and the Medical Expenditure Panel Survey. The study-level covariate for hospital discharges for Crohn's disease was found to have no significant effect and was later dropped during data analysis. For ulcerative colitis, a prior value on remission was set to zero for all age groups, and an incidence prior value was set to zero only for ages zero to 1 year. For Crohn's disease, a prior value on remission was set to zero for all age groups, and on incidence a prior value was set to zero only for ages zero to 2 years. Location-level covariates were log-transformed lag-distributed income and Healthcare Access and Quality Index (both on excess mortality) and log-transformed age-standardised death rate due to both types of IBD (on prevalence).

The proportion of IBD cases categorised as indeterminate colitis was determined to be 0·624 (95% UI 0·0549–0·0699) via meta-analysis. To account for all IBD cases, an adjustment of 1·0624 (1·0549–1·0699) was applied to the outputs of our ulcerative colitis and Crohn's disease models. This approach assumes that all cases initially diagnosed as indeterminant ultimately declare themselves to be one of these two defined diseases.

Estimates of prevalence were combined with disability weights to estimate YLDs. The basis of the GBD disability weight survey assessments are lay descriptions of sequelae highlighting major functional consequences and symptoms. For GBD 2017, we used the Medical Expenditure Panel Survey to find the proportion of ulcerative colitis and Crohn's disease that was asymptomatic versus symptomatic during a given 4-week period. The lay description for either of these diseases in case they were symptomatic was defined as a person who has cramping abdominal pain, has diarrhoea several times a day, and feels very tired for 2 months every year, and when the person does not have symptoms, there is anxiety about them returning. The disability weight for symptomatic Crohn's disease and ulcerative colitis was 0·231 (95% UI 0·156–0·320).

We estimated the burden of IBD in terms of mortality, prevalence, YLLs, YLDs, and DALYs, which are the sum of YLLs and YLDs, for both sexes, 20 age groups, and 195 countries and territories from 1990 to 2017. The rates were age-standardised according to the GBD world population and are reported per 100 000 population.^9^ 95% UIs were reported for all estimates. This study is compliant with the Guidelines for Accurate and Transparent Health Estimates Reporting.

### Role of the funding source

The funder of the study had no role in study design; the collection, analysis, or interpretation of the data; or the writing of the report. The corresponding author had full access to all of the data and the final responsibility to submit for publication.

## Results

**Figure 1:**
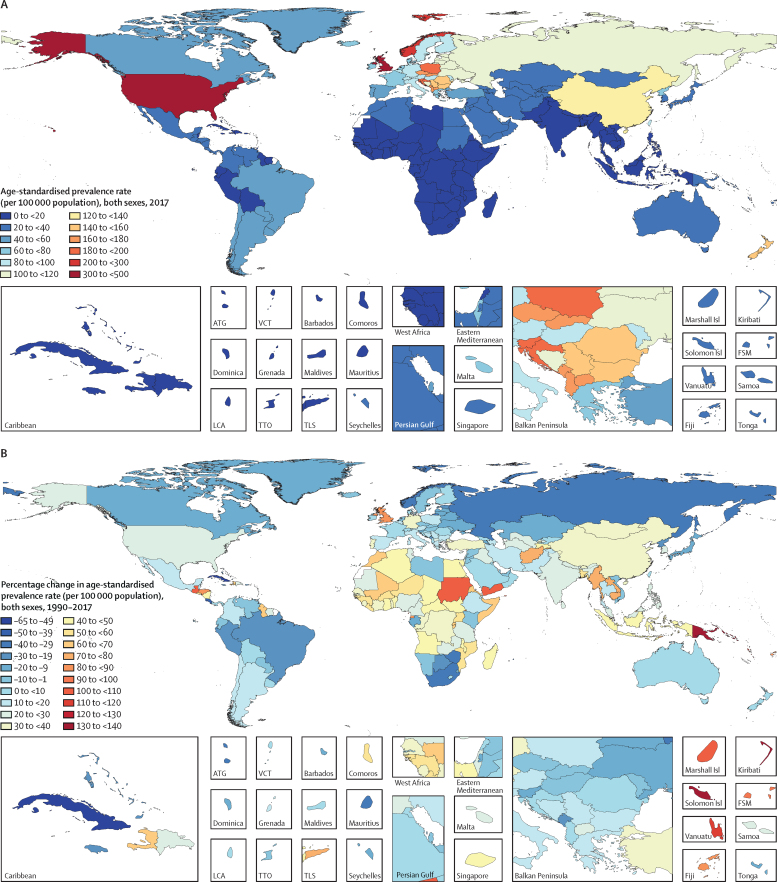
(A) Age-standardised prevalence rate (per 100 000 population) of IBD, both sexes, for 195 countries and territories, 2017. (B) Percentage change in age-standardised prevalence rate (per 100 000 population) of IBD, both sexes, for 195 countries and territories, 1990–2017 IBD=inflammatory bowel disease. ATG=Antigua and Barbuda. VCT=Saint Vincent and the Grenadines. LCA=Saint Lucia. TTO=Trinidad and Tobago. TLS=Timor-Leste. FSM=Federated States of Micronesia.

**Figure 2:**
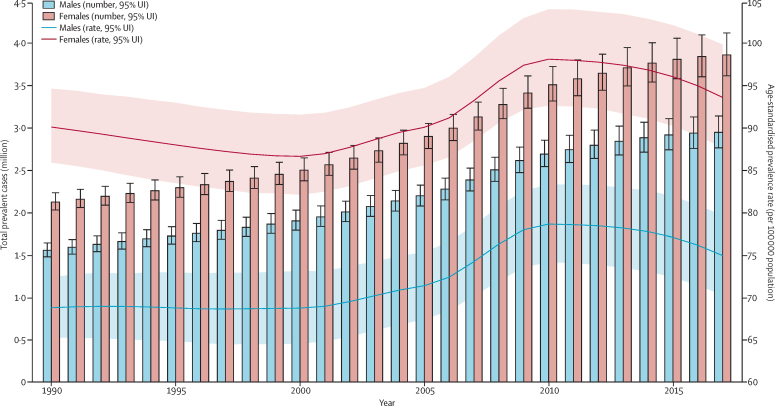
Trends from 1990 to 2017 in number and age-standardised prevalence rates of IBD at the global level Error bars indicate the 95% uncertainty interval (UI) for prevalent cases. Shading indicates the 95% UI for the age-standardised prevelance rate. IBD=inflammatory bowel disease.

**Figure 3:**
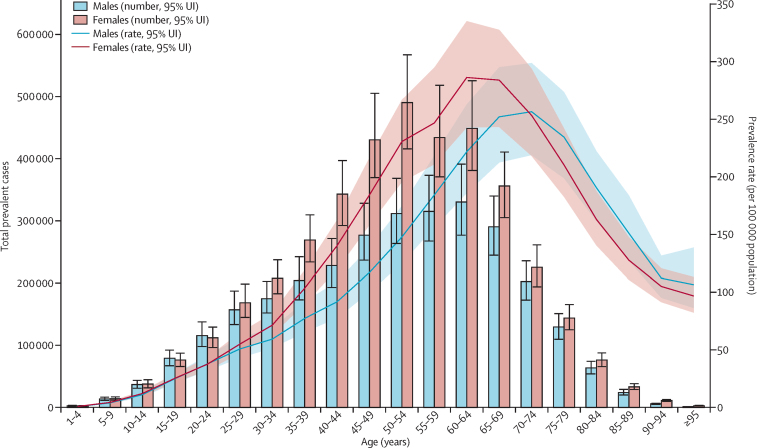
Age patterns by sex in 2017 of the total number of prevalent cases and age-specific prevalence rate of IBD at the global level IBD=inflammatory bowel disease.

Between 1990 and 2017, the number of individuals with IBD increased from 3·7 million (95% UI 3·5–3·9) to more than 6·8 million (6·4–7·3), an increase of 85·1% (79·5–89·9) in global prevalent cases of IBD (appendix p 22). However, the global age-standardised prevalence rate of IBD showed only a 6·1% (3·3–8·6) increase, from 79·5 (75·9–83·5) per 100 000 population in 1990 to 84·3 (79·2–89·9) per 100 000 population in 2017. The global map of age-standardised prevalence rate of IBD and percentage change in age-standardised prevalence at the country level are presented in figure 1. Both the number of prevalent cases and age-standardised prevalence rate were significantly higher in females than males in all years from 1990 to 2017 (figure 2). Overall, nearly 3·9 million (3·6–4·1) prevalent cases (57%) occurred among females in 2017, and nearly 3·0 million (2·8–3·2) cases (43%) occurred in males. The age-standardised prevalence rate was 75·0 (70·3–79·7) per 100 000 population in males and 93·8 (87·8–100·0) per 100 000 population in females in 2017. The highest peak of IBD age-specific prevalence rate occurred at age 60–64 years in females, whereas the peak was at age 70–74 years in males (figure 3).

**Figure 4:**
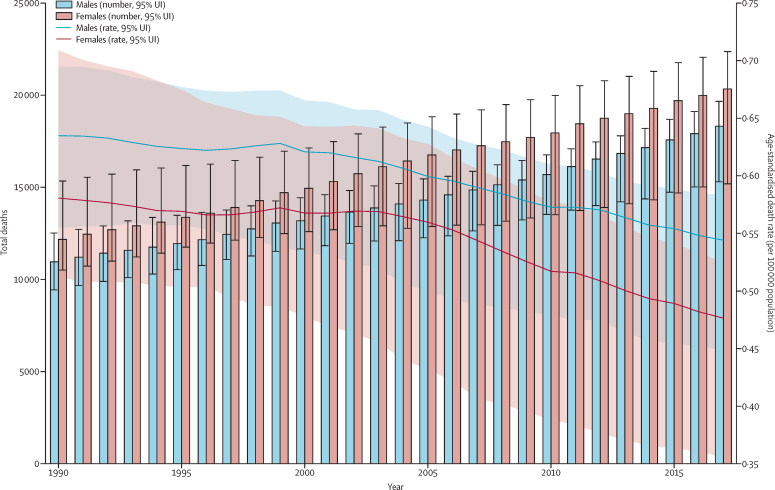
Trends from 1990 to 2017 in number and age-standardised death rates of IBD at the global level Error bars indicate the 95% uncertainty interval (UI) for number of deaths. Shading indicates the 95% UI for the age-standardised death rate. IBD=inflammatory bowel disease.

The total number of IBD-related deaths increased by 67·0% (95% UI 23·6–96·1) from 1990 to 2017, from 23 000 (20 000–27 000) to 38 000 (32 000–41 000; figure 4; appendix p 30). Despite this rise, the global age-standardised death rate decreased from 0·61 (0·55–0·69) per 100 000 population in 1990 to 0·51 (0·42–0·54) per 100 000 population in 2017, a rate that corresponded with a 16·4% (36·0–4·7) decrease in age-standardised death rate over the study period (appendix p 30). The total number of deaths caused by IBD constituted 0·07% (0·06–0·07) of total all-cause deaths in 2017 (estimates available through the GBD results tool). In 2017, the number of deaths from IBD was highest in females aged 85–89 years and males aged 80–84 years, whereas the age-specific rate of death was highest in the group aged 95 years and older for both sexes (appendix p 3).

The total YLDs attributed to IBD almost doubled over the study period, from 0·56 million (95% UI 0·39–0·77) in 1990 to 1·02 million (0·71–1·38) in 2017 (estimates available through the GBD results tool). However, the age-standardised YLDs rate did not have the same sharp increase (12·0 [8·4–16·5] per 100 000 population in 1990 to 12·6 [8·7–17·0] per 100 000 population in 2017). The number of YLDs in 2017 peaked in the 50–54 years age group (0·12 million [0·08–0·16]; appendix p 4) and then declined in the older age groups. Among the diseases in the GBD digestive disease category, IBD rose in YLD rank (both number of YLDs and age-standardised rate) from fifth in 1990 to fourth in 2017, after upper digestive diseases, hernia, and cirrhosis (estimates available through the online data visualisation tool).

**Figure 5:**
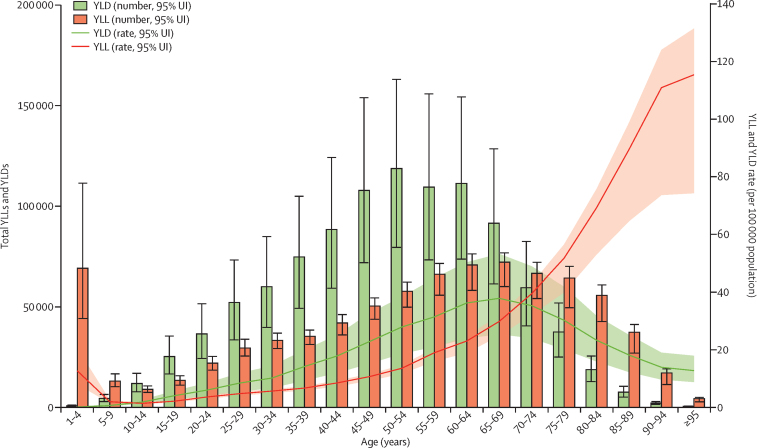
Global counts and age-specific rates of YLLs and YLDs due to IBD across age groups, 2017 Error bars indicate the 95% uncertainty interval (UI) for YLLs and YLDs. Shading indicates the 95% UI for the rates. YLLs=years of life lost. YLDs=years of life lived with disability. IBD=inflammatory bowel disease.

The total YLLs attributed to IBD in 1990 was 0·68 million (95% UI 0·54–0·96), which increased to 0·83 million (0·71–0·90) in 2017. The age-standardised rates of YLLs declined over time for both sexes, from 14·5 YLLs (11·8–19·0) per 100 000 population in 1990 to 10·7 YLLs (9·1–11·7) per 100 000 population in 2017, for both sexes combined. In 2017, the highest number of YLLs occurred in the group aged 65–69 years (72 000 [60 000–77 000]), while the highest number of YLDs occurred in the 50–54 year age group (119 000 [80 000–163 000]; figure 5). The 1–4 year age group had the third highest number of YLLs, because each death at a young age results in more YLLs than at older ages, but had the second lowest number of YLDs (figure 5).

The age-standardised rate of DALYs decreased from 26·5 (95% UI 21·0–33·0) per 100 000 population in 1990 to 23·2 (19·1–27·8) per 100 000 population in 2017 (appendix p 38). The total DALYs caused by IBD increased between 1990 (1·25 million [0·97–1·61]) and 2017 (1·85 million [1·51–2·23]; appendix p 38). Of the total DALYs caused by IBD in 2017, 45% were due to YLLs and 55% were due to YLDs.

In 2017, the highest age-standardised prevalence rate among all seven super-regions was observed in the high-income super-region (206·1 [95% UI 195·3–216·8] per 100 000 population; appendix p 8). Moreover, this region had the highest increase in the age-standardised prevalence rate from 1990 to 2017 (31·3% [26·4–36·6]; percentage changes for other super-regions available from the GBD results tool).

**Figure 6:**
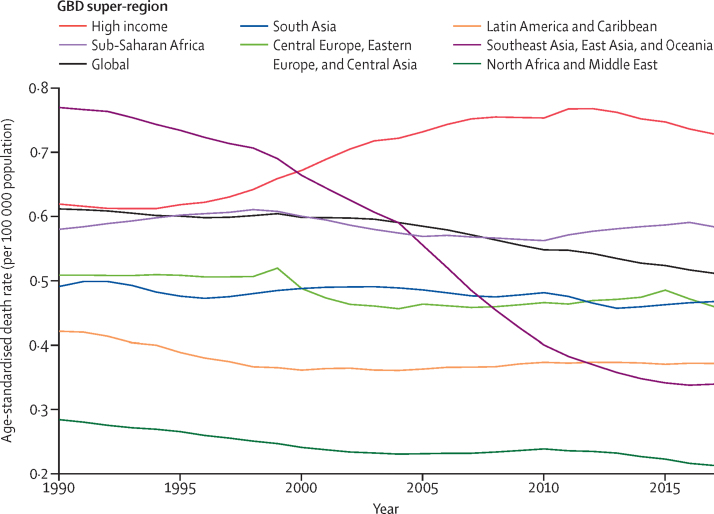
Trends from 1990 to 2017 in age-standardised death rate of IBD in seven GBD super-regions IBD=inflammatory bowel disease. GBD=Global Burden of Disease, Injuries, and Risk Factors Study.

The high-income super-region also had the largest increase in age-standardised death rate from 1990 to 2017 (17·6% [95% UI −35·7 to 34·7]; figure 6). Sub-Saharan Africa was the only other super-region to experience an increase in age-standardised death rate over the study period, a 0·7% (−32·5 to 43·6) increase (figure 6). The total number of deaths caused by IBD in the high-income super-region increased from 7440 (95% UI 6910–9550) in 1990 to 16 900 (10 300–18 800) in 2017 (super-region data available from the GBD results tool). North Africa and the Middle East remained the super-region with the lowest number of deaths, with 872 (781–999) deaths in 2017. The number of deaths was 372 (317–432) in females and 500 (436–605) in males. The 2017 age-standardised death rate in this super-region was also the lowest among all GBD super-regions (0·21 [0·19–0·25] per 100 000 population). A sharp decrease in the age-standardised death rate also occurred in southeast Asia, east Asia, and Oceania from 1990 to 2017 (figure 6).

**Figure 7:**
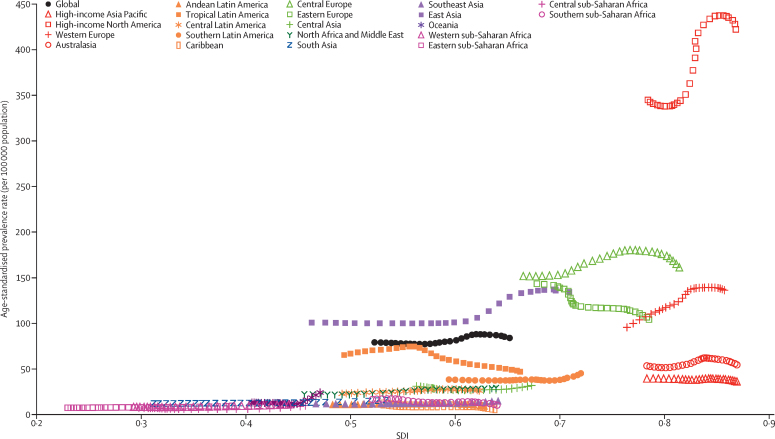
Age-standardised prevalence rate of IBD globally and for 21 GBD regions by SDI, 1990–2017 For each region, points from left to right depict estimates from each year from 1990 to 2017. IBD=inflammatory bowel disease. GBD=Global Burden of Disease, Injuries, and Risk Factors Study.

High-income North America was the region with the highest age-standardised prevalence rate for both sexes between 1990 and 2017 (344·8 [95% UI 331·7–359·3] per 100 000 population in 1990 and 422·0 [398·7–466·1] per 100 000 population in 2017 for both sexes combined; figure 7; appendix pp 11, 22). The lowest age-standardised prevalence rate in 2017 was observed in the Caribbean (6·7 [6·3–7·2] per 100 000 population; appendix pp 22–29), followed by Andean Latin America and the four sub-Saharan Africa regions.

**Figure 8:**
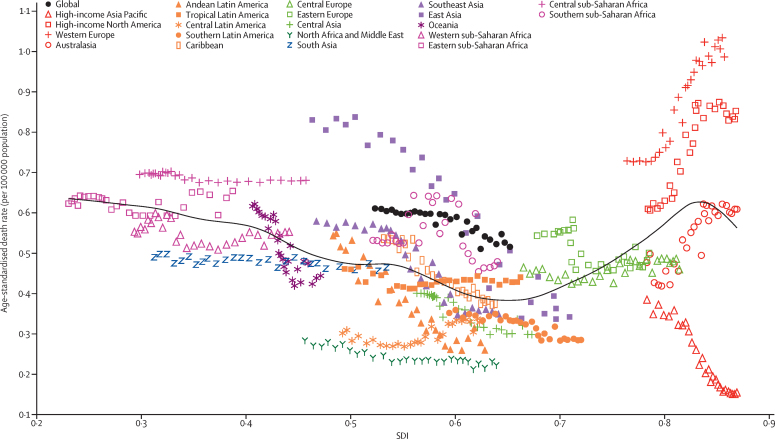
Age-standardised death rate of IBD globally and for 21 GBD regions by SDI, 1990–2017 The expected age-standardised death rate in 2017 based solely on SDI is represented by the black line. For each region, points from left to right depict estimates from each year from 1990 to 2017. IBD=inflammatory bowel disease. SDI=Socio-demographic Index.

The western Europe region had the highest age-standardised death rate in 2017 (0·97 [95% UI 0·54–1·11] per 100 000 population), followed by high-income North America (0·83 [0·55–0·91] per 100 000 population; appendix pp 13, 30–37), driven primarily in high-income North America by high number of deaths in the USA (appendix p 30). Both western Europe (33·6% [–37·5 to 59·0]) and high-income North America (36·9% [–22·3 to 57·1]) experienced an increase in age-standardised death rate over the study period (figure 8). The age-standardised death rate decreased sharply in east Asia (59·6% [66·3–27·3] decrease from 1990 to 2017; appendix pp 14, 33). High-income Asia Pacific (0·16 [0·13–0·24] per 100 000 population), north Africa and the Middle East (0·21 [0·19–0·25] per 100 000 population), and Andean Latin America (0·26 [0·22–0·29] per 100 000 population) had the lowest age-standardised death rate among regions in 2017 (figure 8; appendix p 13).

**Figure 9:**
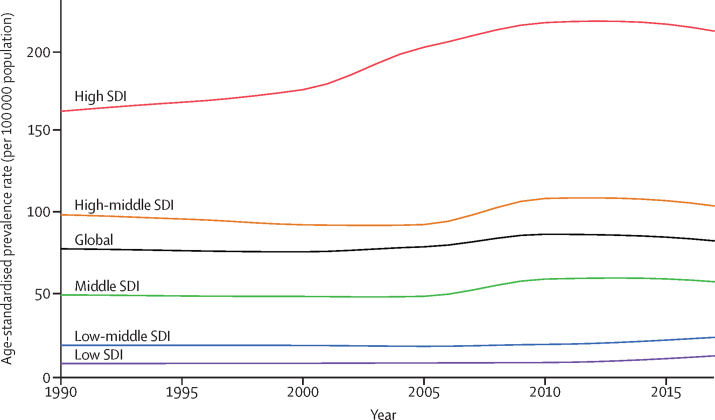
Trends from 1990 to 2017 in age-standardised prevalence rates of IBD by SDI quintile IBD=inflammatory bowel disease. SDI=Socio-demographic Index.

Higher SDI was associated with higher age-standardised prevalence rates of IBD, with values that were higher than the global rate in the two highest SDI quintiles, and lower than the global rate in the three lowest SDI quintiles (figure 9). High SDI and low SDI quintiles had the highest and lowest age-standardised prevalence rates (213·0 [95% UI 202·3–223·8] per 100 000 population and 13·8 [12·6–15·2] per 100 000 population), respectively, in 2017 (figure 8). By contrast, while the high SDI quintile (0·71 [0·44–0·78] per 100 000 population) also had the highest age-standardised death rate in 2017, the low SDI quintile (0·52 [0·42–0·64]) had the second-highest rate. The largest decrease in age-standardised death rate from 1990 to 2017 occurred in the middle SDI quintile (46·2% [51·8–22·4] decrease; GBD results tool).

**Figure 10:**
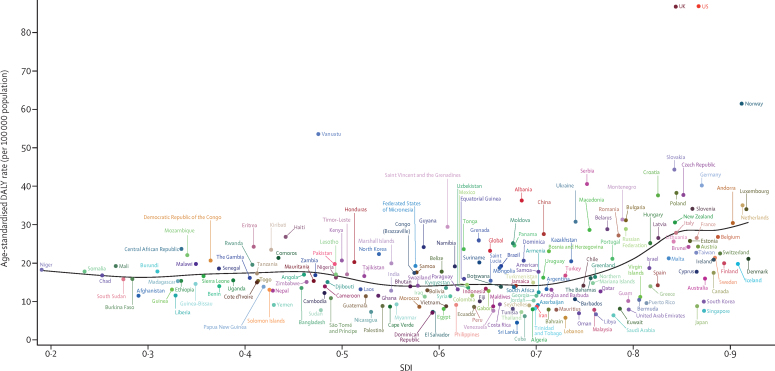
Age-standardised DALYs rates from IBD by SDI for 195 countries and territories in 2017 The black line represents the expected age-standardised DALY rate of IBD based solely on SDI. DALY=disability-adjusted life-year. IBD=inflammatory bowel disease. SDI=Socio-demographic Index.

The age-standardised prevalence rate of IBD in the two most populous countries, China and India, were 136·2 (95% UI 125·4 to 147·4) per 100 000 population and 16·2 (14·7 to 17·9) per 100 000 population, respectively, in 2017 (see appendix p 22–29 for the full national-level data for age-standardised prevalence rates). The highest age-standardised prevalence rate of IBD was observed in the USA (464·5 [438·6 to 490·9] per 100 000 population), followed by the UK (449·6 [420·6 to 481·6] per 100 000 population). At a national level, the largest change in age-standardised prevalence rate of IBD from 1990 to 2017 occurred in the Solomon Islands, where the age-standardised prevalence rate increased by 139·8% (106·3 to 177·7). In 1990, the age-standardised prevalence rate of IBD in this country was 10·3 (9·2 to 11·5) per 100 000 population, increasing to 24·6 (20·7 to 29·5) per 100 000 population in 2017. Vanuatu had the highest age-standardised death rate in 2017 (1·8 [0·8 to 3·2] per 100 000 population), and Singapore had the lowest (0·08 [0·06 to 0·14] per 100 000 population; appendix pp 17, 30–37; see appendix pp 30–37 for the full national level data for age-standardised death rates). At the national level, Italy had the largest increase in age-standardised death rate over the study period, from 0·40 (0·34 to 0·63) per 100 000 population in 1990 to 0·79 (0·34 to 0·95) per 100 000 population in 2017—a 99% (−39·4 to 170·6) increase. South Korea had the largest decrease in age-standardised death rate over the study period (77·8% [84·6 to 8·7] decrease per 100 000 population (appendix pp 18, 30–37). The age-standardised death rate in South Korea decreased from 1·62 (0·48 to 2·07) per 100 000 population in 1990 to 0·36 (0·30 to 0·49) per 100 000 population in 2017. Age-standardised DALY rates caused by IBD in 2017, for 195 countries and territories, are presented in figure 10 and in the appendix (pp 38–45).

## Discussion

In this study, we used a standardised approach to describe burden due to IBD at the global, super-region, regional, and national levels. We report that currently, approximately nearly 3·9 million females and nearly 3·0 million males are living with IBD worldwide and the number of prevalent cases is on the rise. This is important for health-care delivery systems and economies in the global context of treating chronic diseases like IBD, because standard care for these conditions, particularly immunotherapies, is extremely costly.

Historically, IBD has been considered a condition of high-income countries.^1^, ^10^ We found that the region of high-income North America, specifically the USA, makes a prominent contribution to the global number of patients with IBD. The USA had the highest age-standardised prevalence rate globally, with nearly a quarter of total global patients with IBD living there in 2017. Among European countries, the UK had the highest age-standardised prevalence. The prevalence of IBD was reported to range from 252 to 439 cases per 100 000 population in the USA.^11^ A 2018 systematic review^3^ evaluating more than 200 population-based studies reported that the highest prevalence rate of IBD occurred in North America. In the UK, a prevalence as high as 373 per 100 000 population has been reported.^12^ Our estimates confirmed these findings and showed an age-standardised prevalence rate of 464·5 (95% UI 438·6–490·9) per 100 000 population for the USA.

We also noticed a clear trend in prevalence of IBD from low to high SDI quintiles, with higher prevalence in countries in the high SDI quintile. This pattern was preserved over time, suggesting that the burden of IBD was consistently greater in countries with a high index of development such as the UK, the USA, Canada, and Australia. This correlation, suggested by many studies,^13^, ^14^, ^15^ might indicate that there are common environmental pressures across these regions that act as important risk factors for IBD, although we did not evaluate the role of potential risk factors in IBD prevalence for this study. These risk factors might include urbanisation, more hygienic environments, and diets low in dietary fibre and high in meat.^15^, ^16^ Based on the immune disease development model, the higher prevalence of IBD among people with higher socioeconomic status has been suggested to be due to a delay in or low level of exposure to common infectious agents during childhood. Consequently, the immune response is altered in genetically susceptible individuals.^17^, ^18^ Therefore, the primary health-care indicators of high-income and low-income countries might be correlated with prevalence rates, because in many low-income countries in Asia and Africa, basic sanitation is still an issue. The higher prevalence of IBD in regions with higher SDI might also be interpreted as an indication that individuals with higher socio-demographic status are at higher risk of IBD and need more investigation during their routine check-ups. Another possible explanation for the higher prevalence of IBD in higher sociodemographic regions might be better access to diagnostic testing tools, resulting in higher rates of diagnosis.^16^

We report an increase in age-standardised prevalence rate of IBD in regions that formerly had low prevalence, including east and south Asia, Oceania, and sub-Saharan Africa. It is probable that a combination of factors, including improvements in the socioeconomic status of newly industrialised countries, changes in diet and other lifestyle changes, improved sanitation, changed microbiota, and environmental factors, increase the risk of developing IBD.^10^, ^13^, ^19^ Behavioural and environmental factors might play an increasingly critical role in the development of IBD.^20^ Different factors that might increase the risk of developing IBD include smoking, lifestyle choices, discontinued breastfeeding, enteric infections, appendicectomy, and air pollution.^20^, ^21^ Improvement in access to health-care systems, more widely available diagnostic tools, and increased awareness on the part of both patients and physicians^19^ might also contribute to higher rates of diagnosis.

The continuing increase in prevalence of IBD in previously low-prevalence areas has important implications for both health-care providers and those responsible for health-care policy planning. Almost three-quarters of all people (about 3–5 billion) live in developing countries.^22^ Almost 2·7 billion people live in India and China. Therefore, even a small increase in the occurrence of chronic diseases such as IBD, which has low mortality but high disability, could have devastating effects in developing countries in the coming years.^3^ Of note, the economic effects of IBD are not limited to its burden on health-care systems. A German study^23^ reported that, each year, about 9% and 3% of all German employees with IBD had rehabilitation or were granted a disability pension, respectively. More than 50% of the total social costs of IBD are indirect costs such as early retirement or sick leave.^24^ Therefore, IBD contributes to an ever-increasing burden, not only on health-care systems but also on the economy as a whole.

We report that the age-specific death rate at the global and regional levels attenuated from 1990 to 2017. The overall decrease in mortality probably reflects the improved survival of patients with IBD, which might be due to increased use of immunomodulators, earlier introduction of biological agents, improvements in surgical techniques, and increased awareness of colorectal cancer surveillance.^25^, ^26^

Because the prevalence of IBD is much lower in low SDI countries, we expected a lower age-standardised death rate in these countries than in high SDI locations. However, the low quality of death registries in these countries might be another reason behind the low number of reported deaths in low SDI countries. Moreover, it is clear that IBD is not an easy disease to diagnose—ie, it requires a colonoscopy, and this is not available for the majority of people living in low SDI countries. It is possible that the fewer reported deaths due to IBD in low SDI countries might be due to under-detection of IBD-related deaths.

Although the fatal burden of IBD remains relatively low, the non-fatal burden continues to increase, climbing from the fifth-leading cause of YLDs among digestive diseases in 1990 to the fourth in 2017. IBD can substantially compromise the physical, psychological, familial, and social dimensions of life. As a result, the secondary effects of the disease can be seen in the increased rates of anxiety, depression, and other emotional effects. A 2016 study^27^ showed a notable association between symptoms of depression and clinical disease activity in patients with IBD, regardless of IBD subtypes. However, only IBD-specific symptoms are accounted for in disability weights in GBD, and not the social stigma, depression, anxiety, and other inflammatory conditions.

Sex-stratified global and national incidence rates of IBD, reported either by GBD or by other studies, are similar, suggesting that the disease is not sex-specific.^28^ However, the age-standardised death rate of IBD is lower, but the prevalence rate is higher, among females. It is possible that differences in environmental determinants derived from biological, social, and economic exposures between males and females might be responsible for this difference. The higher prevalence of smoking, as one of the most consistently studied environmental factors of IBD, in males compared with females might have contributed to the higher mortality rate in male patients.^29^ Alternatively, some studies point to the influence of hormones on the brain–gut–microbiota axis as the reason for sex differences in IBD prognosis, but the mechanism underlying this complex pathophysiology is still not completely understood.

As is the case with all GBD research, our study was limited by low availability and quality of data, which could only partially be overcome by using statistical methods. In data-scarce locations, we had to rely on predictive covariates and spatiotemporal trends. For non-fatal models, for which data were especially scarce, this resulted in estimates for some regions being determined entirely by global trends and associations with income and health-care access (in the case of Oceania and western, central, and southern sub-Saharan Africa), and by these factors plus data for a single country (in the case of eastern sub-Saharan Africa and South Asia). This is reflected in wider UIs in these locations, and suggests extra caution should be applied in interpreting estimates for these locations. It might also explain an unexpected discrepancy in age-standardised prevalence rate between China (136·2 [95% UI 125·4–147·4] per 100 000 population), where we had many data inputs, and India (16·2 [14·7–17·9] per 100 000 population) in 2017, where estimates were heavily influenced by a single Nepalese data source. Our results for India were lower than previous reports, mainly for the northern parts of the country. The age-standardised prevalence rate estimates for India also contrast previous reports,^30^, ^31^, ^32^ suggesting that a single Nepalese study in the GBD 2017 database for south Asia may be poorly representative of the region. Primary data from these previous reports should be incorporated into future rounds of GBD to improve these estimates. Likewise, special effort should be made to obtain more input data from other data-scarce regions.

Additionally, some prevalence estimates could have been improved by imposing an upper bound on the prior value for excess mortality rate or providing data inputs on excess mortality to our compartmental models. For example, a large population-based study^33^ in Canada reported a prevalence of IBD around 520 per 100 000 population, notably higher than the GBD 2017 prevalence estimates, which ranged from 46 to 57 cases per 100 000 population; this lower estimate was influenced by a high previous distribution of excess mortality in our high-income North America model, which was overcome by abundant prevalence data inputs for the USA, but not by the relatively fewer prevalence data inputs for Canada.

Lastly, because of greater availability of data from facilities and insurance claims, we relied on ICD10-based diagnosis for our case definition, and used fixed effects from our global mixed-effects compartmental model to adjust data with other case definitions toward this reference. In future rounds of GBD, we will use previously published estimates of the predictive value of ICD codes in administrative data to adjust our administrative data inputs toward the magnitude we would expect with stringent diagnosis, thus improving the specificity of our case definition but maintaining the geographical coverage afforded by administrative data. We also hope to include an analysis of the burden of IBD that is attributable to certain risk factors in future iterations of GBD, once the links between IBD and potential risk factors are better understood.

The natural course of IBD, with low mortality, as well as improved survival, caused an increase in prevalence of the disease from 1990 to 2017. In keep with this trend, prevalence is expected to continue increasing in the future. Rising prevalence, plus the increase in incidence in historically low-incidence regions, will have important health and economic effects. Our findings could be useful for health service planners and policy makers to justify and prioritise resource allocation to be able to respond to the growing number of patients with IBD. This study will motivate health planners to develop cost-effective and simple community-based interventions for implementation by health-care professionals at the primary-care level. This is necessary because IBD can last for many years and the ageing population is increasing.

We emphasise that understanding the shared and different environmental determinants of IBD across various regions is essential to implement interventions that will slow down the rising global burden of IBD.
