# Supplementary material for: The global, regional, and national burden of inflammatory bowel disease in 195 countries and territories, 1990–2017: a systematic analysis for the Global Burden of Disease Study 2017
Source: Lancet Gastroenterol Hepatol. 2019 Oct 21;5(1):17–30. doi: 10.1016/S2468-1253(19)30333-4 (PMC7026709; doi:10.1016/S2468-1253(19)30333-4)
Supplement: Supplementary appendix [file mmc1.pdf]

THE LANCET
Gastroenterology & Hepatology

Supplementary appendix

This appendix formed part of the original submission and has been peer reviewed. We post it as supplied by the authors.

Supplement to: GBD 2017 Inflammatory Bowel Disease Collaborators. The global, regional, and national burden of inflammatory bowel disease in 195 countries and territories, 1990–2017: a systematic analysis for the Global Burden of Disease Study 2017. *Lancet Gastroenterol Hepatol* 2019; published online Oct 21. http://dx.doi.org/10.1016/S2468-1253(19)30333-4.

Supplementary appendix

Supplement to: **Global, regional, and national burden of inflammatory bowel disease in 195 countries and territories, 1990–2017: a systematic analysis for the Global Burden of Disease Study 2017**

Contents:

Appendix Figures

Appendix figure 1: Age patterns by sex in 2017 of the total number of deaths and age-specific death rates of IBD at the global level

Appendix figure 2: Age patterns by sex in 2017 of the total number and age-specific YLDs rates of IBD at the global level. IBD=inflammatory bowel disease. YLDs=years lived with disability.

Appendix figure 3: Age patterns by sex in 2017 of the total number and age-specific YLLs rates of IBD at the global level. IBD=inflammatory bowel disease. YLLs=years of life lost.

Appendix Figure 4: Trends from 1990 to 2017 for number and age-standardised DALY rates of IBD at the global level. IBD=inflammatory bowel disease. DALYs=disability-adjusted life-years.

Appendix figure 5: Age patterns by sex in 2017 for number and age-standardised DALYs rates of IBD at the global level. IBD=inflammatory bowel disease. DALYs=disability-adjusted life-years.

Appendix figure 6: Trends from 1990 to 2017 for age-standardised prevalence rate of IBD across Global Burden of Disease super-regions. IBD=inflammatory bowel disease.

Appendix Figure 7: Trends from 1990 to 2017 for age-standardised DALY rates of IBD across Global Burden of Disease super-regions. IBD=inflammatory bowel disease. DALYs=disability-adjusted life-years.

Appendix figure 8: Trends from 1990 to 2017 in number of prevalent cases of IBD for both sexes, across 21 regions. IBD=inflammatory bowel disease.

Appendix Figure 9: Age-standardised prevalence rates of IBD by sex, across 21 regions, in 2017. IBD=inflammatory bowel disease.

Appendix figure 10: Percentage change in age-standardised prevalence rates of IBD by sex, across 21 regions, from 1990 to 2017. IBD=inflammatory bowel disease.

Appendix figure 11: Age-standardised death rates from IBD by sex, across 21 regions, in 2017. IBD=inflammatory bowel disease.

Appendix figure 12: Percentage change in age-standardised death rates from IBD by sex, across 21 regions, from 1990 to 2017. IBD=inflammatory bowel disease.

Appendix Figure 13: Age-standardised DALYs rates from IBD by sex, across 21 regions, in 2017. IBD=inflammatory bowel disease. DALYs=disability-adjusted life-years.

Appendix Figure 14: Trend from 1990 to 2017 in number of DALYs from IBD for both sexes, across 21 regions. IBD=inflammatory bowel disease. DALYs=disability-adjusted life-years.

Appendix figure 15: Age-standardised death rates (per 100 000) from IBD for both sexes 2017 at the country level. IBD=inflammatory bowel disease.

Appendix figure 16: Percentage change in age-standardised death rates (per 100 000), from IBD for both sexes, at the country level, from 1990 to 2017. IBD=inflammatory bowel disease.

Appendix figure 17: Age-standardised DALYs rates (per 100 000), from IBD for both sexes 2017 at the country level. IBD=inflammatory bowel disease. DALYs=disability-adjusted life-years.

Appendix figure 18: Percentage change in age-standardised DALY rates (per 100 000), from IBD for both sexes, at the country level, from 1990 to 2017. IBD=inflammatory bowel disease. DALYs=disability-adjusted life-years.

Appendix Tables

Appendix Table 1: Data points by site for IBD. IBD=inflammatory bowel disease.

Appendix table 2: IBD prevalence in 1990 and 2017 for both sexes and percentage change in age-standardised rates by location

Appendix Table 3: Deaths due to IBD in 1990 and 2017 for both sexes, and percentage change in age-standardised rates by location. IBD=inflammatory bowel disease.

Appendix Table 4: DALYs due to IBD in 1990 and 2017 for both sexes, and percentage change of age-standardised rates by location. IBD=inflammatory bowel disease. DALYs=disability-adjusted life-years.

Appendix figure 1: Age patterns by sex in 2017 of the total number of deaths and age-specific death rates of IBD at the global level

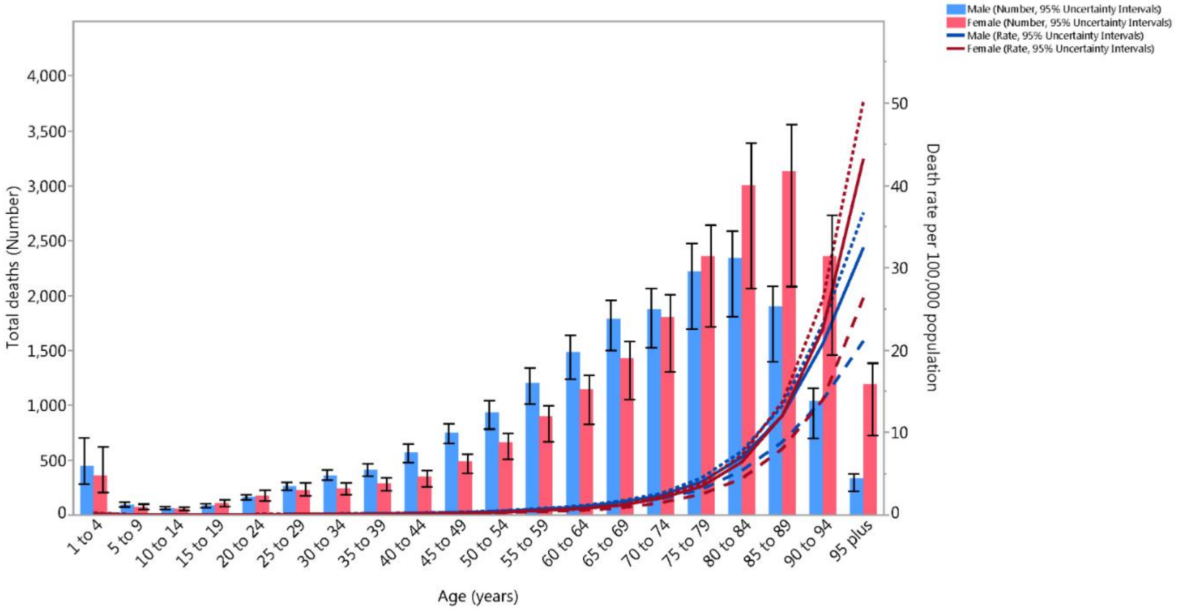

Dotted and dashed lines indicate 95% upper and lower uncertainty intervals, respectively. IBD=inflammatory bowel disease.

Appendix figure 2: Age patterns by sex in 2017 of the total number and age-specific YLDs rates of IBD at the global level

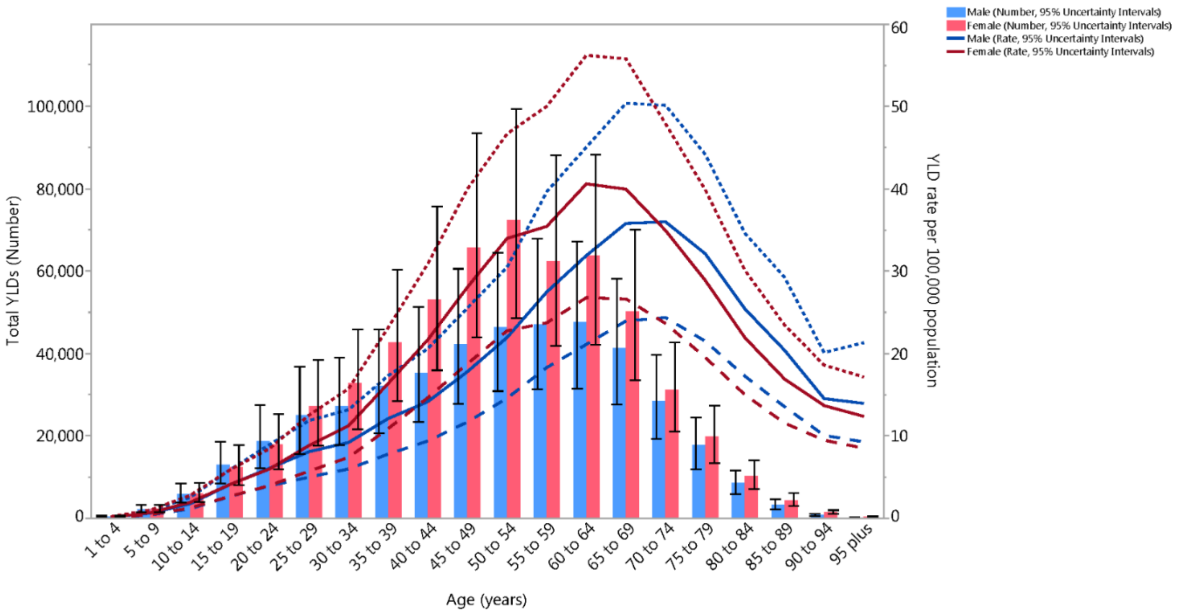

Dotted and dashed lines indicate 95% upper and lower uncertainty intervals, respectively. IBD=inflammatory bowel disease. YLDs=years lived with disability.

Appendix figure 3: Age patterns by sex in 2017 of the total number and age-specific YLLs rates of IBD at the global level

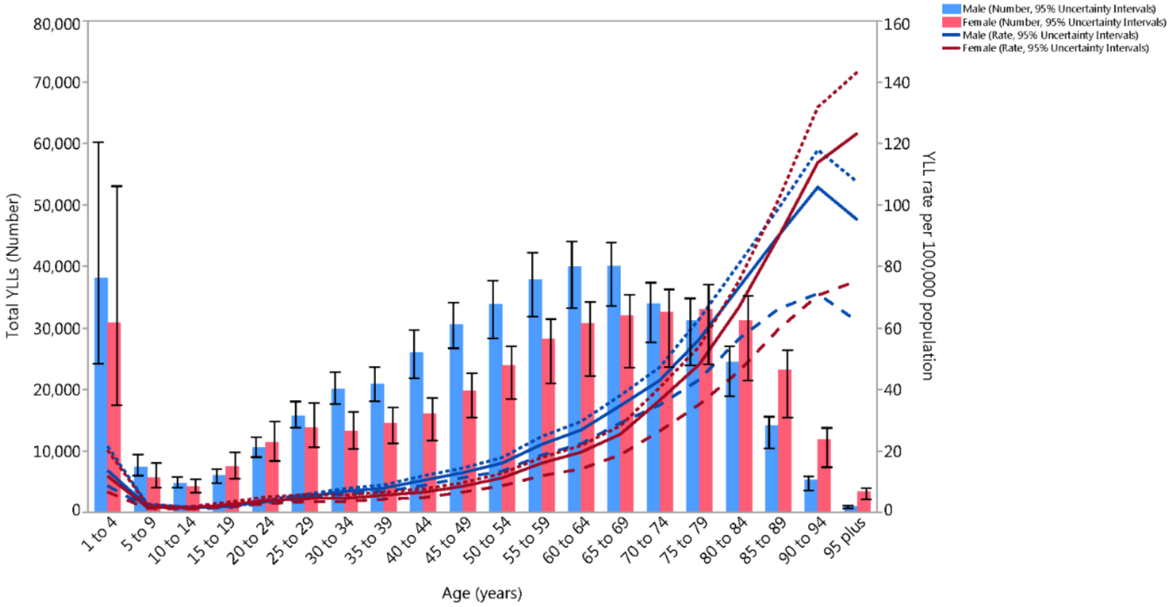

Dotted and dashed lines indicate 95% upper and lower uncertainty intervals, respectively. IBD=inflammatory bowel disease. YLLs=years of life lost.

Appendix figure 4: Trends from 1990 to 2017 for number and age-standardised DALY rates of IBD at the global level

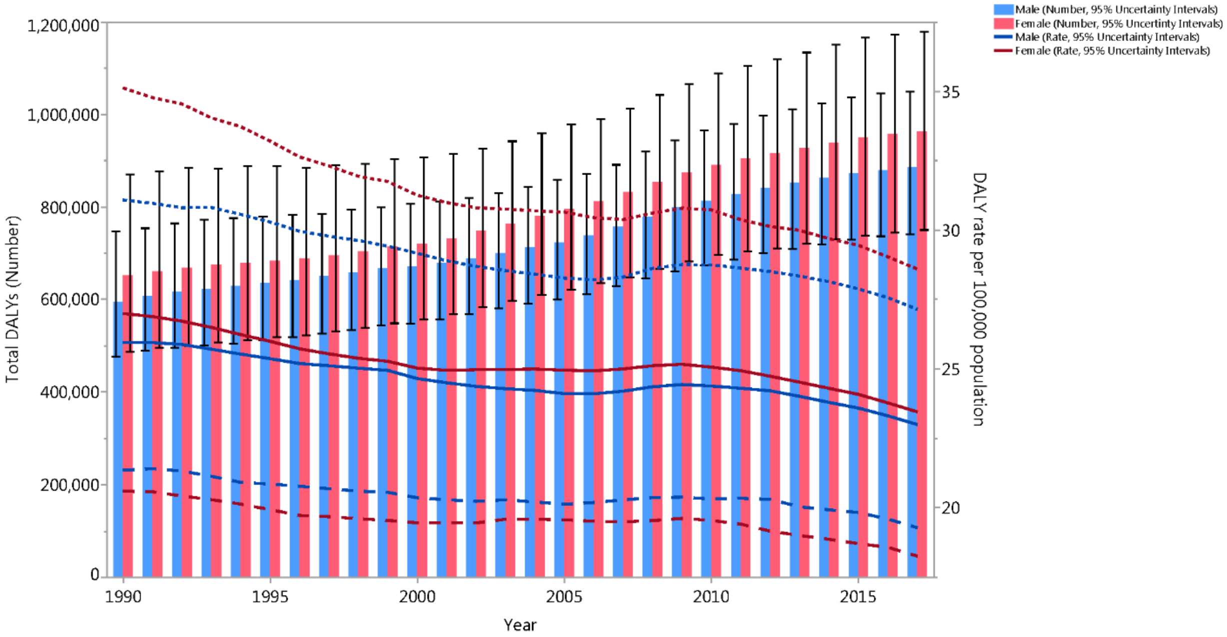

Dotted and dashed lines indicate 95% upper and lower uncertainty intervals, respectively. IBD=inflammatory bowel disease. DALYs=disability-adjusted life-years.

Appendix figure 5: Age patterns by sex in 2017 for number and age-standardised DALYs rates of IBD at the global level

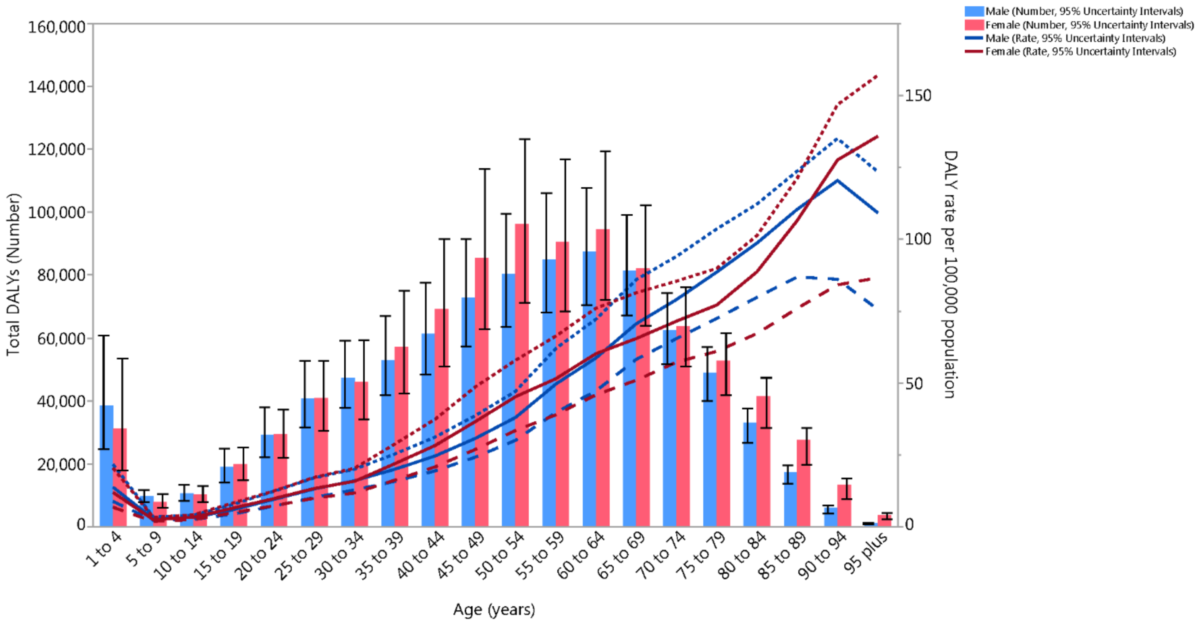

Dotted and dashed lines indicate 95% upper and lower uncertainty intervals, respectively. IBD=inflammatory bowel disease. DALYs=disability-adjusted life-years.

Appendix figure 6: Trends from 1990 to 2017 for age-standardised prevalence rate of IBD across Global Burden of Disease super-regions

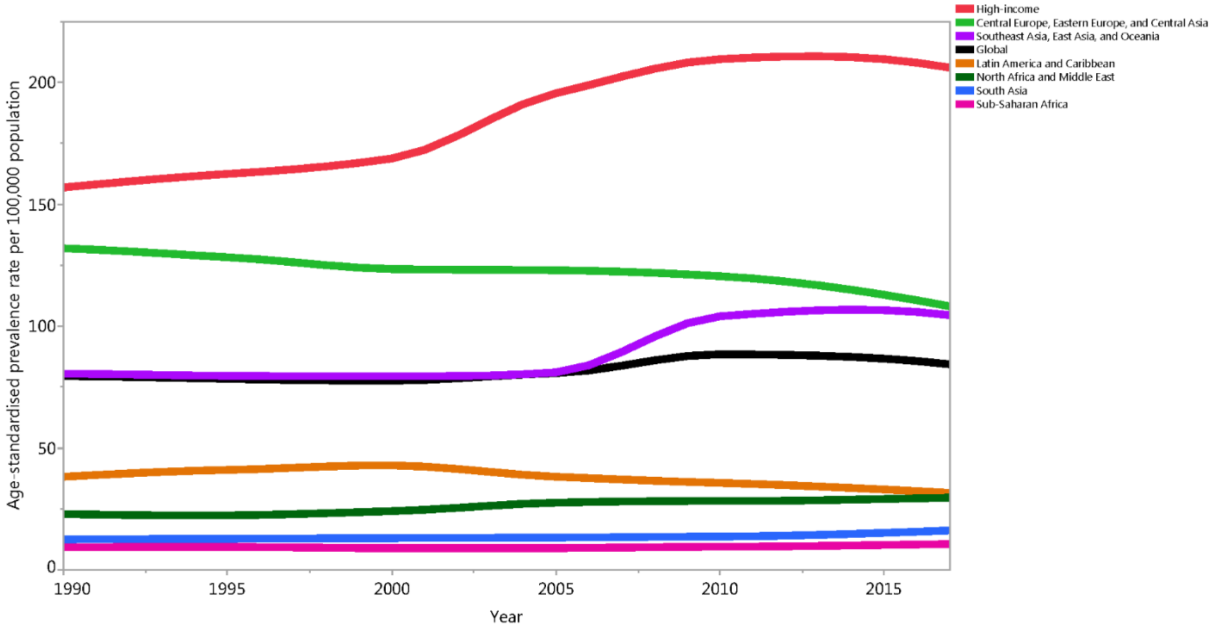

IBD=inflammatory bowel disease.

Appendix Figure 7: Trends from 1990 to 2017 for age-standardised DALY rates of IBD across Global Burden of Disease super-regions

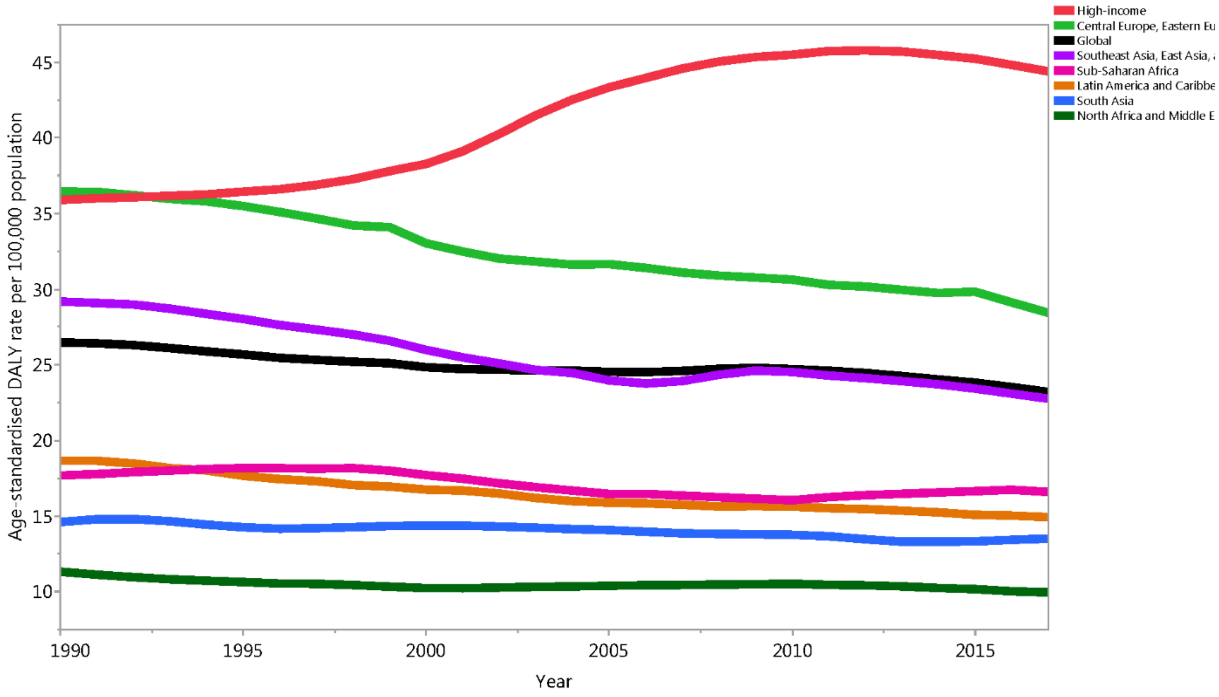

IBD= inflammatory bowel disease. DALYs=disability-adjusted life-years.

Appendix figure 8: Trends from 1990 to 2017 in number of prevalent cases of IBD for both sexes, across 21 regions

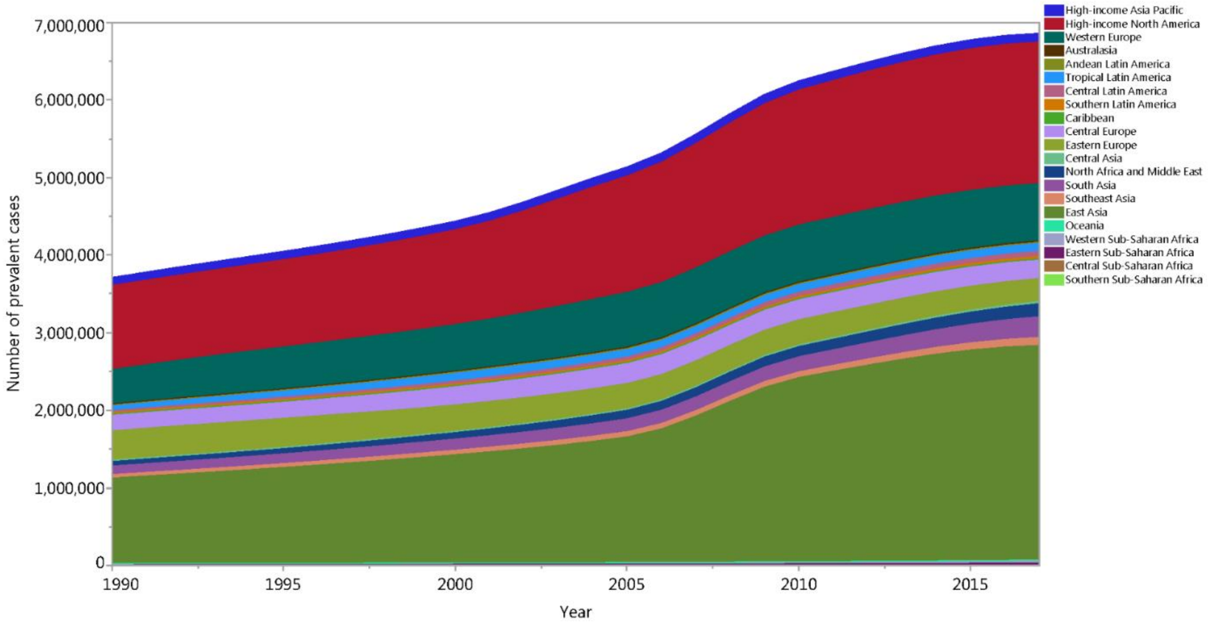

IBD=inflammatory bowel disease.

Appendix Figure 9: Age-standardised prevalence rates of IBD by sex, across 21 regions, in 2017

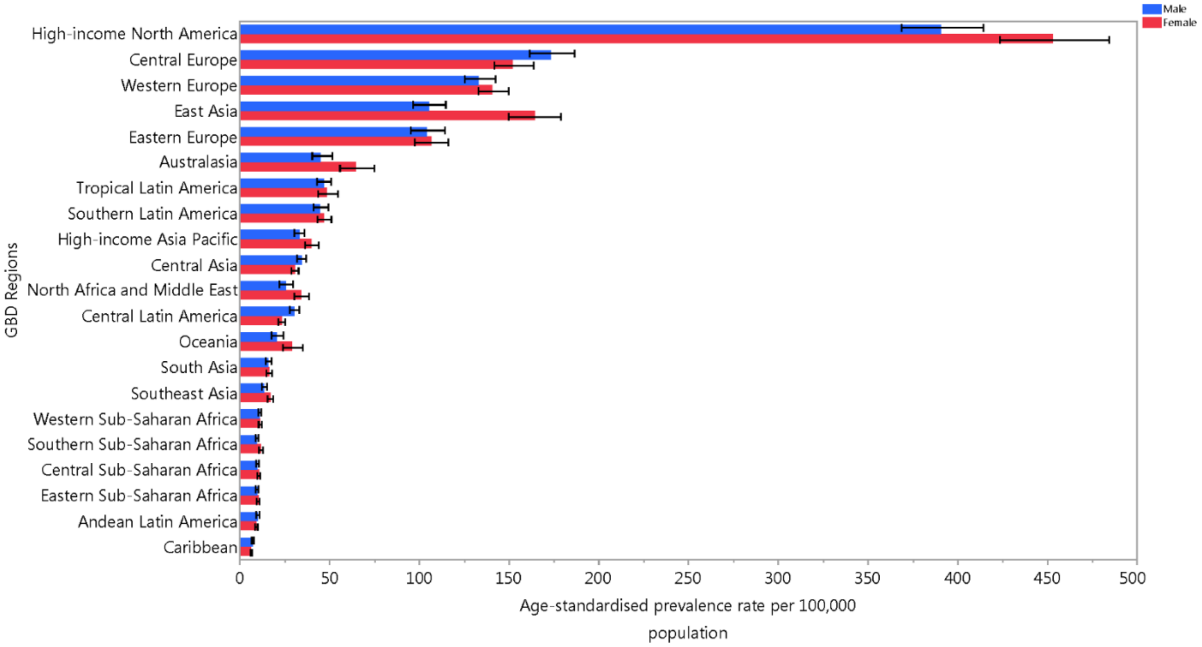

IBD=inflammatory bowel disease.

Appendix figure 10: Percentage change in age-standardised prevalence rates of IBD by sex, across 21 regions, from 1990 to 2017

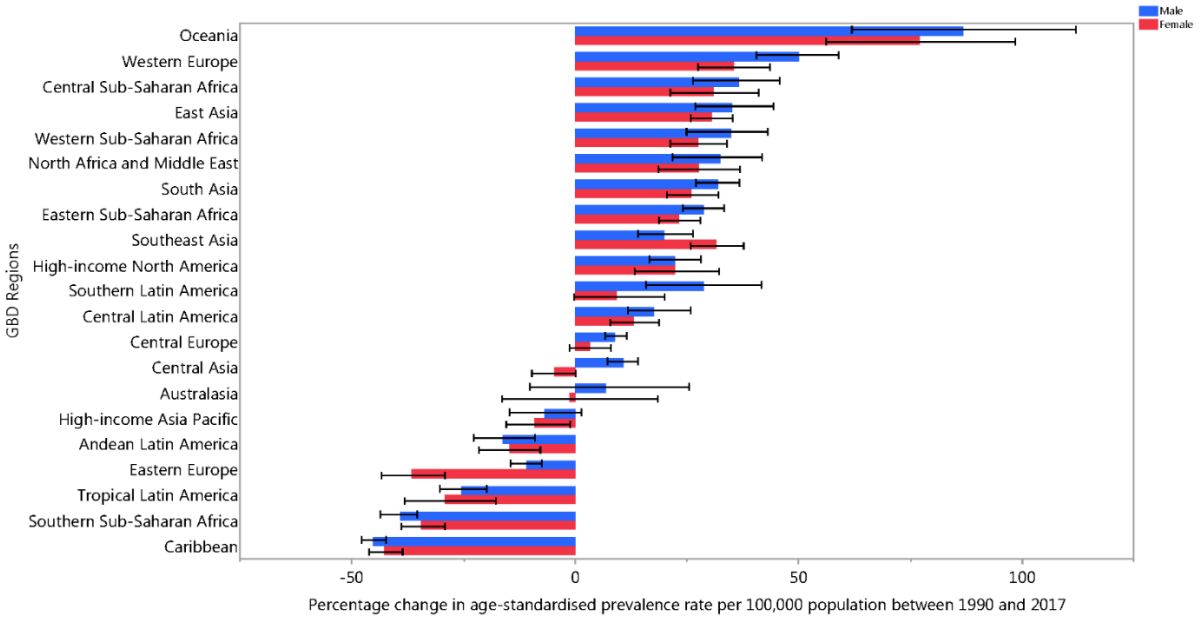

IBD=inflammatory bowel disease.

Figure 11: Age-standardised death rates from IBD by sex, across 21 GBD regions, in 2017

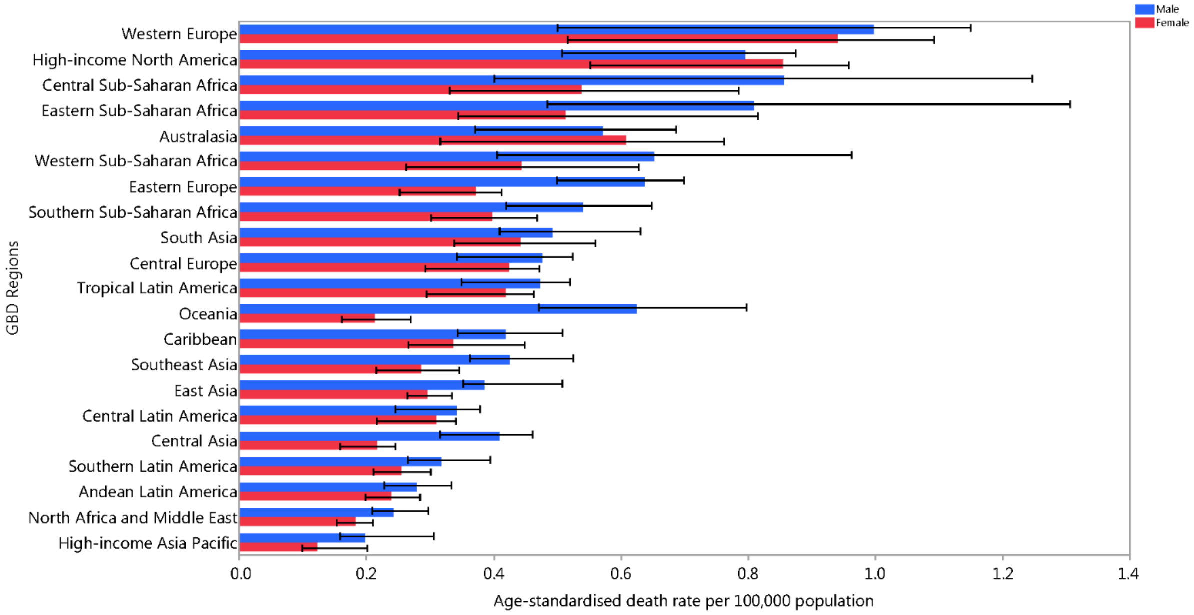

IBD=inflammatory bowel disease.

Appendix figure 12: Percentage change in age-standardised death rates from IBD by sex, across 21 GBD regions, from 1990 to 2017

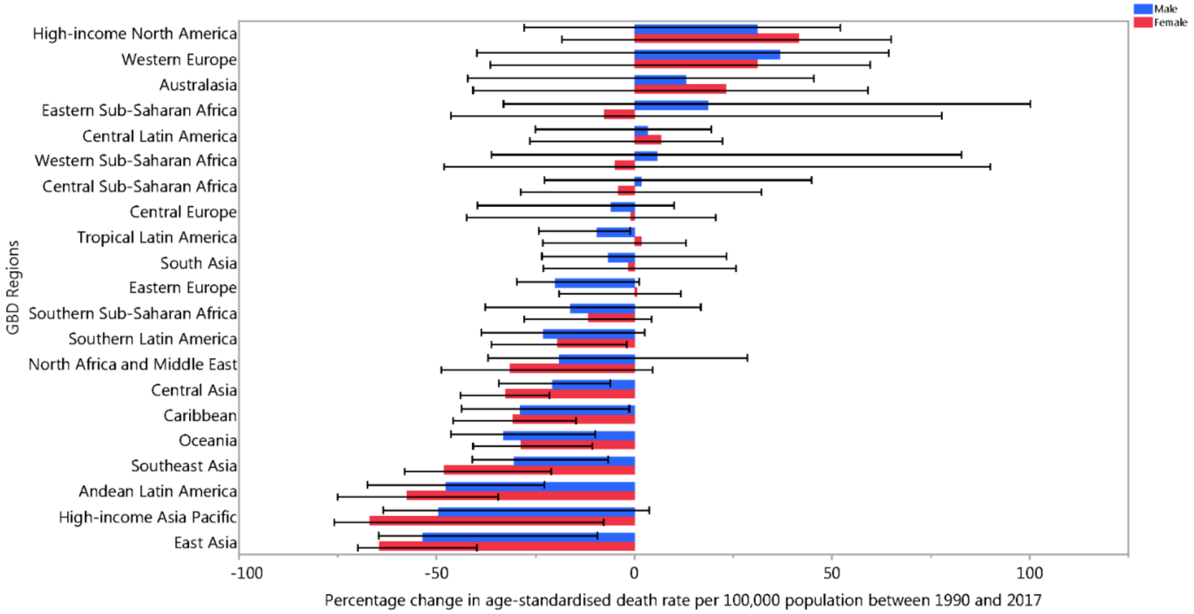

IBD=inflammatory bowel disease.

Appendix Figure 13: Age-standardised DALYs rates from IBD by sex, across 21 GBD regions, in 2017

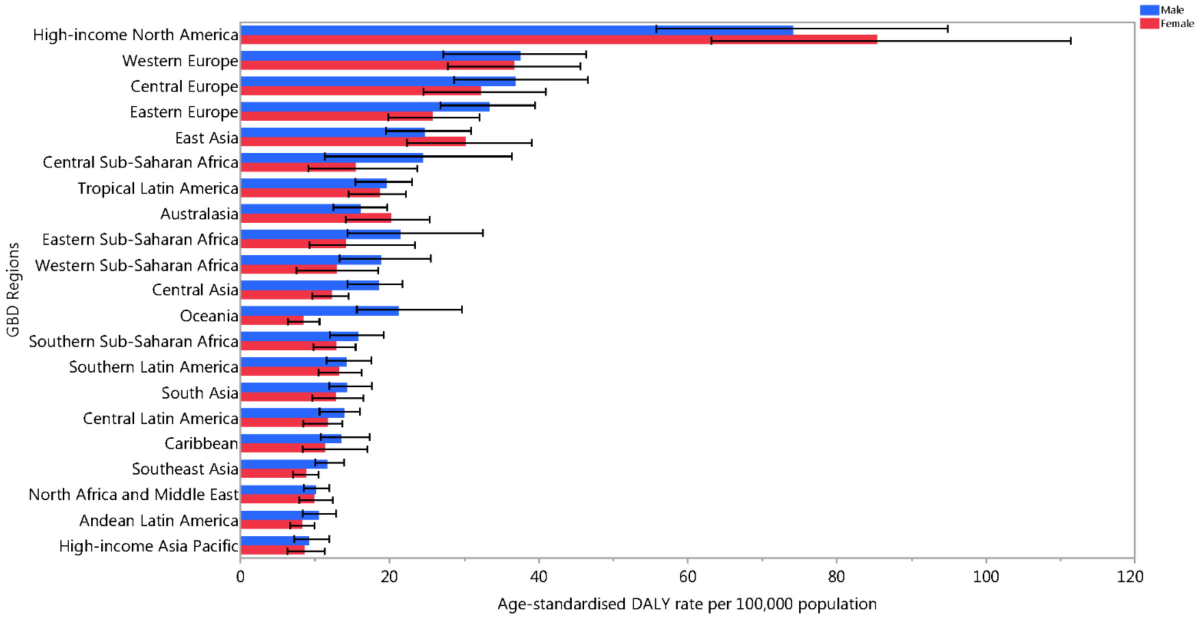

IBD=inflammatory bowel disease. DALYs=disability-adjusted life-years.

Appendix Figure 14: Trend from 1990 to 2017 in number of DALYs from IBD for both sexes, across 21 regions

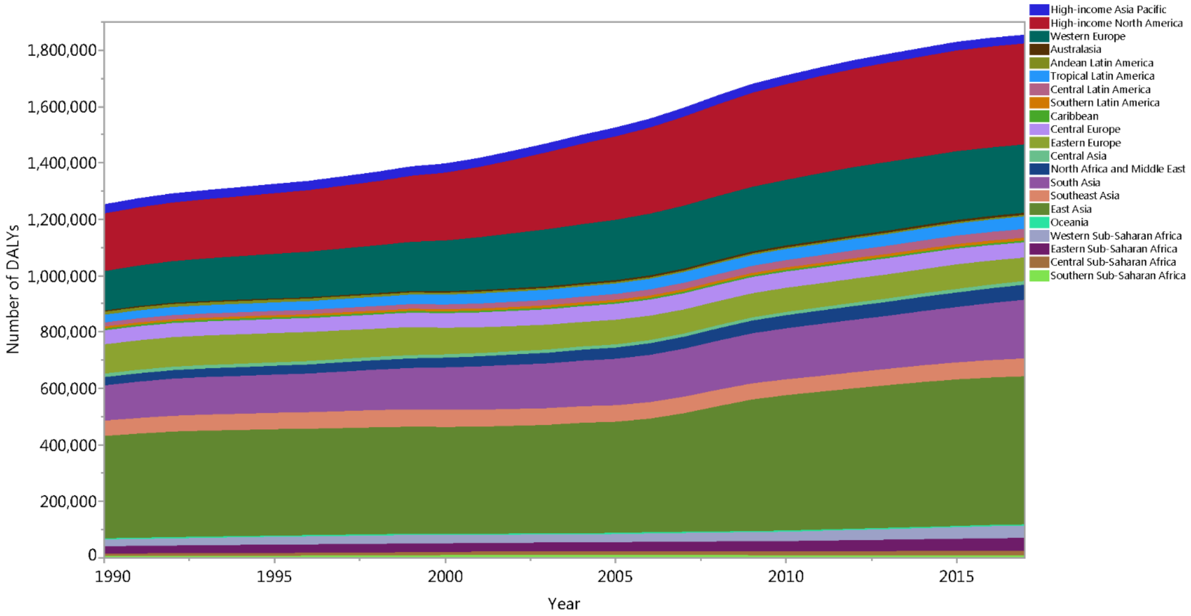

IBD=inflammatory bowel disease. DALYs=disability-adjusted life-years.

Appendix figure 15: Age-standardised death rates (per 100 000) from IBD for both sexes 2017 at the country level

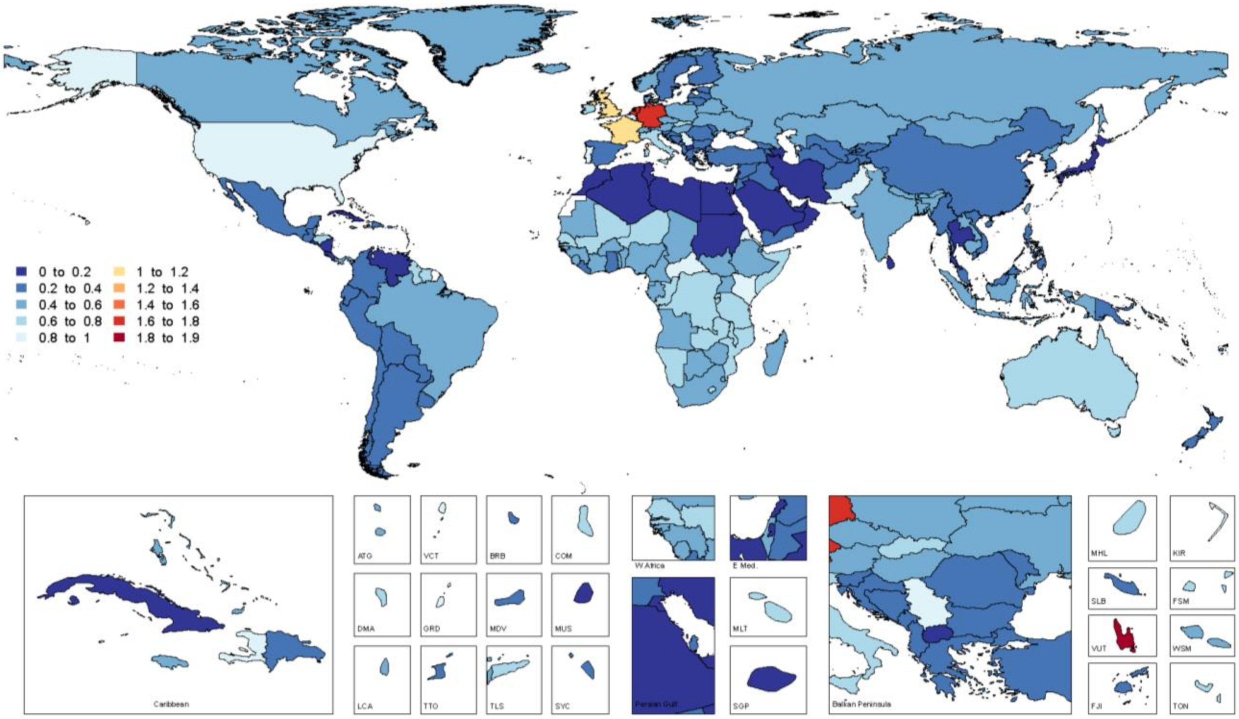

IBD=inflammatory bowel disease. ATG=Antigua and Barbuda, VCT=Saint Vincent and the Grenadines, BRB=Barbados, COM=Comoros, DMA=Dominica, GRD=Grenada, MDV=Maldives, MUS=Mauritius, LCA=Saint Lucia, TTO=Trinidad and Tobago, TLS=Timor-Leste, SYC=Seychelles, MLT=Malta, SGP=Singapore, MHL=Marshall Islands, KIR=Kiribati, SLB=Solomon Islands, FSM=Federated States of Micronesia, VUT=Vanuatu, WSM=Samoa, FJI=Fiji, TON=Tonga.

Appendix figure 16: Percentage change in age-standardised death rates (per 100 000) from IBD for both sexes, at the country level, from 1990 to 2017

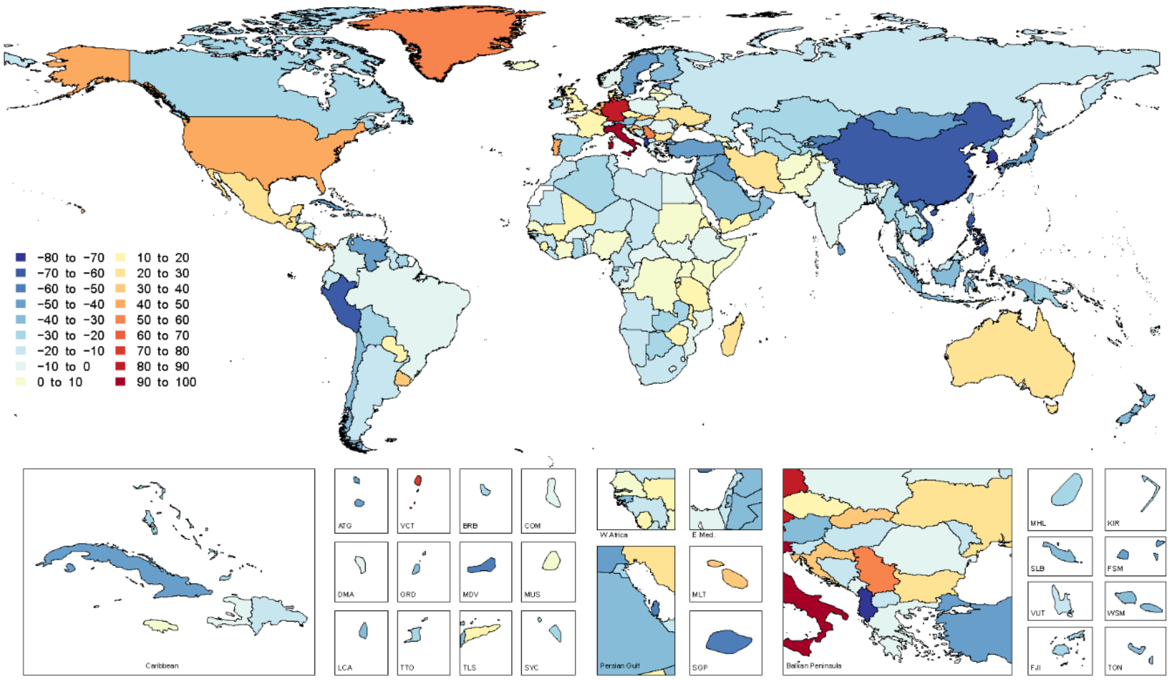

IBD=inflammatory bowel disease. ATG=Antigua and Barbuda, VCT=Saint Vincent and the Grenadines, BRB=Barbados, COM=Comoros, DMA=Dominica, GRD=Grenada, MDV=Maldives, MUS=Mauritius, LCA=Saint Lucia, TTO=Trinidad and Tobago, TLS=Timor-Leste, SYC=Seychelles, MLT=Malta, SGP=Singapore, MHL=Marshall Islands, KIR=Kiribati, SLB=Solomon Islands, FSM=Federated States of Micronesia, VUT=Vanuatu, WSM=Samoa, FJI=Fiji, TON=Tonga.

Appendix figure 17: Age-standardised DALYs rates (per 100 000) from IBD for both sexes 2017 at the country level

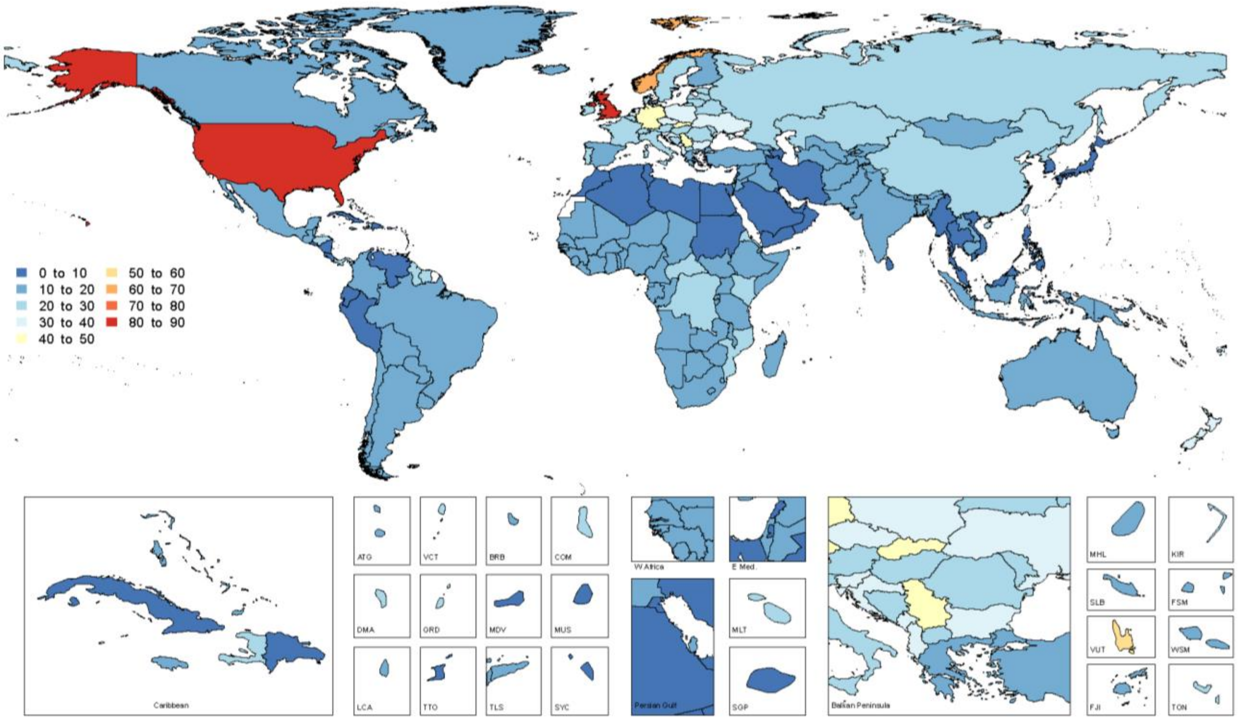

IBD=inflammatory bowel disease. DALYs=disability-adjusted life-years. ATG=Antigua and Barbuda, VCT=Saint Vincent and the Grenadines, BRB=Barbados, COM=Comoros, DMA=Dominica, GRD=Grenada, MDV=Maldives, MUS=Mauritius, LCA=Saint Lucia, TTO=Trinidad and Tobago, TLS=Timor-Leste, SYC=Seychelles, MLT=Malta, SGP=Singapore, MHL=Marshall Islands, KIR=Kiribati, SLB=Solomon Islands, FSM=Federated States of Micronesia, VUT=Vanuatu, WSM=Samoa, FJI=Fiji, TON=Tonga.

Appendix figure 18: Percentage change in age-standardised DALY rates (per 100 000) from IBD for both sexes, at the country level, from 1990 to 2017

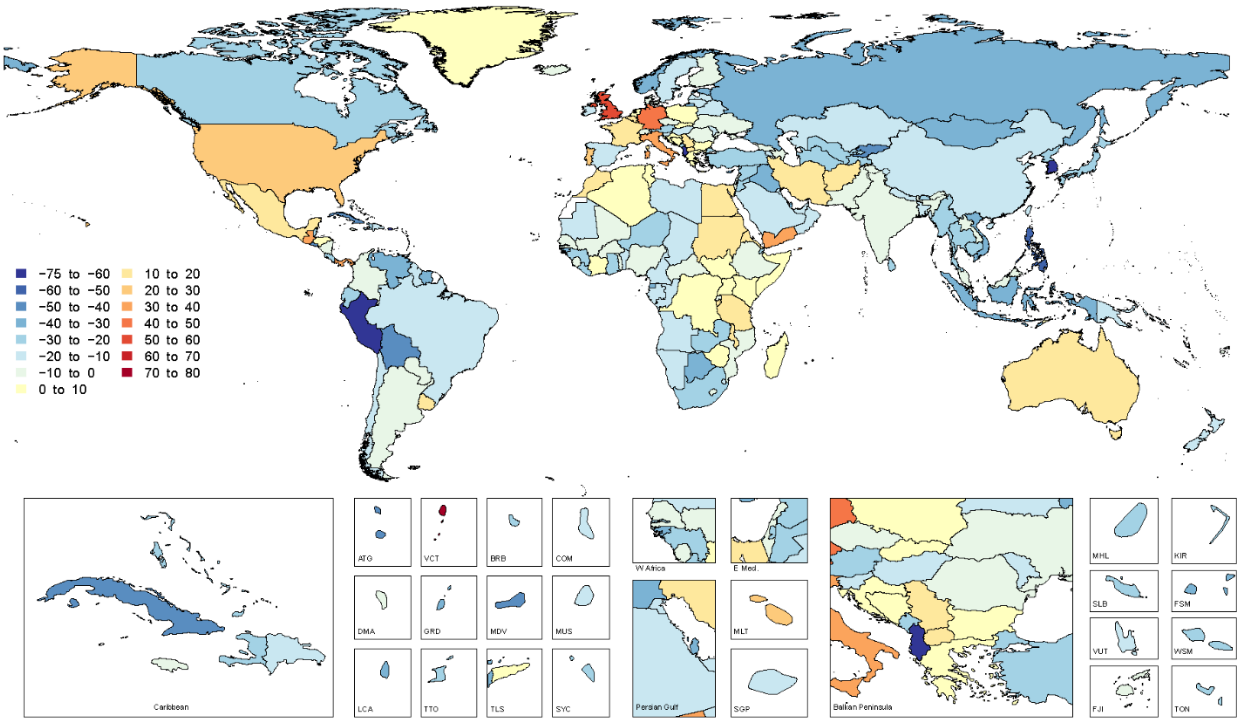

IBD=inflammatory bowel disease. DALYs=disability-adjusted life-years. ATG=Antigua and Barbuda, VCT=Saint Vincent and the Grenadines, BRB=Barbados, COM=Comoros, DMA=Dominica, GRD=Grenada, MDV=Maldives, MUS=Mauritius, LCA=Saint Lucia, TTO=Trinidad and Tobago, TLS=Timor-Leste, SYC=Seychelles, MLT=Malta, SGP=Singapore, MHL=Marshall Islands, KIR=Kiribati, SLB=Solomon Islands, FSM=Federated States of Micronesia, VUT=Vanuatu, WSM=Samoa, FJI=Fiji, TON=Tonga.

Appendix Table 1. Data points for ulcerative colitis and Crohn's disease

1a: Non-infective inflammatory bowel disease due to ulcerative colitis.

	Prevalence	Incidence	Case fatality
**Site-years (total)**	1,241	282	3
**Number of countries with data**	51	52	2
**Number of GBD regions with data (out of 21)**	17	13	1
**Number of GBD super-regions with data (out of 7 super-regions**	7	6	1

1b: Non-infective inflammatory bowel disease due to Crohn's disease

	Prevalence	Incidence	Case fatality
**Site-years (total)**	1,183	270	1
**Number of countries with data**	50	49	1
**Number of GBD regions with data (out of 21)**	17	12	1
**Number of GBD super-regions with data (out of 7 super-regions**	7	5	1

Appendix table 2: IBD prevalence in 1990 and 2017 for both sexes and percentage change in age-standardised rates by location

	1990		2017		Percentage change in age-standardized rates between 1990 and 2017
	Counts (95% UI)	Rate (95% UI)	Counts (95% UI)	Rate (95% UI)	
**Global**	3699934 (3536764, 3888255)	79.5 (75.9, 83.5)	6848872 (6421390, 7304419)	84.3 (79.2, 89.9)	6.1 (3.3, 8.6)
**High-income North America**	1085638 (1044639, 1132603)	344.8 (331.7, 359.3)	1824322 (1727288, 1921648)	422.0 (398.7, 446.1)	22.4 (16.3, 28.7)
**Canada**	17538 (16014, 19554)	57.1 (52.3, 63.4)	20360 (18722, 22566)	46.6 (42.8, 51.9)	-18.4 (-26.9, -9.1)
**Greenland**	29 (26, 32)	54.1 (48.9, 60.5)	32 (28, 38)	47.8 (42.5, 56.1)	-11.6 (-23.6, 4.3)
**USA**	1068048 (1027131, 1114359)	376.1 (362.0, 392.0)	1803898 (1707622, 1900996)	464.5 (438.6, 490.9)	23.5 (17.2, 29.9)
**Australasia**	11920 (10861, 13095)	53.8 (48.9, 59.1)	18490 (16596, 20688)	54.9 (49.5, 61.0)	2.0 (-9.9, 16.0)
**Australia**	6638 (6067, 7426)	36.1 (33, 40.8)	10680 (9686, 12000)	37.9 (34.3, 43.2)	5.1 (-8.1, 19.2)
**New Zealand**	5282 (4412, 6250)	142.9 (120.4, 168.4)	7810 (6544, 9492)	148.2 (123.9, 178.9)	3.7 (-18.9, 29.2)
**High-income Asia-Pacific**	78971 (72854, 86056)	39.7 (36.7, 43.1)	89447 (82801, 96633)	36.4 (33.7, 39.3)	-8.2 (-13.4, -2.8)
**Brunei**	44 (37, 53)	19.6 (16.4, 23.1)	157 (125, 201)	34.3 (27.8, 43.3)	74.5 (43.8, 115.9)
**Japan**	67049 (61423, 73627)	44.4 (40.8, 48.5)	66300 (60739, 72594)	39.2 (35.9, 42.9)	-11.7 (-17.5, -5.8)
**Singapore**	869 (796, 953)	25.8 (23.6, 28.3)	2514 (2296, 2772)	36.9 (33.8, 40.5)	43.0 (30.9, 58.0)
**South Korea**	11009 (10199, 11874)	25.2 (23.4, 27.1)	20476 (18536, 22545)	30.3 (27.5, 34.0)	20.4 (10.3, 34.6)
**Western Europe**	441120 (419869, 464651)	95.9 (91.3, 101.1)	738083 (698523, 781879)	136.6 (129.6, 144.4)	42.5 (35.7, 48.9)
**Andorra**	97 (69, 136)	153.7 (109.0, 215.3)	127 (86, 189)	114.7 (79.6, 163.7)	-25.3 (-43.2, -2.0)
**Austria**	7022 (6075, 8295)	75.6 (65.7, 88.7)	9233 (7819, 11041)	83.6 (70.1, 101.2)	10.6 (-11.4, 36.4)
**Belgium**	9581 (8667, 10891)	79.0 (71.4, 89.0)	13464 (12321, 15286)	93.9 (86.4, 104.9)	18.9 (3.9, 34.4)
**Cyprus**	416 (370, 476)	50.7 (45.1, 57.8)	962 (873, 1078)	61.1 (55.6, 68.8)	20.5 (2.8, 38.5)
**Denmark**	3934 (3435, 4684)	63.9 (56.3, 75.7)	4479 (3904, 5343)	64.2 (56.4, 76.1)	0.5 (-16, 18.4)
**Finland**	5039 (4163, 6411)	84.0 (70.3, 106.0)	6345 (5140, 8648)	91.7 (75.3, 126.8)	9.2 (-18.9, 57.3)
**France**	40496 (36098, 47145)	61.0 (54.4, 69.8)	53375 (48654, 60318)	66.6 (60.3, 75.8)	9.3 (-6.0, 26.7)
**Germany**	62158 (55870, 71967)	63.2 (56.8, 73.2)	97652 (90233, 105262)	88.3 (81.7, 97.1)	39.8 (21.4, 55.7)
**Greece**	7120 (6585, 7795)	57.6 (53.1, 63.1)	9302 (8466, 10399)	68.7 (62.8, 76.4)	19.2 (7.9, 34.4)
**Iceland**	190 (172, 218)	71.6 (64.8, 82.9)	269 (241, 319)	66.8 (60.1, 78.7)	-6.6 (-19.9, 10.5)
**Ireland**	2550 (2284, 2988)	68.9 (61.9, 80.6)	4072 (3606, 4799)	71 (63.4, 82.9)	3.0 (-13.7, 21.6)
**Israel**	2796 (2434, 3293)	58.9 (51.1, 69.4)	6591 (6081, 7259)	70.9 (65.5, 77.7)	20.4 (1.9, 38.4)
**Italy**	56469 (51123, 65077)	80.9 (72.9, 94.4)	76581 (70764, 83027)	93.8 (86.8, 102.3)	16.0 (-0.2, 28.9)

**Luxembourg**	447<br>(410, 503)	94.7<br>(87.1, 106.4)	831<br>(756, 949)	110.9<br>(100.9, 125.7)	17.2<br>(4.0, 34.9)
**Malta**	219<br>(198, 255)	53.2<br>(48.1, 60.9)	373<br>(341, 425)	66.3<br>(60.7, 74.7)	24.5<br>(9.4, 40.8)
**Netherlands**	12524<br>(11237, 14369)	72.2<br>(64.9, 82.5)	14672<br>(12985, 17544)	69.6<br>(61.4, 85.9)	-3.6<br>(-18.3, 18.1)
**Norway**	20340<br>(16210, 25635)	409.5<br>(327.7, 511.9)	17791<br>(13584, 23505)	274.4<br>(213.1, 354.0)	-33.0<br>(-51.9, -8.4)
**Portugal**	4321<br>(3779, 5044)	36.2<br>(32.2, 42.1)	6657<br>(6007, 7682)	47.3<br>(42.7, 53.9)	30.5<br>(11.6, 51.2)
**Spain**	19834<br>(17001, 24403)	43.4<br>(37.4, 52.6)	29186<br>(26398, 32764)	48.7<br>(44.4, 54.4)	12.3<br>(-5.8, 32.2)
**Sweden**	10413<br>(9155, 12393)	99.1<br>(87.7, 115.7)	12231<br>(10520, 15016)	98.4<br>(85.4, 118.0)	-0.7<br>(-16.4, 20.5)
**Switzerland**	7764<br>(7208, 8548)	91.8<br>(85.4, 101.2)	10626<br>(9775, 12084)	94.8<br>(87.1, 109.2)	3.3<br>(-6.8, 18.4)
**United Kingdom**	166965<br>(157784, 177224)	244.7<br>(230.5, 260.4)	362500<br>(338481, 391401)	449.6<br>(420.6, 481.6)	83.7<br>(72.1, 96.3)
**Southern Latin America**	18692<br>(17219, 20282)	38.8<br>(35.7, 42.0)	33575<br>(31210, 36409)	45.6<br>(42.5, 49.3)	17.6<br>(9.4, 26.1)
**Argentina**	11959<br>(10847, 13249)	36.7<br>(33.3, 40.6)	21163<br>(19583, 23187)	43.7<br>(40.6, 47.8)	19.3<br>(7.9, 31.3)
**Chile**	5282<br>(4779, 5850)	43.6<br>(39.5, 48.3)	10584<br>(9629, 11770)	50.0<br>(45.5, 55.5)	14.8<br>(4.1, 27.8)
**Uruguay**	1451<br>(1338, 1591)	42.4<br>(39.1, 46.4)	1827<br>(1682, 2011)	45.0<br>(41.4, 49.5)	6.2<br>(-1.2, 16.0)
**Eastern Europe**	377954<br>(346486, 412059)	143.7<br>(131.7, 156.6)	296246<br>(270939, 322578)	104.5<br>(96.1, 113.8)	-27.3<br>(-32.6, -21.7)
**Belarus**	15643<br>(13542, 17925)	129.8<br>(113.0, 148.1)	14918<br>(12805, 17611)	115.9<br>(99.1, 135.8)	-10.7<br>(-19.0, -1.8)
**Estonia**	2893<br>(2660, 3140)	155.5<br>(143.0, 168.3)	2064<br>(1909, 2233)	111.5<br>(103.7, 120.1)	-28.3<br>(-33.5, -23.3)
**Latvia**	4301<br>(3938, 4684)	131.4<br>(120.3, 142.7)	3132<br>(2900, 3400)	111.9<br>(103.7, 120.6)	-14.8<br>(-21.4, -7.8)
**Lithuania**	5889<br>(5358, 6472)	137.0<br>(125.2, 150.0)	4491<br>(4146, 4849)	111.7<br>(103.1, 122.3)	-18.5<br>(-25.5, -11.5)
**Moldova**	4709<br>(4370, 5061)	102.3<br>(94.8, 110)	4856<br>(4502, 5235)	99.4<br>(92.7, 106.4)	-2.8<br>(-9.3, 3.2)
**Russia**	258284<br>(232383, 284795)	149.1<br>(134.4, 164.7)	197640<br>(179945, 216250)	101.2<br>(92.8, 110.1)	-32.1<br>(-38.7, -25.5)
**Ukraine**	86236<br>(77679, 95752)	135.4<br>(122.5, 149.8)	69145<br>(62566, 76575)	112.3<br>(101.5, 124.3)	-17.0<br>(-24.9, -8.2)
**Central Europe**	211813<br>(196617, 227208)	151.9<br>(141.7, 162.7)	249563<br>(231791, 268715)	161.5<br>(151.0, 173.0)	6.3<br>(3.7, 9.2)
**Albania**	4497<br>(3703, 5496)	162.9<br>(134.4, 198.1)	5871<br>(4693, 7253)	168.8<br>(135.8, 208.8)	3.6<br>(-8.7, 15.3)
**Bosnia and Herzegovina**	4833<br>(4469, 5235)	103.4<br>(95.9, 111.3)	5407<br>(5006, 5817)	117.6<br>(109.6, 125.5)	13.7<br>(9.0, 19.1)
**Bulgaria**	15428<br>(12632, 18793)	140.4<br>(115.6, 170.5)	15688<br>(12533, 19401)	155.8<br>(125.7, 192.6)	11.0<br>(0.7, 21.2)
**Croatia**	11156<br>(10282, 12102)	191.2<br>(176.7, 207.6)	11410<br>(10651, 12277)	194.2<br>(181.5, 208.0)	1.6<br>(-5.8, 8.3)
**Czech Republic**	22327<br>(20683, 24065)	186.1<br>(172.5, 199.7)	24029<br>(22563, 25792)	168.7<br>(158.9, 180.0)	-9.3<br>(-13.9, -5.0)
**Hungary**	11011<br>(10233, 11840)	89.0<br>(82.5, 95.6)	10467<br>(9721, 11260)	80.6<br>(75.2, 86.4)	-9.4<br>(-14.7, -3.9)
**Macedonia**	2656<br>(2152, 3271)	128.5<br>(104.9, 157.1)	4547<br>(3635, 5655)	161.8<br>(129.6, 201.9)	25.9<br>(13.3, 37.0)
**Montenegro**	1356<br>(1079, 1688)	208.2<br>(166.1, 259.6)	1310<br>(1050, 1627)	163.5<br>(131.3, 204.5)	-21.5<br>(-29.7, -12.5)
**Poland**	72792<br>(67488, 78845)	170.0<br>(158.4, 183.3)	97335<br>(90624, 104919)	189.7<br>(177.3, 203.8)	11.6<br>(6.0, 17.8)

**Romania**	34677<br>(32140, 37265)	131.9<br>(122.5, 141.6)	37512<br>(34902, 40152)	140.2<br>(131.2, 149.5)	6.3<br>(1.8, 10.9)
**Serbia**	15096<br>(14065, 16247)	140.2<br>(131.3, 150.3)	18095<br>(16903, 19450)	156.2<br>(146.1, 166.9)	11.4<br>(6.2, 17.8)
**Slovakia**	11058<br>(10259, 11888)	193.1<br>(179.6, 207.1)	12499<br>(11593, 13526)	176.1<br>(163.6, 189.9)	-8.8<br>(-13.9, -2.7)
**Slovenia**	4926<br>(4564, 5304)	214.2<br>(198.7, 229.9)	5392<br>(5037, 5800)	189.3<br>(177.3, 203.1)	-11.6<br>(-16.6, -6.3)
**Central Asia**	18291<br>(17248, 19419)	31.4<br>(29.7, 33.3)	28765<br>(26875, 30799)	32.3<br>(30.2, 34.5)	2.8<br>(-0.7, 6.3)
**Armenia**	1384<br>(1271, 1500)	42.5<br>(39.3, 46.0)	1352<br>(1247, 1466)	37.2<br>(34.4, 40.1)	-12.5<br>(-18.2, -6.9)
**Azerbaijan**	1864<br>(1731, 1999)	29.3<br>(27.2, 31.4)	3680<br>(3411, 3966)	33.5<br>(31.2, 36.0)	14.5<br>(9.7, 19.5)
**Georgia**	2105<br>(1939, 2280)	35.2<br>(32.5, 38.2)	1665<br>(1548, 1799)	36.1<br>(33.7, 39.0)	2.5<br>(-5.7, 10.4)
**Kazakhstan**	5845<br>(5385, 6319)	38.0<br>(35.1, 41.0)	5777<br>(5332, 6260)	31.3<br>(29, 33.9)	-17.5<br>(-23.2, -11.8)
**Kyrgyzstan**	1093<br>(1010, 1184)	29.8<br>(27.6, 32.1)	1792<br>(1664, 1925)	31.3<br>(29.1, 33.6)	5.3<br>(-1.9, 12.6)
**Mongolia**	336<br>(307, 370)	21.4<br>(19.5, 23.5)	917<br>(849, 990)	29.7<br>(27.7, 31.9)	39.0<br>(24.8, 55.1)
**Tajikistan**	866<br>(803, 935)	22.6<br>(21.0, 24.3)	2495<br>(2317, 2683)	32.9<br>(30.5, 35.4)	45.9<br>(35.0, 56.7)
**Turkmenistan**	700<br>(624, 788)	25.7<br>(22.8, 28.8)	1520<br>(1406, 1649)	31.9<br>(29.5, 34.4)	24.0<br>(9.7, 39.7)
**Uzbekistan**	4098<br>(3810, 4382)	26.4<br>(24.6, 28.3)	9568<br>(8903, 10340)	31.8<br>(29.5, 34.2)	20.4<br>(13.2, 27.5)
**Central Latin America**	24723<br>(22953, 26510)	23.2<br>(21.6, 25.0)	65310<br>(60586, 70561)	26.7<br>(24.8, 28.9)	15.2<br>(11.2, 20.0)
**Colombia**	4873<br>(4457, 5507)	22.0<br>(20.1, 24.5)	14058<br>(12650, 16464)	26.2<br>(23.7, 30.6)	19.5<br>(6.6, 37.9)
**Costa Rica**	1233<br>(1144, 1329)	58.5<br>(54.2, 63.2)	1487<br>(1323, 1743)	29.8<br>(26.6, 35.1)	-49.0<br>(-53.3, -40.8)
**El Salvador**	531<br>(458, 640)	15.4<br>(13.2, 19.0)	1361<br>(1234, 1545)	23.8<br>(21.5, 27.1)	53.8<br>(25.1, 80.0)
**Guatemala**	538<br>(476, 629)	11.3<br>(9.9, 13.1)	2777<br>(2505, 3191)	22.7<br>(20.4, 26.2)	101.2<br>(69.1, 138.0)
**Honduras**	330<br>(300, 373)	12.2<br>(10.9, 13.8)	1572<br>(1426, 1804)	22.7<br>(20.6, 26.1)	86.7<br>(61.7, 114.0)
**Mexico**	13023<br>(11911, 14184)	23.6<br>(21.6, 25.8)	33915<br>(30966, 37031)	27.9<br>(25.5, 30.4)	18.1<br>(13.8, 22.4)
**Nicaragua**	400<br>(370, 445)	19.1<br>(17.6, 21.1)	1384<br>(1256, 1576)	27.1<br>(24.6, 30.7)	41.8<br>(28.4, 63.1)
**Panama**	490<br>(454, 534)	27.9<br>(25.8, 30.8)	1161<br>(1016, 1403)	29.1<br>(25.6, 34.9)	4.3<br>(-6.9, 24.3)
**Venezuela**	3305<br>(3027, 3635)	26.5<br>(24.3, 29.3)	7593<br>(6900, 8689)	25.3<br>(23.1, 28.9)	-4.4<br>(-13.1, 8.0)
**Andean Latin America**	2921<br>(2739, 3130)	11.3<br>(10.5, 12.1)	5433<br>(5057, 5832)	9.5<br>(8.9, 10.2)	-15.4<br>(-19.9, -10.5)
**Bolivia**	308<br>(285, 333)	7.3<br>(6.7, 7.9)	770<br>(715, 833)	7.9<br>(7.3, 8.6)	8.4<br>(-0.2, 16.5)
**Ecuador**	791<br>(727, 878)	11.7<br>(10.7, 13.0)	1641<br>(1509, 1796)	10.5<br>(9.7, 11.5)	-9.7<br>(-19.9, 1.3)
**Peru**	1822<br>(1690, 1968)	12.2<br>(11.3, 13.2)	3022<br>(2783, 3293)	9.5<br>(8.8, 10.4)	-21.6<br>(-27.1, -15.0)
**Caribbean**	3582<br>(3325, 3853)	12.0<br>(11.1, 13.0)	3329<br>(3099, 3576)	6.7<br>(6.3, 7.2)	-44.0<br>(-46.4, -41.1)
**Antigua and Barbuda**	7<br>(6, 7)	12.0<br>(11.0, 13.0)	8<br>(7, 8)	7.8<br>(7.1, 8.6)	-35.0<br>(-39.5, -29)
**The Bahamas**	17<br>(16, 20)	8.6<br>(7.8, 9.8)	26<br>(24, 29)	6.7<br>(6.1, 7.4)	-21.8<br>(-31.8, -13.5)

**Barbados**	24<br>(22, 26)	9.1<br>(8.3, 9.9)	29<br>(26, 32)	7.4<br>(6.8, 8.3)	-18.0<br>(-25.4, -7.8)
**Belize**	8<br>(7, 8)	5.9<br>(5.4, 6.4)	22<br>(20, 24)	6.5<br>(6.0, 7.2)	11.1<br>(2.9, 21.4)
**Bermuda**	14<br>(13, 15)	22.1<br>(20.3, 23.8)	7<br>(7, 9)	8.4<br>(7.4, 9.9)	-62.0<br>(-66.2, -55.5)
**Cuba**	2214<br>(2028, 2419)	20.8<br>(19.0, 22.7)	1148<br>(1045, 1267)	7.6<br>(7.0, 8.2)	-63.7<br>(-65.9, -60.9)
**Dominica**	5<br>(5, 6)	7.9<br>(7.3, 8.6)	6<br>(5, 6)	6.9<br>(6.4, 7.6)	-12.4<br>(-17.3, -5.7)
**Dominican Republic**	299<br>(278, 322)	5.9<br>(5.4, 6.3)	696<br>(637, 767)	7.0<br>(6.4, 7.8)	20.4<br>(12.3, 30.5)
**Grenada**	4<br>(4, 5)	5.9<br>(5.4, 6.4)	9<br>(8, 10)	6.7<br>(6.0, 7.7)	14.9<br>(5.6, 28.5)
**Guyana**	19<br>(18, 21)	3.2<br>(3.0, 3.5)	37<br>(34, 40)	5.4<br>(5.0, 5.9)	66.2<br>(52.9, 80.1)
**Haiti**	140<br>(130, 152)	2.9<br>(2.6, 3.2)	416<br>(388, 449)	4.7<br>(4.4, 5.1)	63.4<br>(48.0, 80.3)
**Jamaica**	193<br>(179, 209)	9.8<br>(9.0, 10.7)	207<br>(190, 228)	7.2<br>(6.6, 7.9)	-26.8<br>(-32.2, -20.1)
**Puerto Rico**	397<br>(363, 439)	10.8<br>(9.9, 12.0)	421<br>(372, 487)	8.3<br>(7.4, 9.6)	-23.2<br>(-32.9, -10.1)
**Saint Lucia**	7<br>(7, 8)	6.7<br>(6.1, 7.3)	14<br>(13, 16)	7.1<br>(6.4, 8.0)	6.0<br>(-2.7, 18.1)
**Saint Vincent and the Grenadines**	5<br>(5, 6)	6.1<br>(5.6, 6.6)	8<br>(8, 9)	6.5<br>(6.0, 7.1)	6.5<br>(-0.6, 15.0)
**Suriname**	17<br>(15, 18)	5.2<br>(4.8, 5.7)	38<br>(35, 43)	6.5<br>(5.9, 7.2)	23.3<br>(12.2, 37.7)
**Trinidad and Tobago**	73<br>(66, 80)	7.1<br>(6.5, 7.8)	106<br>(96, 119)	6.5<br>(5.9, 7.3)	-8.4<br>(-16.2, 1.9)
**Virgin Islands**	16<br>(14, 17)	15.9<br>(14.6, 17.3)	11<br>(10, 13)	7.7<br>(6.7, 9.2)	-51.6<br>(-57.2, -42.6)
**Tropical Latin America**	73744<br>(66784, 80487)	65.6<br>(59.3, 71.6)	110563<br>(101449, 120814)	47.5<br>(43.5, 51.7)	-27.7<br>(-33.5, -21.3)
**Brazil**	72271<br>(65303, 78974)	65.9<br>(59.5, 72.0)	107647<br>(98592, 117714)	47.4<br>(43.5, 51.7)	-28.1<br>(-33.9, -21.6)
**Paraguay**	1473<br>(1375, 1583)	54.5<br>(50.7, 59.0)	2916<br>(2714, 3153)	50.0<br>(46.5, 54.2)	-8.1<br>(-14.5, -1.3)
**East Asia**	1104158<br>(1033626, 1178136)	101.2<br>(94.6, 108.2)	2767618<br>(2529326, 3010003)	134.6<br>(123.9, 145.6)	33.0<br>(28.2, 37.9)
**China**	1047992<br>(981582, 1116181)	101.3<br>(94.7, 108.2)	2665081<br>(2433419, 2900745)	136.2<br>(125.4, 147.4)	34.5<br>(29.7, 39.6)
**North Korea**	19502<br>(16717, 22706)	100.6<br>(86.9, 117.1)	24177<br>(20778, 28001)	75.2<br>(65.0, 86.6)	-25.3<br>(-33.5, -15.9)
**Taiwan (Province of China)**	18269<br>(16568, 20486)	95.4<br>(86.5, 105.9)	33775<br>(29725, 39798)	97.7<br>(85.0, 117.0)	2.4<br>(-10.0, 21.7)
**Southeast Asia**	45189<br>(41401, 49233)	12.1<br>(11.1, 13.3)	103884<br>(93977, 114931)	15.3<br>(13.9, 16.8)	26.0<br>(21.3, 31.1)
**Cambodia**	653<br>(512, 831)	8.9<br>(6.9, 11.5)	2192<br>(1698, 2813)	15.3<br>(11.9, 19.7)	71.6<br>(45.3, 102.5)
**Indonesia**	14253<br>(13047, 15639)	9.5<br>(8.7, 10.3)	36572<br>(33260, 40456)	14.0<br>(12.8, 15.4)	48.3<br>(40.1, 56.2)
**Laos**	273<br>(215, 346)	8.9<br>(7.0, 11.6)	909<br>(691, 1217)	15.6<br>(12.1, 21.0)	75.1<br>(43.7, 114.4)
**Malaysia**	2069<br>(1896, 2282)	14.2<br>(13.1, 15.5)	5324<br>(4807, 6110)	17.4<br>(15.8, 20.0)	22.9<br>(11.2, 39.6)
**Maldives**	26<br>(20, 33)	17.6<br>(13.9, 22.3)	86<br>(65, 115)	19.3<br>(14.9, 24.7)	9.6<br>(-2.1, 25.3)
**Mauritius**	306<br>(239, 390)	31.4<br>(25.0, 39.8)	301<br>(232, 392)	19.1<br>(14.8, 24.5)	-39.4<br>(-47.1, -29.5)
**Myanmar**	3032<br>(2362, 3861)	9.1<br>(7.1, 11.6)	8335<br>(6280, 11046)	15.9<br>(12.1, 20.8)	73.5<br>(42.7, 108.7)

**Philippines**	5115<br>(4722, 5589)	10.9<br>(10.1, 12.0)	12111<br>(11183, 13067)	13.2<br>(12.2, 14.2)	20.9<br>(12.4, 29.4)
**Sri Lanka**	1807<br>(1673, 1953)	12.0<br>(11.1, 12.9)	3703<br>(3379, 4095)	15.1<br>(13.8, 16.6)	25.4<br>(18.3, 35.7)
**Seychelles**	16<br>(12, 20)	24.9<br>(19.6, 31.7)	23<br>(17, 31)	20.1<br>(15.2, 26.7)	-19.5<br>(-32.1, 4.4)
**Thailand**	6317<br>(5831, 6850)	12.7<br>(11.7, 13.7)	13718<br>(12487, 15395)	14.8<br>(13.5, 16.6)	16.9<br>(8.6, 28.7)
**East Timor**	55<br>(42, 71)	9.8<br>(7.5, 12.5)	166<br>(128, 212)	17<br>(13, 21.8)	74.4<br>(46.4, 107.2)
**Vietnam**	11209<br>(9579, 13191)	21.6<br>(18.4, 25.5)	20307<br>(17308, 24157)	19.8<br>(17, 23.3)	-8.2<br>(-17, 3.3)
**Oceania**	627<br>(565, 698)	13.6<br>(12.3, 15.1)	2302<br>(1936, 2743)	24.6<br>(20.9, 29.3)	81.3<br>(60.4, 102.8)
**American Samoa**	13<br>(12, 16)	38.1<br>(33.4, 44.2)	17<br>(14, 21)	32.5<br>(27.4, 38.8)	-14.8<br>(-26, -4.9)
**Federated States of Micronesia**	9<br>(8, 11)	13.3<br>(11.6, 15.5)	24<br>(20, 28)	26.3<br>(22.5, 31.6)	98.4<br>(65.8, 132.1)
**Fiji**	84<br>(75, 95)	14.7<br>(13.1, 16.7)	236<br>(197, 285)	26.8<br>(22.6, 31.9)	82<br>(64.2, 99.4)
**Guam**	63<br>(56, 72)	55.2<br>(48.0, 63.3)	64<br>(53, 77)	34.8<br>(29.2, 41.8)	-36.9<br>(-43.8, -28)
**Kiribati**	6<br>(5, 7)	10.7<br>(9.7, 11.9)	23<br>(19, 28)	25.5<br>(21.5, 30.8)	138.1<br>(104.6, 177.4)
**Marshall Islands**	4<br>(3, 4)	12.9<br>(11.3, 14.7)	12<br>(10, 14)	26.2<br>(22.2, 31.2)	103.7<br>(74.1, 133.6)
**Northern Mariana Islands**	21<br>(19, 24)	56.4<br>(49.7, 64.0)	20<br>(16, 25)	34.5<br>(29.1, 41.2)	-38.9<br>(-44.6, -31.4)
**Papua New Guinea**	296<br>(263, 336)	10.0<br>(8.8, 11.3)	1538<br>(1284, 1838)	23.6<br>(19.7, 28.2)	136.3<br>(100.1, 175.1)
**Samoa**	29<br>(26, 33)	25.0<br>(22.0, 28.5)	49<br>(42, 59)	30.7<br>(26.1, 36.3)	23.1<br>(9.8, 37.5)
**Solomon Islands**	23<br>(21, 26)	10.3<br>(9.2, 11.5)	111<br>(94, 133)	24.6<br>(20.7, 29.5)	139.8<br>(106.3, 177.7)
**Tonga**	24<br>(21, 27)	33.9<br>(29.8, 38.7)	27<br>(23, 32)	30.0<br>(25.4, 35.8)	-11.4<br>(-19.5, -1.7)
**Vanuatu**	12<br>(11, 14)	11.7<br>(10.4, 13.3)	55<br>(46, 66)	25.6<br>(21.5, 30.9)	118.5<br>(85.6, 153.2)
**North Africa and Middle East**	58503<br>(52019, 65412)	22.9<br>(20.4, 25.6)	166817<br>(146799, 191174)	29.6<br>(26.2, 33.8)	29.4<br>(21.5, 37.6)
**Afghanistan**	921<br>(740, 1159)	11.8<br>(9.4, 14.8)	4288<br>(3412, 5307)	20.7<br>(16.5, 25.7)	74.9<br>(44.9, 109.4)
**Algeria**	3841<br>(3106, 4689)	21.1<br>(17.0, 25.8)	12036<br>(9530, 15222)	29.7<br>(23.7, 37.6)	41.0<br>(20.0, 64.6)
**Bahrain**	163<br>(124, 211)	36.6<br>(28.9, 46.6)	532<br>(413, 696)	29.4<br>(23.4, 37.2)	-19.6<br>(-28.4, -10.2)
**Egypt**	7814<br>(6344, 9712)	17.9<br>(14.4, 22.4)	22797<br>(17992, 28809)	26.7<br>(21.2, 33.8)	48.9<br>(22.8, 77.4)
**Iran**	9677<br>(8774, 10688)	23.3<br>(21.2, 25.5)	23812<br>(21600, 26364)	27.0<br>(24.6, 29.6)	16.2<br>(6.0, 26.7)
**Iraq**	2538<br>(2033, 3110)	21.4<br>(17.1, 26.6)	9115<br>(7331, 11261)	26.2<br>(21.1, 32.2)	22.4<br>(6.5, 40.2)
**Jordan**	862<br>(706, 1077)	34.4<br>(28.2, 43.0)	2957<br>(2413, 3623)	31.4<br>(25.7, 38.7)	-8.6<br>(-19.1, 5.4)
**Kuwait**	480<br>(437, 539)	29.8<br>(27.3, 32.9)	1526<br>(1357, 1733)	30.9<br>(28.0, 34.8)	4.0<br>(-4.8, 15.5)
**Lebanon**	603<br>(558, 656)	18.5<br>(17.1, 20.0)	1539<br>(1418, 1694)	19.3<br>(17.8, 21.1)	4.2<br>(-3.5, 13.1)
**Libya**	594<br>(547, 666)	18.7<br>(17.3, 20.5)	1262<br>(1158, 1381)	18.3<br>(16.9, 19.8)	-1.9<br>(-10.9, 6.3)
**Morocco**	3507<br>(2819, 4298)	17.7<br>(14.1, 22.1)	10323<br>(8099, 12868)	28.5<br>(22.4, 35.1)	61.4<br>(31.5, 94.1)

**Palestine**	446<br>(351, 574)	34.8<br>(27.2, 45.7)	1161<br>(933, 1445)	30.2<br>(24.4, 37.7)	-13.2<br>(-23.5, -0.3)
**Oman**	367<br>(313, 437)	25.5<br>(22.2, 29.5)	1264<br>(1034, 1595)	27.6<br>(23.5, 33.3)	8.4<br>(-6.1, 31.8)
**Qatar**	178<br>(158, 210)	43.1<br>(38.9, 50.2)	1233<br>(1088, 1476)	40.1<br>(36.2, 46.0)	-6.9<br>(-20.1, 6.9)
**Saudi Arabia**	2899<br>(2601, 3294)	23.9<br>(21.6, 26.9)	9375<br>(8370, 11115)	25.4<br>(22.9, 29.1)	6.3<br>(-5.6, 21.7)
**Sudan**	1732<br>(1414, 2063)	11.9<br>(9.7, 14.2)	6979<br>(5563, 8695)	24.0<br>(19.1, 30.0)	101.0<br>(69.3, 137.3)
**Syria**	2572<br>(2033, 3235)	30.8<br>(24.3, 39.5)	4707<br>(3715, 5943)	28.8<br>(22.6, 36.0)	-6.7<br>(-16.1, 3.2)
**Tunisia**	1724<br>(1393, 2141)	25.5<br>(20.5, 31.8)	4128<br>(3224, 5282)	32.2<br>(25.4, 40.9)	26.3<br>(10.1, 47.2)
**Turkey**	16031<br>(14499, 18032)	33.5<br>(30.3, 37.5)	38881<br>(35173, 43130)	43.6<br>(39.6, 48.1)	30.1<br>(15.8, 44.6)
**United Arab Emirates**	511<br>(386, 669)	31.0<br>(24.4, 38.8)	3682<br>(2607, 5186)	28.7<br>(22.5, 37.6)	-7.5<br>(-19.6, 11.5)
**Yemen**	1005<br>(819, 1216)	11.4<br>(9.4, 13.7)	5065<br>(3969, 6373)	23.4<br>(18.4, 29.4)	104.5<br>(71.8, 143.0)
**South Asia**	109710<br>(101262, 118952)	12.5<br>(11.6, 13.5)	267099<br>(243347, 295122)	16.1<br>(14.7, 17.8)	29.0<br>(24.3, 34.0)
**Bangladesh**	8164<br>(7401, 9027)	10.6<br>(9.5, 11.9)	23839<br>(21203, 27231)	16.2<br>(14.3, 18.5)	52.6<br>(35, 71.3)
**Bhutan**	51<br>(46, 55)	12.6<br>(11.4, 14.0)	148<br>(133, 166)	16.9<br>(15.2, 18.8)	33.6<br>(24.5, 43.4)
**India**	89149<br>(82262, 96873)	12.6<br>(11.6, 13.6)	212451<br>(192979, 235104)	16.2<br>(14.7, 17.9)	28.6<br>(23.8, 33.5)
**Nepal**	1581<br>(1458, 1714)	11.0<br>(10.1, 12.0)	3725<br>(3410, 4179)	14.0<br>(12.8, 15.8)	27.6<br>(17.7, 41.4)
**Pakistan**	10766<br>(9802, 11837)	13.9<br>(12.6, 15.5)	26936<br>(24106, 29871)	15.9<br>(14.3, 17.8)	14.3<br>(6.9, 21.6)
**Southern sub-Saharan Africa**	6729<br>(6200, 7309)	16.9<br>(15.6, 18.4)	7549<br>(6917, 8191)	10.7<br>(9.9, 11.6)	-36.6<br>(-39.7, -33.1)
**Botswana**	121<br>(113, 130)	12.7<br>(11.9, 13.7)	199<br>(182, 220)	9.8<br>(9.0, 10.8)	-22.8<br>(-28.3, -15.5)
**Lesotho**	115<br>(107, 123)	8.4<br>(7.8, 9.0)	143<br>(133, 152)	8.7<br>(8.1, 9.3)	3.5<br>(-2.6, 9.5)
**Namibia**	121<br>(112, 132)	11.5<br>(10.6, 12.4)	207<br>(189, 226)	10.4<br>(9.6, 11.4)	-9.3<br>(-16.4, -1.9)
**South Africa**	5392<br>(4922, 5909)	18.5<br>(16.9, 20.3)	5954<br>(5404, 6519)	11.2<br>(10.2, 12.2)	-39.4<br>(-43.0, -35.5)
**Swaziland**	55<br>(50, 61)	10.3<br>(9.4, 11.4)	80<br>(73, 86)	9.0<br>(8.3, 9.8)	-12.8<br>(-20.5, -5.5)
**Zimbabwe**	925<br>(857, 995)	13.2<br>(12.2, 14.2)	966<br>(901, 1033)	8.8<br>(8.2, 9.5)	-33.1<br>(-36.5, -29.5)
**Western sub-Saharan Africa**	11958<br>(11218, 12750)	8.5<br>(8.0, 9.1)	34191<br>(31884, 36569)	11.2<br>(10.4, 12.0)	31.4<br>(24.8, 37.4)
**Benin**	261<br>(245, 280)	8.0<br>(7.5, 8.6)	900<br>(838, 966)	11.2<br>(10.4, 12.1)	40.3<br>(32.6, 47.8)
**Burkina Faso**	443<br>(417, 473)	6.5<br>(6.1, 7.0)	1500<br>(1395, 1611)	10.3<br>(9.6, 11.1)	58.1<br>(46.8, 69.2)
**Cameroon**	639<br>(590, 695)	8.6<br>(7.9, 9.4)	2058<br>(1900, 2234)	10.3<br>(9.6, 11.2)	20.5<br>(11.7, 28.7)
**Cape Verde**	64<br>(59, 68)	23.8<br>(22.1, 25.5)	74<br>(68, 82)	14.3<br>(13.1, 15.7)	-39.9<br>(-43.1, -34.7)
**Chad**	305<br>(285, 328)	7.2<br>(6.8, 7.8)	996<br>(923, 1071)	10.6<br>(9.8, 11.4)	46.3<br>(36.9, 56.3)
**Cote d'Ivoire**	623<br>(583, 668)	7.3<br>(6.8, 7.8)	1809<br>(1676, 1941)	9.8<br>(9.1, 10.6)	35.2<br>(26.7, 43.8)
**The Gambia**	76<br>(70, 81)	11.0<br>(10.2, 11.7)	185<br>(173, 199)	11.9<br>(11.1, 12.9)	8.6<br>(4.0, 13.2)

**Ghana**	862<br>(805, 925)	7.8<br>(7.2, 8.4)	2697<br>(2507, 2899)	11.0<br>(10.2, 11.9)	41.9<br>(32.9, 51.5)
**Guinea**	317<br>(296, 341)	6.9<br>(6.4, 7.4)	898<br>(834, 963)	10.8<br>(10.1, 11.7)	58.2<br>(46.3, 69.2)
**Guinea-Bissau**	45<br>(42, 47)	6.2<br>(5.8, 6.7)	126<br>(117, 136)	9.7<br>(9.0, 10.4)	55.1<br>(44.4, 65.6)
**Liberia**	105<br>(98, 113)	7.0<br>(6.5, 7.5)	380<br>(352, 410)	10.9<br>(10.1, 11.8)	56.1<br>(44.3, 66.4)
**Mali**	426<br>(398, 456)	6.8<br>(6.3, 7.3)	1454<br>(1354, 1557)	10.8<br>(10.1, 11.7)	60.1<br>(49.6, 71.4)
**Mauritania**	158<br>(145, 171)	10.4<br>(9.6, 11.4)	386<br>(351, 441)	13.0<br>(11.8, 14.7)	24.4<br>(12.5, 41.0)
**Niger**	378<br>(348, 418)	7.0<br>(6.4, 7.8)	1438<br>(1342, 1541)	11.1<br>(10.2, 11.9)	57.1<br>(41.6, 71.2)
**Nigeria**	6367<br>(5898, 6942)	9.4<br>(8.7, 10.3)	16806<br>(15595, 18045)	11.8<br>(10.9, 12.6)	24.5<br>(15.5, 33.0)
**Sao Tome and Principe**	11<br>(10, 12)	12.0<br>(11.1, 12.9)	21<br>(19, 22)	12.7<br>(11.8, 13.7)	6.2<br>(0.7, 11.4)
**Senegal**	466<br>(435, 500)	8.7<br>(8.1, 9.3)	1250<br>(1162, 1338)	11.4<br>(10.5, 12.2)	30.5<br>(23.4, 37.1)
**Sierra Leone**	225<br>(209, 241)	7.9<br>(7.4, 8.5)	607<br>(565, 651)	10.6<br>(9.9, 11.5)	34.3<br>(26.0, 42.5)
**Togo**	189<br>(176, 203)	7.6<br>(7.1, 8.2)	605<br>(559, 650)	10.4<br>(9.7, 11.2)	37.7<br>(29.0, 46.1)
**Eastern sub-Saharan Africa**	10526<br>(9868, 11247)	7.9<br>(7.4, 8.4)	27280<br>(25463, 29354)	9.9<br>(9.2, 10.6)	25.8<br>(21.7, 30.0)
**Burundi**	225<br>(204, 254)	5.4<br>(4.9, 6.2)	700<br>(648, 763)	9.1<br>(8.4, 9.9)	68.2<br>(46.9, 88.7)
**Comoros**	26<br>(24, 28)	7.6<br>(7.0, 8.4)	76<br>(69, 85)	12.1<br>(11.1, 13.6)	58.7<br>(41.7, 80.0)
**Djibouti**	35<br>(31, 41)	10.1<br>(9.2, 11.3)	116<br>(105, 132)	12.1<br>(11.0, 13.5)	19.2<br>(6.5, 33.7)
**Eritrea**	126<br>(116, 137)	6.0<br>(5.5, 6.6)	444<br>(412, 480)	10.2<br>(9.4, 11.0)	68.7<br>(53.9, 84.1)
**Ethiopia**	3115<br>(2848, 3432)	8.8<br>(8.1, 9.7)	6918<br>(6306, 7602)	9.7<br>(8.8, 10.6)	10.1<br>(4.4, 15.6)
**Kenya**	1816<br>(1670, 1982)	11.8<br>(10.8, 12.8)	3999<br>(3658, 4374)	11.0<br>(10.0, 12.0)	-6.8<br>(-9.0, -4.4)
**Madagascar**	652<br>(603, 703)	7.7<br>(7.0, 8.4)	2076<br>(1910, 2234)	11.0<br>(10.2, 11.9)	43.7<br>(31.1, 56.9)
**Malawi**	443<br>(409, 482)	6.3<br>(5.9, 6.9)	1056<br>(981, 1135)	8.9<br>(8.3, 9.6)	40.3<br>(27.9, 52.7)
**Mozambique**	651<br>(607, 698)	6.2<br>(5.7, 6.7)	1941<br>(1795, 2091)	9.8<br>(9.1, 10.5)	58.6<br>(46.9, 71.4)
**Rwanda**	317<br>(291, 344)	6.1<br>(5.5, 6.7)	919<br>(851, 984)	9.8<br>(9.0, 10.5)	60.7<br>(44.8, 77.8)
**Somalia**	303<br>(281, 328)	5.8<br>(5.3, 6.3)	1100<br>(1020, 1177)	9.6<br>(8.9, 10.3)	66.2<br>(51.2, 81.0)
**South Sudan**	315<br>(287, 350)	7.4<br>(6.8, 8.2)	705<br>(652, 761)	10.5<br>(9.7, 11.3)	41.1<br>(26.7, 54.7)
**Tanzania**	1406<br>(1314, 1500)	7.9<br>(7.3, 8.4)	3811<br>(3558, 4090)	10.0<br>(9.3, 10.7)	26.8<br>(20.3, 33.0)
**Uganda**	711<br>(665, 759)	5.9<br>(5.5, 6.3)	2357<br>(2178, 2572)	9.2<br>(8.5, 10.0)	56.5<br>(44.5, 70.1)
**Zambia**	382<br>(354, 417)	7.2<br>(6.6, 7.9)	1046<br>(962, 1159)	8.8<br>(8.1, 9.8)	23.1<br>(11.4, 35.6)
**Central sub-Saharan Africa**	3165<br>(2948, 3385)	7.7<br>(7.2, 8.2)	9006<br>(8400, 9629)	10.2<br>(9.5, 11.0)	33.2<br>(25.2, 40.8)
**Angola**	531<br>(475, 601)	7.1<br>(6.3, 8.2)	2063<br>(1889, 2276)	10.6<br>(9.7, 11.7)	49.6<br>(30.1, 69.8)
**Central African Republic**	120<br>(108, 136)	5.6<br>(5.0, 6.3)	300<br>(278, 324)	8.4<br>(7.7, 9.0)	49.6<br>(31.4, 68.4)

**Congo**	133<br>(122, 144)	7.2<br>(6.6, 7.9)	390<br>(360, 424)	9.6<br>(8.9, 10.4)	33.5<br>(22.1, 44.7)
**DR Congo**	2254<br>(2088, 2434)	7.9<br>(7.3, 8.5)	5993<br>(5567, 6445)	10.2<br>(9.5, 11.0)	30.0<br>(21.1, 38.5)
**Equatorial Guinea**	18<br>(17, 20)	5.7<br>(5.3, 6.3)	99<br>(89, 113)	10.7<br>(9.6, 12.2)	86.7<br>(67.5, 112.9)
**Gabon**	109<br>(101, 117)	13.3<br>(12.3, 14.3)	159<br>(148, 172)	10.7<br>(10.0, 11.6)	-19.0<br>(-23.8, -14.8)

Appendix table 3: Deaths due to IBD in 1990 and 2017 for both sexes and percentage change in age-standardised rates by location

	1990		2017		Percentage change in age-standardized rates between 1990 and 2017
	Counts (95% UI)	Rate (95% UI)	Counts (95% UI)	Rate (95% UI)	
**Global**	23126 (20184, 27466)	0.6 (0.5, 0.7)	38629 (31595, 41157)	0.5 (0.4, 0.5)	-16.4 (-36.0, -4.7)
**High-income North America**	2215 (2061, 2920)	0.6 (0.6, 0.8)	5254 (3471, 5785)	0.8 (0.6, 0.9)	36.9 (-22.3, 57.1)
**Canada**	242 (204, 300)	0.7 (0.6, 0.9)	375 (328, 483)	0.5 (0.5, 0.7)	-29.4 (-38.5, -5.3)
**Greenland**	0 (0, 0)	0.3 (0.3, 0.4)	0 (0, 0)	0.5 (0.3, 0.6)	51.7 (-10.9, 113.3)
**USA**	1973 (1825, 2630)	0.6 (0.6, 0.8)	4878 (3064, 5413)	0.9 (0.6, 1.0)	46.0 (-21.1, 69.1)
**Australasia**	114 (102, 153)	0.5 (0.4, 0.7)	327 (194, 391)	0.6 (0.4, 0.7)	19.2 (-39.3, 46.3)
**Australia**	95 (85, 133)	0.5 (0.4, 0.7)	301 (176, 363)	0.6 (0.4, 0.8)	28.5 (-38.5, 62.1)
**New Zealand**	19 (11, 22)	0.5 (0.3, 0.6)	26 (18, 30)	0.3 (0.2, 0.4)	-35.1 (-49.0, -1.6)
**High-income Asia-Pacific**	681 (418, 769)	0.4 (0.2, 0.4)	772 (645, 1195)	0.2 (0.1, 0.2)	-59.4 (-70.0, -3.1)
**Brunei**	1 (1, 2)	1.4 (1.0, 1.7)	3 (2, 3)	1.0 (0.9, 1.3)	-26.3 (-40.9, -0.4)
**Japan**	357 (288, 376)	0.2 (0.2, 0.2)	491 (406, 815)	0.1 (0.1, 0.2)	-47.2 (-56.9, -5.5)
**Singapore**	4 (3, 4)	0.2 (0.1, 0.2)	6 (4, 9)	0.1 (0.1, 0.1)	-53.0 (-63.8, -13.1)
**South Korea**	319 (122, 398)	1.6 (0.5, 2.1)	273 (222, 372)	0.4 (0.3, 0.5)	-77.8 (-84.6, -8.7)
**Western Europe**	4263 (3943, 5665)	0.7 (0.7, 1.0)	10356 (5627, 11839)	1.0 (0.5, 1.1)	33.6 (-37.5, 59.0)
**Andorra**	0 (0, 1)	0.7 (0.5, 1.0)	1 (1, 1)	0.7 (0.4, 1.0)	-3.0 (-32.4, 37.4)
**Austria**	100 (89, 136)	0.9 (0.8, 1.1)	101 (85, 156)	0.5 (0.4, 0.8)	-38.2 (-48.5, -9.1)
**Belgium**	97 (87, 123)	0.6 (0.6, 0.8)	228 (119, 275)	0.8 (0.5, 1.0)	32.8 (-35.6, 66.7)
**Cyprus**	10 (5, 14)	1.4 (0.6, 1.9)	11 (6, 13)	0.6 (0.3, 0.7)	-59.8 (-70.0, -35.2)
**Denmark**	42 (33, 51)	0.5 (0.4, 0.6)	70 (50, 81)	0.6 (0.4, 0.7)	17.0 (-24.5, 42.6)
**Finland**	29 (15, 37)	0.4 (0.2, 0.5)	34 (24, 40)	0.3 (0.2, 0.3)	-36.1 (-50.4, -1.3)
**France**	835 (633, 936)	0.9 (0.7, 1.0	1846 (688, 2305)	1.0 (0.4, 1.3)	12.8 (-49.0, 37.2)
**Germany**	1121 (901, 1996)	0.9 (0.7, 1.5)	3557 (1949, 4312)	1.6 (0.9, 2.0)	89.3 (-33.8, 165.4)
**Greece**	30 (22, 34)	0.2 (0.2, 0.2)	57 (39, 67)	0.2 (0.1, 0.2)	-4.6 (-18.7, 15.2)
**Iceland**	2 (1, 2)	0.5 (0.5, 0.8)	3 (2, 4)	0.6 (0.4, 0.6)	1.0 (-42.9, 28.8)
**Ireland**	31 (24, 35)	0.8 (0.6, 0.9)	46 (28, 55)	0.6 (0.4, 0.7)	-22.0 (-50.6, -4.8)
**Israel**	23 (18, 27)	0.5 (0.4, 0.6)	50 (36, 58)	0.4 (0.3, 0.5)	-15.5 (-36.1, 0.2)
**Italy**	338 (289, 547)	0.4 (0.3, 0.6)	1353 (605, 1643)	0.8 (0.3, 1.0)	99.0 (-39.4, 170.6)

**Luxembourg**	4<br>(4, 5)	0.8<br>(0.7, 1.0)	11<br>(5, 15)	1.1<br>(0.5, 1.4)	39.7<br>(-42.7, 84.9)
**Malta**	2<br>(2, 2)	0.5<br>(0.4, 0.6)	6<br>(3, 7)	0.6<br>(0.4, 0.8)	36.5<br>(-30.5, 72.0)
**Netherlands**	255<br>(229, 309)	1.3<br>(1.1, 1.5)	627<br>(329, 745)	1.7<br>(0.9, 2)	34.2<br>(-33, 62.0)
**Norway**	29<br>(25, 47)	0.4<br>(0.4, 0.7)	41<br>(36, 52)	0.4<br>(0.4, 0.5)	-3.3<br>(-41.4, 13.6)
**Portugal**	75<br>(66, 83)	0.6<br>(0.5, 0.7)	252<br>(95, 317)	0.9<br>(0.3, 1.1)	44.3<br>(-42.5, 84.3)
**Spain**	235<br>(202, 266)	0.4<br>(0.4, 0.5)	362<br>(316, 471)	0.3<br>(0.3, 0.4)	-23.6<br>(-39.3, -1.3)
**Sweden**	98<br>(67, 109)	0.6<br>(0.4, 0.7)	83<br>(73, 104)	0.3<br>(0.3, 0.5)	-43.4<br>(-52.2, -3.5)
**Switzerland**	63<br>(34, 78)	0.6<br>(0.3, 0.7)	104<br>(68, 124)	0.5<br>(0.3, 0.6)	-8.0<br>(-26.3, 31.8)
**United Kingdom**	838<br>(780, 1062)	0.9<br>(0.8, 1.1)	1501<br>(995, 1649)	1.1<br>(0.7, 1.2)	19.9<br>(-29.1, 35.7)
**Southern Latin America**	165<br>(142, 197)	0.4<br>(0.3, 0.4)	231<br>(202, 268)	0.3<br>(0.2, 0.3)	-21.2<br>(-35.3, -2.6)
**Argentina**	109<br>(95, 129)	0.3<br>(0.3, 0.4)	145<br>(125, 170)	0.3<br>(0.2, 0.3)	-20.0<br>(-36.1, -2.0)
**Chile**	45<br>(33, 51)	0.4<br>(0.3, 0.5)	64<br>(55, 80)	0.3<br>(0.2, 0.4)	-36.0<br>(-48.1, -2.2)
**Uruguay**	11<br>(9, 19)	0.3<br>(0.2, 0.5)	22<br>(15, 25)	0.4<br>(0.3, 0.5)	38.7<br>(-32.8, 86.9)
**Eastern Europe**	1476<br>(1107, 1655)	0.5<br>(0.4, 0.6)	1563<br>(1199, 1679)	0.5<br>(0.4, 0.5)	-10.8<br>(-18.3, 2.0)
**Belarus**	58<br>(46, 66)	0.5<br>(0.4, 0.5)	65<br>(46, 78)	0.4<br>(0.3, 0.5)	-5.0<br>(-27.6, 13.8)
**Estonia**	12<br>(9, 15)	0.6<br>(0.4, 0.8)	9<br>(7, 11)	0.4<br>(0.3, 0.4)	-40.1<br>(-55.6, -8.1)
**Latvia**	13<br>(11, 17)	0.4<br>(0.3, 0.5)	13<br>(10, 16)	0.4<br>(0.3, 0.4)	2.9<br>(-32.0, 30.2)
**Lithuania**	16<br>(12, 18)	0.3<br>(0.3, 0.4)	19<br>(13, 22)	0.3<br>(0.2, 0.4)	0.5<br>(-26.4, 19.1)
**Moldova**	18<br>(15, 20)	0.4<br>(0.3, 0.5)	17<br>(14, 20)	0.3<br>(0.3, 0.4)	-17.8<br>(-29.5, -6.8)
**Russia**	1127<br>(797, 1282)	0.6<br>(0.5, 0.7)	1162<br>(845, 1258)	0.5<br>(0.4, 0.6)	-18.2<br>(-25.8, -7.4)
**Ukraine**	233<br>(194, 286)	0.3<br>(0.3, 0.4)	276<br>(246, 329)	0.4<br>(0.4, 0.5)	22.2<br>(-1.5, 51.8)
**Central Europe**	631<br>(572, 818)	0.5<br>(0.4, 0.6)	917<br>(658, 1004)	0.5<br>(0.3, 0.5)	-2.8<br>(-41.4, 13.8)
**Albania**	44<br>(14, 55)	1.4<br>(0.6, 1.7)	11<br>(8, 21)	0.3<br>(0.3, 0.6)	-76.2<br>(-83.6, -4.9)
**Bosnia and Herzegovina**	13<br>(7, 16)	0.3<br>(0.2, 0.4)	15<br>(9, 19)	0.3<br>(0.2, 0.3)	-18.3<br>(-33.9, 3.9)
**Bulgaria**	21<br>(18, 31)	0.2<br>(0.2, 0.3)	34<br>(24, 39)	0.3<br>(0.2, 0.3)	27.3<br>(-31.3, 60.6)
**Croatia**	16<br>(14, 27)	0.3<br>(0.2, 0.4)	31<br>(21, 36)	0.4<br>(0.2, 0.4)	37.6<br>(-37.2, 80.9)
**Czech Republic**	69<br>(60, 96)	0.5<br>(0.5, 0.7)	119<br>(65, 142)	0.6<br>(0.3, 0.7)	13.7<br>(-51.1, 44.2)
**Hungary**	87<br>(76, 127)	0.6<br>(0.6, 0.9)	95<br>(80, 113)	0.5<br>(0.4, 0.6)	-17.3<br>(-47.8, -1.4)
**Macedonia**	3<br>(3, 4)	0.2<br>(0.2, 0.2)	5<br>(3, 6)	0.2<br>(0.1, 0.2)	-17.8<br>(-38.6, 4.3)
**Montenegro**	2<br>(1, 2)	0.3<br>(0.2, 0.4)	3<br>(2, 3)	0.3<br>(0.2, 0.3)	-9.6<br>(-26.1, 9.5)
**Poland**	217<br>(172, 300)	0.5<br>(0.4, 0.7)	328<br>(214, 379)	0.5<br>(0.3, 0.5)	-4.5<br>(-48.8, 25.5)

**Romania**	60<br>(51, 81)	0.2<br>(0.2, 0.3)	72<br>(61, 86)	0.2<br>(0.2, 0.3)	-9.9<br>(-37.4, 13.4)
**Serbia**	56<br>(42, 75)	0.5<br>(0.4, 0.7)	126<br>(72, 147)	0.8<br>(0.5, 1.0)	55.5<br>(-29.6, 105.4)
**Slovakia**	32<br>(26, 55)	0.6<br>(0.5, 1.0)	63<br>(46, 73)	0.8<br>(0.6, 0.9)	34.9<br>(-34.8, 83.6)
**Slovenia**	10<br>(8, 11)	0.4<br>(0.3, 0.5)	16<br>(10, 19)	0.4<br>(0.2, 0.4)	-17.3<br>(-48.2, -1.6)
**Central Asia**	222<br>(177, 268)	0.4<br>(0.3, 0.5)	235<br>(183, 265)	0.3<br>(0.2, 0.3)	-25.6<br>(-38.3, -15.9)
**Armenia**	7<br>(5, 8)	0.2<br>(0.2, 0.3)	10<br>(6, 11)	0.2<br>(0.2, 0.3)	2.9<br>(-21.5, 23.4)
**Azerbaijan**	9<br>(8, 12)	0.2<br>(0.1, 0.2)	14<br>(11, 18)	0.2<br>(0.1, 0.2)	-4.8<br>(-37.2, 34.5)
**Georgia**	14<br>(8, 16)	0.2<br>(0.1, 0.3)	14<br>(7, 17)	0.2<br>(0.1, 0.3)	6.5<br>(-6.7, 21.5)
**Kazakhstan**	79<br>(62, 93)	0.6<br>(0.4, 0.7)	82<br>(52, 99)	0.5<br>(0.3, 0.6)	-20.2<br>(-40.8, -2.8)
**Kyrgyzstan**	19<br>(11, 23)	0.5<br>(0.3, 0.6)	12<br>(9, 15)	0.2<br>(0.2, 0.3)	-55.1<br>(-68.5, -18.8)
**Mongolia**	11<br>(5, 16)	0.7<br>(0.4, 1.0)	11<br>(7, 16)	0.4<br>(0.3, 0.6)	-41.7<br>(-59.8, -18.0)
**Tajikistan**	21<br>(13, 39)	0.4<br>(0.3, 0.6)	22<br>(17, 27)	0.3<br>(0.2, 0.4)	-25.0<br>(-53.3, 5.0)
**Turkmenistan**	10<br>(7, 14)	0.4<br>(0.3, 0.5)	12<br>(10, 15)	0.3<br>(0.2, 0.3)	-24.9<br>(-37.5, -7.6)
**Uzbekistan**	52<br>(35, 64)	0.3<br>(0.2, 0.3)	59<br>(48, 70)	0.2<br>(0.2, 0.3)	-27.2<br>(-41.3, -3.4)
**Central Latin America**	297<br>(276, 345)	0.3<br>(0.3, 0.4)	753<br>(544, 823)	0.3<br>(0.2, 0.4)	5.0<br>(-25.5, 19.9)
**Colombia**	57<br>(51, 68)	0.3<br>(0.3, 0.3)	147<br>(89, 176)	0.3<br>(0.2, 0.3)	-3.2<br>(-44.2, 18.5)
**Costa Rica**	3<br>(2, 4)	0.2<br>(0.1, 0.2)	9<br>(6, 10)	0.2<br>(0.1, 0.2)	12.8<br>(-19.2, 40.7)
**El Salvador**	14<br>(7, 17)	0.4<br>(0.2, 0.5)	9<br>(7, 13)	0.2<br>(0.1, 0.2)	-58.9<br>(-70.5, -5.2)
**Guatemala**	11<br>(7, 22)	0.2<br>(0.2, 0.4)	32<br>(19, 38)	0.3<br>(0.2, 0.3)	28.7<br>(-50.4, 92.8)
**Honduras**	16<br>(11, 21)	0.6<br>(0.4, 0.8)	39<br>(26, 56)	0.6<br>(0.4, 0.9)	4.1<br>(-26.7, 45.3)
**Mexico**	157<br>(147, 194)	0.3<br>(0.3, 0.4)	451<br>(319, 497)	0.4<br>(0.3, 0.4)	21.5<br>(-15.6, 39.6)
**Nicaragua**	3<br>(3, 4)	0.2<br>(0.1, 0.2)	6<br>(4, 7)	0.1<br>(0.1, 0.1)	-28.4<br>(-46.4, -9.7)
**Panama**	7<br>(6, 12)	0.4<br>(0.3, 0.7)	24<br>(17, 28)	0.6<br>(0.4, 0.7)	39.5<br>(-34.7, 97.2)
**Venezuela**	29<br>(17, 33)	0.2<br>(0.2, 0.3)	38<br>(32, 46)	0.1<br>(0.1, 0.2)	-45.6<br>(-58.2, -5.2)
**Andean Latin America**	176<br>(116, 301)	0.5<br>(0.4, 0.8)	144<br>(123, 164)	0.3<br>(0.2, 0.3)	-52.9<br>(-70.3, -34.2)
**Bolivia**	27<br>(11, 64)	0.5<br>(0.3, 1.0)	35<br>(25, 45)	0.4<br>(0.3, 0.5)	-26.4<br>(-63.2, 36.3)
**Ecuador**	20<br>(15, 25)	0.3<br>(0.2, 0.3)	34<br>(24, 40)	0.2<br>(0.2, 0.3)	-10.9<br>(-43.1, 14.2)
**Peru**	130<br>(82, 227)	0.7<br>(0.5, 1.0)	76<br>(62, 96)	0.2<br>(0.2, 0.3)	-65.6<br>(-79.4, -47.1)
**Caribbean**	150<br>(118, 212)	0.5<br>(0.4, 0.7)	187<br>(159, 226)	0.4<br>(0.3, 0.5)	-29.8<br>(-42.9, -13.2)
**Antigua and Barbuda**	0<br>(0, 0)	0.8<br>(0.5, 0.9)	0<br>(0, 1)	0.4<br>(0.4, 0.6)	-43.8<br>(-55.7, -7.9)
**The Bahamas**	1<br>(1, 1)	0.7<br>(0.6, 0.9)	2<br>(2, 2)	0.5<br>(0.5, 0.6)	-23.5<br>(-39.1, -3.6)

**Barbados**	1<br>(1, 2)	0.5<br>(0.4, 0.6)	2<br>(1, 2)	0.4<br>(0.3, 0.4)	-21.9<br>(-37.0, -2.7)
**Belize**	1<br>(1, 1)	0.8<br>(0.6, 0.9)	2<br>(1, 2)	0.6<br>(0.5, 0.8)	-25.5<br>(-46.9, 41.1)
**Bermuda**	0<br>(0, 1)	0.8<br>(0.5, 1.0)	0<br>(0, 1)	0.4<br>(0.3, 0.5)	-53.3<br>(-65.4, -12.7)
**Cuba**	34<br>(18, 42)	0.3<br>(0.2, 0.4)	35<br>(27, 40)	0.2<br>(0.2, 0.2)	-41.1<br>(-54.0, -5.4)
**Dominica**	1<br>(0, 1)	0.7<br>(0.6, 0.8)	1<br>(0, 1)	0.7<br>(0.6, 0.8)	-9.2<br>(-28.8, 18.7)
**Dominican Republic**	12<br>(8, 15)	0.3<br>(0.2, 0.3)	19<br>(14, 30)	0.2<br>(0.1, 0.3)	-17.9<br>(-46.9, 46.0)
**Grenada**	1<br>(1, 1)	1.3<br>(1.1, 1.7)	1<br>(1, 2)	0.9<br>(0.8, 1.1)	-34.0<br>(-48.8, -8.6)
**Guyana**	4<br>(4, 5)	0.9<br>(0.8, 1.1)	5<br>(4, 6)	0.8<br>(0.7, 0.9)	-18.3<br>(-31.9, -0.5)
**Haiti**	41<br>(15, 94)	1.0<br>(0.5, 1.8)	68<br>(42, 95)	1.0<br>(0.6, 1.4)	-3.3<br>(-43.5, 72.5)
**Jamaica**	8<br>(7, 11)	0.4<br>(0.3, 0.6)	13<br>(11, 17)	0.4<br>(0.4, 0.6)	9.8<br>(-16.5, 43.2)
**Puerto Rico**	32<br>(18, 37)	0.9<br>(0.5, 1.0)	21<br>(17, 32)	0.3<br>(0.3, 0.5)	-63.9<br>(-73.7, -10.4)
**Saint Lucia**	1<br>(1, 1)	0.8<br>(0.6, 1.0)	1<br>(1, 1)	0.5<br>(0.5, 0.7)	-34.0<br>(-49.2, 7.7)
**Saint Vincent and the Grenadines**	0<br>(0, 1)	0.5<br>(0.3, 1.4)	1<br>(1, 1)	1.0<br>(0.8, 1.1)	75.6<br>(-30.9, 187.8)
**Suriname**	2<br>(2, 3)	0.9<br>(0.7, 1.0)	4<br>(3, 5)	0.7<br>(0.6, 0.9)	-22.6<br>(-38.4, 13.8)
**Trinidad and Tobago**	3<br>(2, 4)	0.4<br>(0.2, 0.4)	4<br>(3, 6)	0.3<br>(0.2, 0.3)	-27.1<br>(-45.0, -2.6)
**Virgin Islands**	1<br>(0, 1)	0.7<br>(0.5, 0.8)	1<br>(1, 1)	0.4<br>(0.3, 0.5)	-37.1<br>(-51.6, -7.5)
**Tropical Latin America**	450<br>(376, 480)	0.5<br>(0.4, 0.5)	1014<br>(765, 1099)	0.4<br>(0.3, 0.5)	-3.9<br>(-23.4, 3.9)
**Brazil**	446<br>(371, 475)	0.5<br>(0.4, 0.5)	1003<br>(753, 1089)	0.4<br>(0.3, 0.5)	-4.2<br>(-23.6, 3.6)
**Paraguay**	5<br>(4, 6)	0.2<br>(0.2, 0.3)	12<br>(9, 15)	0.2<br>(0.2, 0.3)	14.8<br>(-18.7, 50.5)
**East Asia**	5977<br>(3954, 6899)	0.8<br>(0.6, 1.0)	5612<br>(5225, 6807)	0.3<br>(0.3, 0.4)	-59.6<br>(-66.3, -27.3)
**China**	5701<br>(3725, 6612)	0.8<br>(0.6, 1.0)	5199<br>(4842, 6383)	0.3<br>(0.3, 0.4)	-60.7<br>(-67.5, -28.0)
**North Korea**	80<br>(55, 103)	0.6<br>(0.4, 0.8)	126<br>(92, 165)	0.5<br>(0.4, 0.7)	-20.4<br>(-42.1, 7.1)
**Taiwan (Province of China)**	95<br>(61, 108)	0.8<br>(0.5, 1.0)	197<br>(152, 222)	0.5<br>(0.4, 0.6)	-39.0<br>(-47.6, -8.8)
**Southeast Asia**	1419<br>(1036, 1709)	0.6<br>(0.4, 0.7)	1825<br>(1606, 2160)	0.3<br>(0.3, 0.4)	-40.0<br>(-48.8, -14.0)
**Cambodia**	28<br>(16, 49)	0.6<br>(0.4, 0.9)	45<br>(36, 56)	0.5<br>(0.4, 0.6)	-28.4<br>(-51.6, 10.2)
**Indonesia**	706<br>(491, 896)	0.8<br>(0.5, 1.1)	985<br>(792, 1333)	0.6<br>(0.4, 0.8)	-30.7<br>(-44.1, -7.7)
**Laos**	11<br>(5, 18)	0.5<br>(0.3, 0.7)	18<br>(12, 28)	0.5<br>(0.3, 0.7)	-7.9<br>(-46.7, 38.3)
**Malaysia**	23<br>(19, 28)	0.3<br>(0.2, 0.3)	53<br>(41, 68)	0.2<br>(0.2, 0.3)	-11.9<br>(-31.5, 22.0)
**Maldives**	0<br>(0, 1)	0.7<br>(0.4, 1.0)	1<br>(1, 1)	0.3<br>(0.2, 0.4)	-55.1<br>(-72.8, -16.5)
**Mauritius**	1<br>(1, 2)	0.2<br>(0.1, 0.2)	3<br>(2, 3)	0.2<br>(0.1, 0.2)	0.7<br>(-34.8, 27.1)
**Myanmar**	87<br>(42, 171)	0.4<br>(0.2, 0.7)	112<br>(84, 151)	0.3<br>(0.2, 0.4)	-26.8<br>(-54.9, 22.4)

**Philippines**	234<br>(136, 311)	0.8<br>(0.4, 0.9)	189<br>(141, 345)	0.3<br>(0.2, 0.5)	-61.3<br>(-73.9, 10.1)
**Sri Lanka**	18<br>(14, 23)	0.2<br>(0.1, 0.2)	21<br>(16, 27)	0.1<br>(0.1, 0.1)	-44.5<br>(-60.9, -20.7)
**Seychelles**	0<br>(0, 0)	0.4<br>(0.3, 0.4)	0<br>(0, 0)	0.3<br>(0.2, 0.3)	-28.8<br>(-41.4, -11.9)
**Thailand**	97<br>(72, 120)	0.2<br>(0.2, 0.3)	170<br>(139, 196)	0.2<br>(0.2, 0.2)	-26.5<br>(-46.0, 1.4)
**East Timor**	2<br>(1, 3)	0.5<br>(0.3, 0.7)	5<br>(3, 8)	0.6<br>(0.3, 1.0)	17.4<br>(-28.4, 74.9)
**Vietnam**	209<br>(124, 290)	0.5<br>(0.3, 0.7)	221<br>(179, 267)	0.3<br>(0.2, 0.3)	-50.6<br>(-69.3, -20.0)
**Oceania**	19<br>(12, 28)	0.6<br>(0.4, 0.8)	28<br>(21, 39)	0.4<br>(0.3, 0.5)	-32.5<br>(-44.5, -13.4)
**American Samoa**	0<br>(0, 1)	1.9<br>(1.0, 2.5)	0<br>(0, 0)	0.5<br>(0.4, 0.8)	-71.8<br>(-80.5, -27.2)
**Federated States of Micronesia**	1<br>(0, 1)	1.2<br>(0.7, 1.7)	0<br>(0, 1)	0.6<br>(0.4, 0.8)	-45.8<br>(-61.3, -22.2)
**Fiji**	1<br>(1, 2)	0.3<br>(0.3, 0.4)	2<br>(1, 2)	0.2<br>(0.2, 0.3)	-26.6<br>(-44.9, -4.8)
**Guam**	0<br>(0, 0)	0.3<br>(0.2, 0.4)	0<br>(0, 0)	0.2<br>(0.2, 0.3)	-39.5<br>(-57.8, -10.4)
**Kiribati**	0<br>(0, 1)	1.0<br>(0.5, 1.6)	1<br>(0, 1)	0.8<br>(0.4, 1.1)	-22.7<br>(-43.6, 6.3)
**Marshall Islands**	0<br>(0, 0)	1.0<br>(0.6, 1.5)	0<br>(0, 0)	0.7<br>(0.5, 1.0)	-28.8<br>(-47.4, 0.4)
**Northern Mariana Islands**	0<br>(0, 0)	1.2<br>(0.7, 1.6)	0<br>(0, 0)	0.4<br>(0.3, 0.6)	-64.1<br>(-74.6, -20.4)
**Papua New Guinea**	11<br>(6, 19)	0.5<br>(0.3, 0.7)	17<br>(11, 28)	0.4<br>(0.3, 0.5)	-28.5<br>(-46.7, -0.7)
**Samoa**	1<br>(0, 1)	0.9<br>(0.4, 1.3)	1<br>(0, 1)	0.5<br>(0.3, 0.8)	-35.8<br>(-52.1, -7.5)
**Solomon Islands**	1<br>(0, 1)	0.6<br>(0.4, 0.9)	1<br>(1, 2)	0.4<br>(0.3, 0.5)	-37.5<br>(-55.1, -11.3)
**Tonga**	1<br>(0, 1)	1.1<br>(0.7, 1.6)	1<br>(0, 1)	0.8<br>(0.6, 1.1)	-30.3<br>(-47.2, -3.7)
**Vanuatu**	2<br>(1, 3)	2.1<br>(1.0, 3.5)	3<br>(1, 6)	1.8<br>(0.8, 3.2)	-15.1<br>(-48.7, 31.0)
**North Africa and Middle East**	524<br>(346, 777)	0.3<br>(0.2, 0.4)	872<br>(781, 999)	0.2<br>(0.2, 0.2)	-25.1<br>(-41.5, 15.0)
**Afghanistan**	20<br>(8, 39)	0.3<br>(0.1, 0.5)	38<br>(19, 65)	0.3<br>(0.2, 0.5)	9.7<br>(-25.2, 81.9)
**Algeria**	23<br>(14, 42)	0.2<br>(0.1, 0.3)	44<br>(26, 68)	0.1<br>(0.1, 0.2)	-26.5<br>(-44.6, -0.2)
**Bahrain**	1<br>(0, 1)	0.2<br>(0.2, 0.3)	1<br>(1, 1)	0.2<br>(0.1, 0.2)	-36.8<br>(-54.2, -13.9)
**Egypt**	46<br>(31, 72)	0.2<br>(0.1, 0.2)	84<br>(47, 142)	0.2<br>(0.1, 0.3)	-6.7<br>(-44.0, 57.4)
**Iran**	40<br>(32, 50)	0.2<br>(0.1, 0.2)	126<br>(65, 142)	0.2<br>(0.1, 0.2)	23.7<br>(-20.1, 59.2)
**Iraq**	42<br>(25, 67)	0.4<br>(0.3, 0.6)	59<br>(48, 72)	0.2<br>(0.2, 0.2)	-50.0<br>(-69.2, -17.7)
**Jordan**	5<br>(4, 9)	0.3<br>(0.2, 0.5)	13<br>(9, 16)	0.2<br>(0.2, 0.3)	-31.5<br>(-64.6, 15.6)
**Kuwait**	1<br>(1, 2)	0.2<br>(0.1, 0.2)	3<br>(2, 4)	0.1<br>(0.1, 0.2)	-22.8<br>(-48.5, 0.9)
**Lebanon**	3<br>(2, 6)	0.1<br>(0.1, 0.3)	7<br>(5, 11)	0.1<br>(0.1, 0.2)	-12.9<br>(-44.2, 110.5)
**Libya**	4<br>(2, 7)	0.2<br>(0.1, 0.3)	7<br>(4, 10)	0.2<br>(0.1, 0.2)	-17.7<br>(-41.6, 23.2)
**Morocco**	28<br>(19, 45)	0.2<br>(0.1, 0.3)	52<br>(40, 76)	0.2<br>(0.1, 0.3)	-10.3<br>(-32.7, 27.9)

**Palestine**	3 (2, 4)	0.3 (0.2, 0.4)	4 (3, 6)	0.2 (0.1, 0.2)	-37.7 (-62.7, 6.1)
**Oman**	1 (1, 2)	0.2 (0.1, 0.3)	2 (2, 3)	0.1 (0.1, 0.2)	-36.2 (-59.3, 5.6)
**Qatar**	1 (1, 1)	0.6 (0.4, 0.8)	3 (1, 4)	0.3 (0.2, 0.4)	-57.1 (-70.7, -35.9)
**Saudi Arabia**	11 (7, 15)	0.2 (0.1, 0.2)	17 (10, 26)	0.1 (0.1, 0.2)	-37.8 (-57.3, 3.8)
**Sudan**	17 (2, 37)	0.2 (0, 0.3)	31 (12, 56)	0.2 (0.1, 0.3)	0.8 (-40.0, 182.4)
**Syria**	22 (16, 33)	0.4 (0.3, 0.6)	35 (24, 44)	0.3 (0.2, 0.4)	-30.6 (-53.0, 5.5)
**Tunisia**	8 (5, 13)	0.2 (0.1, 0.3)	16 (11, 23)	0.1 (0.1, 0.2)	-17.2 (-41.0, 24.1)
**Turkey**	235 (126, 390)	0.6 (0.3, 0.9)	293 (252, 381)	0.3 (0.3, 0.4)	-40.6 (-60.3, 8.8)
**United Arab Emirates**	1 (0, 2)	0.2 (0.1, 0.3)	7 (4, 11)	0.1 (0.1, 0.2)	-11.1 (-47.0, 108.5)
**Yemen**	10 (4, 20)	0.2 (0.1, 0.3)	30 (19, 44)	0.2 (0.2, 0.3)	16.6 (-25.5, 102.5)
**South Asia**	2869 (1889, 4316)	0.5 (0.4, 0.6)	5737 (4634, 6674)	0.5 (0.4, 0.6)	-4.8 (-20.8, 17.8)
**Bangladesh**	280 (171, 468)	0.5 (0.3, 0.6)	501 (406, 607)	0.4 (0.3, 0.5)	-11.0 (-35.5, 36.9)
**Bhutan**	2 (1, 2)	0.6 (0.4, 0.9)	3 (2, 5)	0.5 (0.3, 0.8)	-18.3 (-53.2, 30.6)
**India**	2097 (1324, 3433)	0.4 (0.3, 0.6)	4331 (3392, 5479)	0.4 (0.3, 0.6)	-1.6 (-20.5, 28.4)
**Nepal**	53 (33, 78)	0.5 (0.4, 0.7)	96 (69, 127)	0.5 (0.4, 0.6)	-9.9 (-41.0, 26.1)
**Pakistan**	437 (284, 641)	0.8 (0.4, 1.2)	807 (480, 1271)	0.8 (0.4, 1.3)	7.7 (-19.0, 42.9)
**Southern sub-Saharan Africa**	167 (132, 209)	0.5 (0.4, 0.7)	250 (199, 293)	0.5 (0.4, 0.5)	-14.6 (-30.5, 4.8)
**Botswana**	4 (3, 6)	0.6 (0.5, 0.9)	6 (4, 9)	0.5 (0.3, 0.6)	-29.7 (-45.7, -5.7)
**Lesotho**	6 (4, 9)	0.6 (0.4, 0.9)	7 (5, 10)	0.6 (0.4, 0.9)	-3.1 (-34.5, 37.2)
**Namibia**	6 (4, 8)	0.7 (0.5, 1.0)	10 (5, 15)	0.6 (0.4, 0.9)	-12.8 (-43.1, 26.3)
**South Africa**	126 (94, 165)	0.5 (0.4, 0.7)	187 (135, 226)	0.4 (0.3, 0.5)	-18.5 (-32.3, 1.7)
**Swaziland**	2 (1, 3)	0.5 (0.3, 0.7)	3 (2, 4)	0.5 (0.3, 0.6)	-10.0 (-33.1, 34.5)
**Zimbabwe**	22 (16, 30)	0.4 (0.3, 0.6)	37 (20, 65)	0.5 (0.3, 0.9)	13.8 (-41.9, 73.8)
**Western sub-Saharan Africa**	593 (255, 1033)	0.5 (0.3, 0.8)	1089 (740, 1448)	0.5 (0.4, 0.8)	-0.6 (-41.0, 64.3)
**Benin**	14 (5, 28)	0.5 (0.2, 0.8)	25 (17, 34)	0.5 (0.3, 0.7)	-9.1 (-48.4, 98.3)
**Burkina Faso**	30 (15, 59)	0.6 (0.3, 0.8)	53 (20, 98)	0.5 (0.2, 0.9)	-6.8 (-51.2, 76.6)
**Cameroon**	29 (12, 50)	0.5 (0.3, 0.9)	59 (26, 93)	0.5 (0.2, 0.8)	-12.4 (-45.5, 54.0)
**Cape Verde**	1 (1, 1)	0.3 (0.2, 0.4)	1 (1, 1)	0.2 (0.2, 0.3)	-31.4 (-46.8, 13.1)
**Chad**	23 (11, 40)	0.7 (0.4, 1.0)	37 (23, 57)	0.6 (0.4, 0.7)	-15.2 (-41.3, 27.6)
**Cote d'Ivoire**	25 (11, 51)	0.5 (0.2, 0.8)	58 (38, 78)	0.5 (0.4, 0.7)	5.1 (-36.5, 101.1)
**The Gambia**	3 (2, 5)	0.7 (0.4, 1.2)	6 (4, 8)	0.6 (0.4, 0.7)	-20.6 (-43.7, 10.3)

**Ghana**	36<br>(17, 70)	0.5<br>(0.3, 0.8)	61<br>(27, 121)	0.4<br>(0.2, 0.7)	-21.8<br>(-51.1, 56.0)
**Guinea**	23<br>(7, 54)	0.5<br>(0.2, 1.0)	26<br>(11, 50)	0.4<br>(0.2, 0.8)	-19.9<br>(-38.2, 20.8)
**Guinea-Bissau**	4<br>(1, 8)	0.7<br>(0.4, 1.3)	4<br>(2, 7)	0.5<br>(0.3, 0.7)	-30.6<br>(-51.7, 22.8)
**Liberia**	6<br>(1, 15)	0.4<br>(0.1, 0.9)	8<br>(4, 13)	0.4<br>(0.2, 0.6)	-13.3<br>(-46.4, 84.0)
**Mali**	31<br>(8, 83)	0.6<br>(0.2, 1.1)	62<br>(29, 95)	0.6<br>(0.3, 1.0)	11.1<br>(-51.9, 317.3)
**Mauritania**	7<br>(4, 10)	0.6<br>(0.3, 0.8)	11<br>(7, 17)	0.5<br>(0.3, 0.8)	-14.1<br>(-53.8, 40.0)
**Niger**	33<br>(11, 80)	0.7<br>(0.4, 1.1)	54<br>(24, 90)	0.6<br>(0.3, 1.0)	-18.0<br>(-49.4, 34.5)
**Nigeria**	280<br>(99, 460)	0.5<br>(0.2, 0.9)	533<br>(217, 967)	0.6<br>(0.2, 1.1)	8.3<br>(-50.4, 94.7)
**Sao Tome and Principe**	0<br>(0, 1)	0.6<br>(0.3, 1.0)	0<br>(0, 1)	0.3<br>(0.2, 0.5)	-44.8<br>(-65.3, -19.6)
**Senegal**	26<br>(13, 43)	0.6<br>(0.4, 0.9)	50<br>(30, 77)	0.6<br>(0.4, 1.0)	1.0<br>(-36.2, 54.0)
**Sierra Leone**	13<br>(3, 33)	0.5<br>(0.1, 1.0)	21<br>(10, 37)	0.6<br>(0.3, 1.0)	17.3<br>(-45.7, 489.8)
**Togo**	9<br>(4, 18)	0.5<br>(0.3, 0.8)	19<br>(11, 29)	0.5<br>(0.3, 0.8)	-0.1<br>(-44.0, 129.3)
**Eastern sub-Saharan Africa**	537<br>(248, 985)	0.6<br>(0.4, 0.8)	1086<br>(779, 1619)	0.7<br>(0.5, 1.0)	5.6<br>(-38.5, 74.3)
**Burundi**	14<br>(5, 32)	0.6<br>(0.3, 1.0)	28<br>(11, 53)	0.7<br>(0.3, 1.3)	21.3<br>(-40.1, 211.0)
**Comoros**	2<br>(1, 4)	0.9<br>(0.4, 1.5)	3<br>(2, 6)	0.8<br>(0.5, 1.4)	-8.2<br>(-41.1, 40.8)
**Djibouti**	1<br>(1, 2)	0.7<br>(0.4, 1.0)	3<br>(2, 5)	0.5<br>(0.3, 0.8)	-19.0<br>(-46.6, 21.0)
**Eritrea**	9<br>(3, 20)	0.7<br>(0.4, 1.2)	21<br>(14, 28)	0.9<br>(0.6, 1.2)	17.7<br>(-41.8, 121.5)
**Ethiopia**	128<br>(42, 300)	0.6<br>(0.3, 1.0)	245<br>(173, 331)	0.6<br>(0.4, 0.8)	-2.5<br>(-59.0, 96.9)
**Kenya**	68<br>(41, 102)	0.8<br>(0.5, 1.2)	168<br>(107, 268)	0.8<br>(0.5, 1.3)	8.3<br>(-21.0, 56.3)
**Madagascar**	28<br>(7, 58)	0.4<br>(0.1, 0.7)	59<br>(35, 82)	0.5<br>(0.4, 0.8)	27.2<br>(-31.2, 285.4)
**Malawi**	30<br>(11, 66)	0.6<br>(0.3, 0.9)	60<br>(27, 116)	0.7<br>(0.3, 1.5)	18.2<br>(-45.5, 155.4)
**Mozambique**	60<br>(28, 110)	0.8<br>(0.5, 1.1)	96<br>(48, 179)	0.8<br>(0.4, 1.4)	-3.0<br>(-37.7, 64.3)
**Rwanda**	25<br>(5, 52)	0.8<br>(0.2, 1.2)	43<br>(19, 85)	0.7<br>(0.3, 1.5)	-2.4<br>(-55.9, 119.1)
**Somalia**	19<br>(5, 53)	0.6<br>(0.2, 1.2)	43<br>(21, 71)	0.6<br>(0.3, 1.0)	7.1<br>(-38.2, 87.0)
**South Sudan**	15<br>(5, 31)	0.6<br>(0.2, 0.9)	23<br>(10, 39)	0.5<br>(0.3, 0.8)	-2.7<br>(-31.9, 54.8)
**Tanzania**	66<br>(35, 119)	0.5<br>(0.4, 0.7)	163<br>(84, 284)	0.6<br>(0.3, 1.1)	16.2<br>(-31.2, 107.6)
**Uganda**	42<br>(11, 81)	0.5<br>(0.2, 1.1)	86<br>(41, 162)	0.6<br>(0.3, 1.2)	9.8<br>(-37.3, 121.2)
**Zambia**	30<br>(17, 54)	0.9<br>(0.7, 1.1)	44<br>(36, 54)	0.6<br>(0.5, 0.8)	-25.5<br>(-47.6, 5.4)
**Central sub-Saharan Africa**	183<br>(77, 345)	0.7<br>(0.4, 1.1)	377<br>(197, 563)	0.7<br>(0.4, 0.9)	-2.1<br>(-22.0, 29.6)
**Angola**	34<br>(6, 90)	0.7<br>(0.2, 1.4)	69<br>(32, 106)	0.6<br>(0.3, 0.9)	-11.9<br>(-48.9, 88.1)
**Central African Republic**	11<br>(4, 22)	0.8<br>(0.4, 1.4)	18<br>(9, 34)	0.8<br>(0.5, 1.3)	-4.1<br>(-28.3, 37.6)

**Congo**	8<br>(3, 16)	0.7<br>(0.3, 1.1)	16<br>(7, 24)	0.6<br>(0.3, 1.0)	-3.1<br>(-37.3, 59.1)
**DR Congo**	125<br>(57, 219)	0.7<br>(0.4, 1.0)	267<br>(138, 404)	0.7<br>(0.4, 1.0)	1.9<br>(-19.5, 35.5)
**Equatorial Guinea**	2<br>(1, 4)	0.8<br>(0.4, 1.5)	3<br>(2, 5)	0.6<br>(0.3, 1.0)	-31.6<br>(-67.0, 36.2)
**Gabon**	3<br>(1, 5)	0.5<br>(0.2, 0.9)	5<br>(1, 8)	0.4<br>(0.1, 0.8)	-10.3<br>(-42.7, 52.4)

Appendix table 4: DALYs due to IBD in 1990 and 2017 for both sexes and percentage change in age-standardised rates by location. IBD=inflammatory bowel disease. DALYs=disability-adjusted life-years.

	1990		2017		Percentage change in age-standardized rates between 1990 and 2017
	Counts (95% UI)	Rate (95% UI)	Counts (95% UI)	Rate (95% UI)	
**Global**	1247612 (965045, 1608560)	26.5 (21.0, 33.0)	1849068 (1514518, 2225092)	23.2 (19.1, 27.8)	-12.4 (-26.4, -2.6)
**High-income North America**	204385 (154897, 261521)	64.6 (48.7, 82.8)	357766 (273069, 456621)	79.8 (59.4, 103.0)	23.6 (8.2, 32.7)
**Canada**	7070 (6039, 8449)	22.8 (19.5, 27.3)	8936 (7628, 10972)	17.5 (14.6, 21.5)	-23.4 (-33.9, -9.2)
**Greenland**	8 (6, 10)	15.8 (12.4, 19.6)	11 (8, 13)	16.4 (12.4, 20.4)	4.0 (-21.2, 29.5)
**USA**	197302 (148650, 253707)	69.1 (51.8, 89.2)	348813 (265192, 446786)	86.9 (64.5, 112.4)	25.7 (10.1, 35.2)
**Australasia**	4004 (3292, 4899)	17.8 (14.6, 21.7)	7131 (5268, 8589)	18.2 (13.6, 22.3)	2.2 (-27.7, 19.4)
**Australia**	2711 (2255, 3445)	14.5 (12.0, 18.1)	5449 (3856, 6534)	16.0 (11.5, 19.5)	10.4 (-29.5, 33.0)
**New Zealand**	1293 (943, 1721)	34.6 (25.2, 46.2)	1682 (1197, 2289)	30.5 (21.4, 41.7)	-11.7 (-32.1, 11.8)
**High-income Asia-Pacific**	26042 (20694, 31226)	13.6 (10.7, 16.2)	24776 (19483, 31967)	8.8 (6.7, 11.4)	-34.9 (-45.7, -8.0)
**Brunei**	40 (31, 55)	27.2 (21.4, 35.6)	91 (77, 112)	24.2 (20.8, 29.1)	-11.0 (-33.0, 14.6)
**Japan**	17079 (13639, 20976)	11.3 (9.0, 13.9)	17039 (13362, 21670)	8.8 (6.8, 11.4)	-22.1 (-28.9, -9.2)
**Singapore**	259 (197, 323)	8.5 (6.5, 10.4)	514 (382, 673)	7.6 (5.6, 9.9)	-10.9 (-26.6, 18.2)
**South Korea**	8663 (5346, 10373)	27.7 (14.0, 33.7)	7134 (5570, 9708)	10.0 (7.7, 13.6)	-63.9 (-75.0, -9.2)
**Western Europe**	139539 (115383, 170844)	28.6 (23.4, 35.1)	242379 (174889, 290715)	37.1 (27.7, 45.7)	29.4 (-10.6, 43.8)
**Andorra**	22 (16, 31)	37.5 (27.1, 51.6)	35 (24, 47)	30.4 (21.1, 41.1)	-19.0 (-39.8, 5.5)
**Austria**	3051 (2576, 3617)	32.1 (26.9, 38.0)	3017 (2385, 3938)	24.3 (18.8, 31.2)	-24.2 (-37.8, -1.6)
**Belgium**	2981 (2435, 3712)	23.0 (18.3, 28.9)	4685 (3380, 5682)	26.8 (20.3, 33.1)	16.5 (-16.9, 38.1)
**Cyprus**	226 (157, 288)	27.9 (19.4, 35.1)	296 (236, 362)	17.8 (14.1, 21.9)	-36.4 (-51.9, -12.4)
**Denmark**	1428 (1143, 1745)	21.3 (17.0, 26.4)	1788 (1337, 2219)	21.1 (15.9, 26.8)	-1.0 (-27.1, 19)
**Finland**	1341 (960, 1800)	21.4 (15.5, 28.8)	1538 (1110, 2159)	20.0 (14.0, 29.2)	-6.8 (-31.0, 34.8)
**France**	17830 (15462, 20760)	23.9 (20.4, 28.1)	28703 (15967, 35022)	26.5 (16.8, 32.6)	11.2 (-31.1, 33.4)
**Germany**	28997 (23091, 44337)	26.9 (21.4, 40.2)	59568 (39444, 70458)	40.2 (27.8, 48.0)	49.4 (-25.3, 90.8)
**Greece**	1604 (1231, 2042)	12.8 (9.7, 16.4)	2116 (1623, 2693)	14.0 (10.3, 18.4)	9.4 (-4.9, 28.5)
**Iceland**	59 (47, 75)	21.7 (17.4, 27.9)	87 (68, 108)	19.8 (15.5, 24.8)	-8.8 (-35.3, 9.5)
**Ireland**	951 (784, 1145)	24.6 (20.2, 29.7)	1300 (969, 1603)	20.9 (15.7, 26.2)	-15.0 (-36.8, 1.3)
**Israel**	943 (757, 1174)	19.5 (15.5, 24.3)	1833 (1407, 2264)	18.7 (14.4, 23.3)	-4.0 (-20.8, 13.1)
**Italy**	14786 (11426, 19086)	20.7 (16.0, 26.7)	28281 (17168, 34739)	27.8 (17.8, 34.9)	34.7 (-20.1, 65.8)

**Luxembourg**	137<br>(113, 167)	28.2<br>(23.1, 34.3)	281<br>(180, 355)	34.0<br>(22.4, 43.0)	20.4<br>(-22.7, 45.6)
**Malta**	70<br>(57, 86)	17.1<br>(14.0, 20.9)	140<br>(99, 170)	21.3<br>(15.4, 26.2)	24.4<br>(-14.2, 46.9)
**Netherlands**	5920<br>(5069, 7344)	33.4<br>(28.7, 40.7)	9615<br>(6559, 11331)	35.0<br>(24.9, 42.3)	4.8<br>(-34.1, 26.0)
**Norway**	4594<br>(3142, 6364)	91.9<br>(62.0, 127)	4077<br>(2752, 5701)	61.5<br>(41.5, 87.2)	-33.1<br>(-51.3, -10.1)
**Portugal**	2043<br>(1743, 2435)	16.9<br>(14.4, 20.3)	4051<br>(2118, 4956)	21.1<br>(12.2, 26.1)	25.2<br>(-33.8, 54.0)
**Spain**	7594<br>(6325, 9229)	15.8<br>(13.2, 19.6)	9918<br>(7885, 11966)	14.2<br>(10.9, 17.7)	-10.2<br>(-29.6, 5.6)
**Sweden**	3100<br>(2490, 3794)	26.7<br>(21.0, 33.2)	2975<br>(2277, 3882)	21.4<br>(15.8, 28.7)	-19.8<br>(-33.0, -0.2)
**Switzerland**	2157<br>(1637, 2680)	24.1<br>(18.5, 30.1)	2880<br>(2197, 3608)	22.5<br>(17.0, 28.9)	-6.6<br>(-22.5, 12.0)
**United Kingdom**	39572<br>(31418, 49415)	55.2<br>(43.1, 69.4)	74946<br>(56920, 94826)	86.8<br>(65.0, 111.5)	57.1<br>(31.4, 72.2)
**Southern Latin America**	7208<br>(6050, 8461)	15.0<br>(12.6, 17.6)	10205<br>(8427, 12340)	13.6<br>(11.2, 16.5)	-9.0<br>(-21.9, 3.7)
**Argentina**	4659<br>(3922, 5519)	14.2<br>(12.0, 16.9)	6487<br>(5325, 7874)	13.2<br>(10.8, 16.1)	-7.1<br>(-21.1, 7.4)
**Chile**	2073<br>(1663, 2474)	17.5<br>(14.1, 20.8)	3013<br>(2402, 3779)	14.1<br>(11.3, 17.7)	-19.3<br>(-31.9, 3.5)
**Uruguay**	475<br>(379, 656)	13.5<br>(10.7, 18.6)	705<br>(553, 835)	16.2<br>(12.7, 19.3)	19.8<br>(-22.0, 46.5)
**Eastern Europe**	103435<br>(79989, 127537)	39.3<br>(30.3, 48.6)	82880<br>(66636, 99430)	29.0<br>(23.3, 35.1)	-26.2<br>(-31.5, -19.1)
**Belarus**	3929<br>(3052, 4953)	32.6<br>(25.2, 41.2)	3759<br>(2796, 4777)	28.8<br>(21.1, 36.8)	-11.6<br>(-24.9, 1.1)
**Estonia**	770<br>(618, 940)	42.1<br>(33.7, 51.6)	478<br>(374, 596)	25.7<br>(19.6, 32.6)	-38.8<br>(-47.6, -23.8)
**Latvia**	984<br>(771, 1224)	30.2<br>(23.7, 37.4)	750<br>(589, 928)	26.5<br>(20.5, 33.4)	-12.2<br>(-26.1, -0.2)
**Lithuania**	1303<br>(1014, 1651)	30.3<br>(23.6, 38.7)	1055<br>(810, 1323)	25.6<br>(19.5, 32.6)	-15.6<br>(-27.9, -4.7)
**Moldova**	1332<br>(1056, 1626)	28.9<br>(22.9, 35.3)	1219<br>(962, 1494)	25.2<br>(19.7, 31.1)	-12.9<br>(-22.9, -3.6)
**Russia**	75260<br>(57217, 93250)	43.4<br>(32.7, 53.9)	57301<br>(46371, 68631)	28.8<br>(2.03, 34.7)	-33.6<br>(-39.8, -26.1)
**Ukraine**	19856<br>(15285, 25195)	31.3<br>(24.0, 40.1)	18318<br>(14679, 22291)	30.7<br>(24.2, 37.7)	-1.7<br>(-15.9, 14.8)
**Central Europe**	49828<br>(39622, 60970)	37.2<br>(30.0, 45.2)	54017<br>(41986, 67207)	34.4<br>(26.3, 43.3)	-7.5<br>(-20.1, -0.2)
**Albania**	3633<br>(1183, 4685)	103.7<br>(42.1, 131.2)	1145<br>(821, 1545)	36.3<br>(26.1, 48.1)	-65.0<br>(-75.4, -6.7)
**Bosnia and Herzegovina**	1059<br>(794, 1345)	22.8<br>(17.1, 28.8)	1098<br>(820, 1389)	23.1<br>(17.4, 29.5)	1.1<br>(-9.7, 13.6)
**Bulgaria**	2955<br>(2193, 3952)	28.8<br>(21.4, 38.2)	2999<br>(2188, 4008)	31.1<br>(21.9, 41.8)	8.2<br>(-10.3, 24.5)
**Croatia**	2061<br>(1526, 2649)	35.7<br>(26.5, 46.4)	2242<br>(1693, 2865)	37.6<br>(27.7, 48.8)	5.2<br>(-15.9, 19.7)
**Czech Republic**	4874<br>(3822, 6088)	40.4<br>(31.5, 50.9)	5670<br>(4158, 7081)	37.7<br>(27.8, 47.4)	-6.6<br>(-30.8, 6.7)
**Hungary**	3886<br>(3239, 4915)	31.4<br>(26.1, 39.4)	3470<br>(2895, 4110)	25.2<br>(20.7, 30.4)	-19.8<br>(-41.4, -9.3)
**Macedonia**	512<br>(361, 690)	24.9<br>(17.7, 33.8)	791<br>(546, 1085)	28.6<br>(19.7, 39.3)	14.6<br>(-0.8, 29.2)
**Montenegro**	254<br>(183, 347)	39.4<br>(28.5, 53)	248<br>(179, 331)	31.3<br>(22.4, 42.2)	-20.5<br>(-29.8, -10.1)
**Poland**	16165<br>(12276, 20730)	37.8<br>(28.7, 48.8)	20153<br>(15474, 25474)	38.2<br>(29.2, 49.2)	1.0<br>(-21.9, 15.9)

**Romania**	7237 (5606, 9208)	28.9 (22.2, 36.5)	7036 (5376, 8946)	27.2 (20.6, 34.8)	-6.1 (-21.4, 6.8)
**Serbia**	3776 (2937, 4727)	35.6 (28.1, 44.7)	5014 (3862, 6083)	40.6 (31.5, 50.2)	13.8 (-18.1, 28.0)
**Slovakia**	2478 (1908, 3190)	43.8 (33.9, 56.3)	3148 (2482, 3857)	44.3 (34.8, 54.7)	1.1 (-23.7, 19.5)
**Slovenia**	937 (714, 1194)	41.0 (31.2, 52.5)	1002 (758, 1277)	34.1 (25.1, 44.3)	-16.8 (-27.3, -8.4)
**Central Asia**	12720 (10388, 16690)	19.7 (16.3, 24.3)	13504 (10767, 15938)	15.2 (12.1, 17.8)	-23.1 (-39.9, -11.4)
**Armenia**	451 (366, 550)	14.0 (11.4, 16.9)	454 (354, 547)	12.4 (9.7, 15.0)	-11.2 (-27.5, 2.7)
**Azerbaijan**	600 (474, 748)	9.4 (7.5, 11.7)	979 (771, 1219)	9.1 (7.2, 11.3)	-3.9 (-20.1, 14.2)
**Georgia**	717 (548, 870)	12.0 (9.3, 14.6)	560 (416, 683)	11.5 (8.9, 14.2)	-3.9 (-13.8, 6.6)
**Kazakhstan**	3658 (2996, 4431)	24.0 (19.8, 28.7)	3814 (2740, 4602)	20.5 (14.7, 24.7)	-14.5 (-39.0, 5.4)
**Kyrgyzstan**	1079 (568, 1405)	25.7 (15.4, 32.1)	809 (618, 982)	13.5 (10.7, 16.2)	-47.3 (-62.5, -11.4)
**Mongolia**	585 (300, 1061)	31.1 (17.7, 46.3)	593 (418, 833)	18.8 (13.4, 26.2)	-39.6 (-62.5, -12.8)
**Tajikistan**	1457 (736, 3077)	23.6 (15.6, 40.5)	1381 (1108, 1693)	16.6 (13.6, 20.1)	-29.5 (-61.1, 8.3)
**Turkmenistan**	619 (401, 937)	18.9 (13.6, 25.2)	723 (575, 882)	14.9 (12.1, 17.9)	-20.8 (-35.9, -0.9)
**Uzbekistan**	3555 (2046, 4702)	17.5 (12.6, 21.3)	4192 (3181, 5128)	13.6 (10.7, 16.4)	-22.2 (-39.3, -2.4)
**Central Latin America**	14785 (13008, 16993)	12.0 (10.6, 13.8)	31188 (23681, 36238)	12.7 (9.7, 14.8)	5.5 (-21.7, 16.8)
**Colombia**	2945 (2494, 3422)	11.6 (10.0, 13.4)	6083 (4241, 7373)	11.6 (8.0, 14.0)	-0.2 (-31.7, 17.3)
**Costa Rica**	272 (210, 343)	12.5 (9.7, 15.9)	462 (341, 582)	9.3 (6.9, 11.6)	-25.8 (-40.0, -10.1)
**El Salvador**	628 (318, 866)	14.3 (8.4, 18.1)	423 (338, 535)	7.3 (5.8, 9.3)	-48.8 (-62.7, -0.5)
**Guatemala**	644 (392, 1465)	8.7 (6.1, 16.6)	1618 (1041, 2028)	11.4 (7.7, 14.1)	30.5 (-49.5, 100.6)
**Honduras**	757 (482, 1202)	19.8 (14.2, 26.6)	1502 (997, 2129)	20.3 (13.7, 28.1)	2.4 (-39.7, 54.2)
**Mexico**	7367 (6552, 8797)	12.1 (10.7, 14.3)	17455 (13185, 20248)	14.4 (10.9, 16.6)	19.0 (-14.1, 32.9)
**Nicaragua**	188 (147, 242)	7.4 (6.0, 8.9)	389 (291, 493)	7.3 (5.5, 9.3)	-1.7 (-23.5, 19.8)
**Panama**	391 (317, 499)	18.2 (14.6, 23.9)	949 (581, 1210)	24.6 (14.8, 31.6)	35.2 (-32.3, 93.7)
**Venezuela**	1593 (1116, 1911)	11.3 (8.1, 13.6)	2307 (1864, 2838)	7.6 (6.2, 9.3)	-32.8 (-45.1, -7.2)
**Andean Latin America**	11018 (6264, 21547)	26.1 (16.9, 45.7)	5544 (4506, 6596)	9.4 (7.7, 11.0)	-64.2 (-82.1, -42.0)
**Bolivia**	1640 (530, 4589)	22.2 (9.7, 53.3)	1402 (927, 2018)	12.9 (9.2, 17.7)	-41.9 (-78.8, 52.3)
**Ecuador**	1183 (880, 1540)	12.1 (9.5, 15.1)	1350 (956, 1625)	8.6 (6.1, 10.4)	-28.8 (-54.3, -6.7)
**Peru**	8196 (4306, 16681)	33.6 (20.5, 61.4)	2792 (2222, 3485)	8.6 (6.9, 10.6)	-74.6 (-87.8, -53.3)
**Caribbean**	5917 (4223, 10018)	18.5 (13.8, 29.4)	5969 (4877, 7769)	12.4 (10.0, 16.5)	-33.3 (-50.3, -17.2)
**Antigua and Barbuda**	13 (9, 14)	22.9 (16.8, 26.5)	13 (11, 16)	13.1 (11.2, 16.7)	-43.0 (-55.0, -10.1)
**The Bahamas**	47 (37, 56)	21.6 (17.1, 26.0)	63 (55, 74)	16.1 (13.9, 18.8)	-25.6 (-40.3, -7.1)

**Barbados**	38<br>(32, 45)	14.1<br>(12.0, 16.9)	42<br>(36, 48)	10.6<br>(9.1, 12.3)	-24.7<br>(-37.4, -7.9)
**Belize**	38<br>(23, 53)	24.5<br>(16.4, 30.8)	60<br>(50, 74)	17.8<br>(14.9, 22.7)	-27.3<br>(-48.3, 35.4)
**Bermuda**	15<br>(11, 18)	23.5<br>(17.1, 28.7)	10<br>(8, 13)	10.4<br>(8.7, 13.7)	-55.5<br>(-67.0, -25.3)
**Cuba**	1256<br>(808, 1526)	11.7<br>(7.5, 14.2)	998<br>(761, 1168)	6.2<br>(4.9, 7.2)	-47.0<br>(-56.1, -27.2)
**Dominica**	15<br>(13, 18)	22.1<br>(19.1, 26.3)	16<br>(14, 20)	20.7<br>(17.8, 25.6)	-6.5<br>(-26.9, 18.7)
**Dominican Republic**	553<br>(368, 885)	8.7<br>(6.3, 11.9)	701<br>(530, 1051)	7.1<br>(5.4, 10.5)	-18.9<br>(-40.1, 27.2)
**Grenada**	29<br>(23, 37)	39.0<br>(30.8, 50.4)	33<br>(29, 41)	25.9<br>(22.6, 31.5)	-33.6<br>(-50.3, -8.5)
**Guyana**	183<br>(148, 213)	30.0<br>(25.2, 34.2)	168<br>(142, 197)	24.2<br>(20.6, 28.2)	-19.3<br>(-32.4, -2.0)
**Haiti**	2007<br>(549, 5812)	34.3<br>(12.3, 78.9)	2430<br>(1386, 3931)	26.8<br>(16.2, 38.4)	-21.8<br>(-58.3, 67.3)
**Jamaica**	326<br>(260, 421)	14.9<br>(12.4, 18.6)	394<br>(310, 492)	14.1<br>(10.9, 17.4)	-5.6<br>(-28.4, 22.5)
**Puerto Rico**	929<br>(537, 1077)	25.5<br>(14.7, 29.6)	486<br>(396, 721)	9.6<br>(7.8, 13.9)	-62.5<br>(-72.3, -13.8)
**Saint Lucia**	27<br>(19, 34)	24.2<br>(17.5, 30.0)	31<br>(27, 37)	15.4<br>(13.5, 18.4)	-36.3<br>(-50.9, -1.6)
**Saint Vincent and the Grenadines**	14<br>(9, 36)	16.4<br>(10.5, 41.8)	37<br>(33, 42)	29.4<br>(25.7, 33.3)	79.4<br>(-30.6, 192.5)
**Suriname**	98<br>(69, 118)	28.9<br>(21.0, 34.1)	118<br>(103, 141)	20.2<br>(17.6, 24.1)	-30.2<br>(-44.5, 3.0)
**Trinidad and Tobago**	110<br>(72, 133)	10.9<br>(6.9, 13.2)	138<br>(90, 174)	8.2<br>(5.4, 10.3)	-25.1<br>(-42.5, -4.0)
**Virgin Islands**	18<br>(14, 21)	18.2<br>(14.6, 21.1)	16<br>(14, 21)	11.2<br>(9.2, 14.3)	-38.5<br>(-52.3, -12.9)
**Tropical Latin America**	27268<br>(23259, 32163)	23.0<br>(19.6, 27.1)	44620<br>(35941, 52406)	19.1<br>(15.3, 22.4)	-17.0<br>(-29.6, -9.2)
**Brazil**	26869<br>(22934, 31655)	23.2<br>(19.7, 27.4)	43834<br>(35188, 51386)	19.2<br>(15.4, 22.6)	-17.1<br>(-29.9, -9.3)
**Paraguay**	399<br>(314, 493)	13.5<br>(10.4, 16.9)	785<br>(617, 991)	13.2<br>(10.4, 16.4)	-2.7<br>(-18.4, 12.4)
**East Asia**	362502<br>(267423, 459808)	33.8<br>(25.2, 41.5)	524890<br>(397470, 674531)	27.4<br>(2.01, 34.7)	-19.1<br>(-36.1, 3.7)
**China**	346396<br>(253846, 440768)	34.0<br>(25.3, 41.8)	502261<br>(380117, 646618)	27.5<br>(21.1, 35.0)	-19.1<br>(-36.5, 4.3)
**North Korea**	5262<br>(4019, 6591)	28.5<br>(21.9, 35.3)	6364<br>(4887, 8092)	22.4<br>(17.3, 28)	-21.6<br>(-35.4, -5.5)
**Taiwan (Province of China)**	4804<br>(3727, 5970)	27.7<br>(21.3, 33.9)	7809<br>(5977, 10045)	22.7<br>(17.3, 29.5)	-17.9<br>(-30.6, 4.7)
**Southeast Asia**	54475<br>(39376, 75926)	15.6<br>(11.7, 19.5)	63437<br>(54372, 73751)	10.1<br>(8.7, 11.8)	-35.1<br>(-50.4, -13.9)
**Cambodia**	1141<br>(576, 2359)	16.2<br>(9.9, 26.9)	1593<br>(1252, 1942)	12.2<br>(9.7, 14.7)	-24.7<br>(-55.8, 24.8)
**Indonesia**	24652<br>(16116, 37090)	19.4<br>(13.6, 25.4)	29848<br>(24367, 37879)	13.3<br>(10.9, 17.1)	-31.2<br>(-50.2, -8.3)
**Laos**	448<br>(192, 937)	14.5<br>(7.4, 24.5)	699<br>(474, 953)	13.2<br>(9.1, 18.7)	-9.0<br>(-51.6, 51.2)
**Malaysia**	1048<br>(842, 1288)	8.3<br>(6.8, 9.8)	2246<br>(1813, 2717)	7.8<br>(6.4, 9.5)	-5.1<br>(-21.2, 21.4)
**Maldives**	20<br>(10, 40)	15.3<br>(9.2, 25.1)	32<br>(25, 42)	8.7<br>(6.7, 10.8)	-42.8<br>(-70.4, 3.8)
**Mauritius**	87<br>(68, 110)	9.1<br>(7.2, 11.5)	120<br>(86, 151)	7.9<br>(5.5, 9.9)	-13.8<br>(-37.9, 5.6)
**Myanmar**	3807<br>(1784, 8154)	11.6<br>(6.1, 22.3)	4582<br>(3506, 5682)	9.3<br>(7.2, 11.6)	-20.4<br>(-55.9, 40.4)

**Philippines**	10519<br>(5836, 16205)	20.6<br>(12.8, 27.6)	7972<br>(6140, 12965)	9.1<br>(7.1, 14.7)	-55.7<br>(-71.6, 3.5)
**Sri Lanka**	844<br>(630, 1128)	6.0<br>(4.7, 7.7)	1072<br>(836, 1346)	4.5<br>(3.5, 5.6)	-25.2<br>(-46.7, -1.9)
**Seychelles**	8<br>(6, 9)	12.4<br>(9.8, 14.8)	10<br>(8, 13)	9.0<br>(7.3, 11.2)	-27.7<br>(-38.4, -13.8)
**Thailand**	4602<br>(3411, 5697)	9.2<br>(7.0, 11.1)	6542<br>(5351, 7719)	7.3<br>(6.0, 8.5)	-21.1<br>(-37.9, 5.5)
**East Timor**	83<br>(43, 155)	15.6<br>(9.6, 20.6)	158<br>(94, 237)	17.1<br>(10.0, 26.1)	9.7<br>(-44.0, 80.0)
**Vietnam**	7146<br>(5009, 8882)	14.6<br>(9.9, 18.5)	8478<br>(6985, 10270)	8.8<br>(7.2, 10.5)	-39.7<br>(-54.4, -15.8)
**Oceania**	861<br>(548, 1428)	19.1<br>(12.9, 27.6)	1415<br>(1040, 2009)	14.9<br>(11.5, 19.5)	-21.9<br>(-39.4, 2.3)
**American Samoa**	16<br>(10, 19)	49.9<br>(30.0, 62.8)	9<br>(7, 11)	17.8<br>(14.6, 23.7)	-64.4<br>(-73.5, -24.4)
**Federated States of Micronesia**	19<br>(11, 28)	29.9<br>(18.1, 40.7)	16<br>(10, 22)	19.3<br>(1.03, 25.9)	-35.6<br>(-54.3, -7.6)
**Fiji**	57<br>(46, 70)	10.7<br>(8.8, 12.9)	85<br>(69, 104)	10.1<br>(8.2, 12.2)	-5.9<br>(-25.2, 17.3)
**Guam**	17<br>(13, 22)	15.8<br>(12.3, 20.0)	18<br>(14, 23)	10.1<br>(7.9, 12.8)	-36.0<br>(-49.6, -18.5)
**Kiribati**	16<br>(6, 32)	29.5<br>(13.6, 50.7)	20<br>(11, 36)	23.5<br>(12.9, 37.0)	-20.5<br>(-41.5, 12.1)
**Marshall Islands**	6<br>(4, 12)	26.0<br>(16.1, 43.9)	8<br>(5, 13)	19.9<br>(13.5, 29.7)	-23.3<br>(-42.3, 8.8)
**Northern Mariana Islands**	12<br>(8, 15)	35.8<br>(24.9, 44.7)	8<br>(6, 10)	14.8<br>(12.1, 19.0)	-58.7<br>(-69.2, -27.3)
**Papua New Guinea**	522<br>(282, 1007)	17.2<br>(10.7, 28.8)	957<br>(628, 1523)	13.9<br>(9.8, 20.0)	-19.6<br>(-43.3, 15.6)
**Samoa**	25<br>(13, 35)	23.9<br>(12.9, 34.3)	27<br>(18, 40)	17.6<br>(11.5, 25.2)	-26.4<br>(-46.9, 2.0)
**Solomon Islands**	35<br>(21, 62)	17.4<br>(10.9, 27.6)	58<br>(42, 78)	13.1<br>(9.8, 17.0)	-25.1<br>(-48.8, 8.7)
**Tonga**	20<br>(14, 26)	30.0<br>(20.9, 40.0)	21<br>(16, 27)	23.6<br>(18.2, 31.4)	-21.3<br>(-38.4, 4.8)
**Vanuatu**	60<br>(28, 99)	63.3<br>(29.3, 105.4)	111<br>(54, 192)	53.6<br>(26.1, 92.7)	-15.4<br>(-48.0, 32.2)
**North Africa and Middle East**	29305<br>(20321, 44059)	11.3<br>(8.2, 15.9)	53148<br>(43636, 64294)	10.0<br>(8.3, 11.9)	-12.1<br>(-35.4, 16.7)
**Afghanistan**	765<br>(330, 1568)	9.8<br>(4.5, 19.2)	2101<br>(1264, 3484)	11.5<br>(7.1, 18.3)	17.2<br>(-22.4, 100.2)
**Algeria**	1353<br>(982, 1943)	8.0<br>(5.7, 11.5)	3108<br>(2203, 4253)	8<br>(5.7, 10.9)	0.1<br>(-21.3, 22.0)
**Bahrain**	46<br>(35, 61)	11.5<br>(9.0, 14.4)	125<br>(89, 167)	7.9<br>(6.0, 10.1)	-31.4<br>(-42.6, -18.2)
**Egypt**	3035<br>(2109, 4666)	7.3<br>(5.4, 10.5)	6531<br>(4522, 9090)	8.1<br>(5.6, 11.3)	10.3<br>(-16.3, 49.7)
**Iran**	2998<br>(2303, 3800)	7.6<br>(6.0, 9.4)	7116<br>(5201, 8787)	8.6<br>(6.2, 10.5)	13.8<br>(-8.3, 32.9)
**Iraq**	2429<br>(1365, 4003)	16.5<br>(10.7, 25.2)	4344<br>(3168, 5580)	11.3<br>(9.0, 14)	-31.7<br>(-59.0, 9.5)
**Jordan**	346<br>(240, 500)	14.6<br>(10.3, 21.1)	881<br>(673, 1129)	10.3<br>(7.9, 12.9)	-29.7<br>(-57.4, -2.6)
**Kuwait**	140<br>(110, 177)	9.4<br>(7.5, 11.5)	360<br>(266, 483)	8.1<br>(6.1, 10.5)	-13.2<br>(-28.7, 3.5)
**Lebanon**	180<br>(125, 264)	6.0<br>(4.2, 9.0)	438<br>(334, 569)	5.8<br>(4.4, 7.5)	-3.7<br>(-31.5, 46.9)
**Libya**	212<br>(154, 303)	7.7<br>(5.6, 11.1)	416<br>(292, 545)	6.7<br>(4.8, 8.8)	-13.0<br>(-32.7, 10.3)
**Morocco**	1406<br>(1001, 2059)	7.7<br>(5.5, 11.1)	3012<br>(2290, 3940)	8.6<br>(6.6, 11.2)	12.7<br>(-9.8, 42.9)

**Palestine**	145<br>(95, 214)	11.7<br>(8.0, 16.3)	308<br>(232, 409)	8.8<br>(6.8, 11.4)	-24.5<br>(-48.8, 3.4)
**Oman**	107<br>(74, 152)	8.5<br>(6.0, 11.8)	284<br>(201, 393)	6.9<br>(5.1, 9.1)	-18.6<br>(-39.6, 9.8)
**Qatar**	62<br>(46, 80)	20.5<br>(15.4, 25.1)	309<br>(201, 464)	12.6<br>(9.0, 17.5)	-38.5<br>(-57.9, -13.1)
**Saudi Arabia**	833<br>(610, 1095)	7.7<br>(5.7, 9.9)	2126<br>(1558, 2935)	6.4<br>(4.7, 8.6)	-17.0<br>(-34.8, 10.0)
**Sudan**	916<br>(333, 2031)	6.5<br>(2.4, 12.0)	2093<br>(1279, 2956)	7.7<br>(4.7, 11.0)	19.9<br>(-27.3, 114.5)
**Syria**	1089<br>(766, 1582)	14.3<br>(10.4, 19.5)	1675<br>(1245, 2108)	11.4<br>(8.2, 14.2)	-20.3<br>(-45.6, 11.1)
**Tunisia**	510<br>(367, 710)	7.9<br>(5.7, 11.0)	1017<br>(731, 1361)	8.1<br>(5.9, 10.8)	2.4<br>(-17.2, 28.8)
**Turkey**	12049<br>(7015, 21920)	23.7<br>(14.3, 39.8)	14218<br>(11814, 17042)	16.8<br>(14.0, 20.0)	-29.5<br>(-58.4, 18.0)
**United Arab Emirates**	126<br>(84, 189)	8.8<br>(5.9, 13.1)	881<br>(585, 1327)	7.9<br>(5.6, 10.7)	-10.1<br>(-35.5, 27.0)
**Yemen**	537<br>(276, 1004)	6.8<br>(3.8, 11.3)	1755<br>(1244, 2392)	9.1<br>(6.6, 12.2)	33.0<br>(-14.4, 107.4)
**South Asia**	124731<br>(76459, 212020)	14.6<br>(9.9, 21.9)	208064<br>(168173, 245342)	13.5<br>(11.0, 15.8)	-7.5<br>(-31.9, 22.3)
**Bangladesh**	13796<br>(7309, 28740)	15.6<br>(10.3, 24.6)	19030<br>(15586, 22968)	13.5<br>(11.2, 16.2)	-13.3<br>(-49.0, 42.7)
**Bhutan**	67<br>(40, 108)	17.7<br>(11.9, 23.2)	107<br>(66, 153)	13.9<br>(8.6, 19.9)	-21.2<br>(-57.6, 28.0)
**India**	93715<br>(55825, 166147)	13.7<br>(9.0, 22.0)	156388<br>(122074, 196066)	12.8<br>(10.1, 16.0)	-6.6<br>(-31.6, 28.9)
**Nepal**	2183<br>(1241, 3896)	15.8<br>(10.2, 23.2)	3117<br>(2282, 4033)	12.8<br>(9.5, 16.7)	-18.9<br>(-53.4, 22.5)
**Pakistan**	14971<br>(10225, 19458)	20.1<br>(14.1, 27.8)	29422<br>(21097, 41691)	19.8<br>(12.9, 29.2)	-1.5<br>(-27.1, 29.9)
**Southern sub-Saharan Africa**	7464<br>(5998, 8680)	17.8<br>(14.4, 21.3)	9609<br>(7498, 11437)	14.1<br>(11.2, 16.7)	-20.5<br>(-33.4, -1.9)
**Botswana**	194<br>(125, 278)	20.5<br>(13.7, 29.0)	260<br>(151, 379)	13.8<br>(8.7, 19.3)	-32.6<br>(-48.3, -10.2)
**Lesotho**	244<br>(165, 352)	18.4<br>(12.4, 26.8)	262<br>(177, 376)	17.0<br>(11.4, 24.7)	-7.1<br>(-37.8, 31.5)
**Namibia**	227<br>(166, 303)	21.6<br>(16.2, 28.7)	370<br>(196, 586)	19.2<br>(10.8, 29.9)	-11.5<br>(-47.4, 33.2)
**South Africa**	5632<br>(4285, 6644)	18.5<br>(14.3, 22.6)	6921<br>(4888, 8697)	13.7<br>(9.7, 17.1)	-25.6<br>(-44.3, -6.6)
**Swaziland**	91<br>(59, 135)	15.9<br>(10.7, 21.7)	124<br>(91, 166)	14.1<br>(10.1, 18.8)	-11.0<br>(-34.3, 28.5)
**Zimbabwe**	1076<br>(757, 1534)	14.2<br>(10.9, 18.3)	1672<br>(962, 2779)	15.1<br>(8.6, 25.4)	6.6<br>(-43.4, 60.5)
**Western sub-Saharan Africa**	27444<br>(10137, 61485)	17.0<br>(8.0, 28.9)	46246<br>(29571, 65815)	15.8<br>(11.2, 20.6)	-7.5<br>(-48.3, 60.3)
**Benin**	640<br>(213, 1640)	16.2<br>(6.9, 30.6)	1052<br>(704, 1599)	14.0<br>(10.1, 19.1)	-13.4<br>(-54.1, 85.4)
**Burkina Faso**	1432<br>(544, 3502)	17.5<br>(9.1, 32.2)	2341<br>(917, 4463)	15.9<br>(6.9, 28.2)	-9.1<br>(-48.3, 79.1)
**Cameroon**	1290<br>(514, 2759)	16.7<br>(8.1, 27.7)	2444<br>(1176, 3709)	13.9<br>(.07, 21.2)	-16.4<br>(-49.9, 49.9)
**Cape Verde**	38<br>(25, 52)	13.7<br>(9.5, 17.3)	43<br>(35, 51)	8.7<br>(7.1, 10.4)	-36.5<br>(-50.8, -11.5)
**Chad**	952<br>(374, 2146)	20.0<br>(10.0, 33.5)	1658<br>(922, 3165)	16.8<br>(11.3, 23.7)	-15.7<br>(-40.6, 30.5)
**Cote d'Ivoire**	1259<br>(517, 2819)	14.7<br>(7.3, 27.3)	2487<br>(1602, 3621)	15.0<br>(10.4, 19.7)	1.9<br>(-38.0, 87.0)
**The Gambia**	141<br>(72, 268)	21.1<br>(12.8, 34.0)	221<br>(156, 305)	16.2<br>(11.7, 21.0)	-23.5<br>(-46.4, 9.7)

**Ghana**	1589<br>(702, 3441)	14.7<br>(7.6, 27.5)	2491<br>(1292, 4702)	11.5<br>(6.0, 21.3)	-21.9<br>(-52.1, 54.8)
**Guinea**	1121<br>(262, 3298)	18.3<br>(6.0, 42.1)	1094<br>(466, 2311)	13.1<br>(6.2, 24.2)	-28.2<br>(-48.3, 22.8)
**Guinea-Bissau**	172<br>(56, 482)	21.8<br>(9.8, 47.5)	178<br>(98, 317)	14.6<br>(8.7, 23.4)	-33.0<br>(-57, .0 29.3)
**Liberia**	305<br>(67, 883)	15.2<br>(4.4, 36.3)	374<br>(180, 612)	11.6<br>(5.8, 17.2)	-24.1<br>(-60.4, 76.7)
**Mali**	1689<br>(321, 5342)	19.5<br>(5.5, 48.3)	2854<br>(1178, 5724)	19.2<br>(9.9, 29.0)	-1.3<br>(-56.7, 276.4)
**Mauritania**	263<br>(133, 449)	17.7<br>(9.9, 25.9)	413<br>(257, 595)	15.4<br>(9.5, 22.5)	-13.4<br>(-54.3, 41.4)
**Niger**	1837<br>(435, 5802)	24.7<br>(10.3, 52.1)	2481<br>(1031, 4929)	18.3<br>(9.4, 29.2)	-25.9<br>(-61.4, 41.0)
**Nigeria**	12454<br>(4060, 27553)	16.4<br>(6.5, 27.2)	22591<br>(10234, 35856)	16.3<br>(7.6, 28.4)	-0.8<br>(-54.7, 87.1)
**Sao Tome and Principe**	21<br>(8, 45)	20.3<br>(8.8, 33.9)	17<br>(10, 24)	10.9<br>(6.6, 15.3)	-46.4<br>(-63.6, -15.7)
**Senegal**	1142<br>(489, 2461)	19.5<br>(10.6, 30.9)	1839<br>(1166, 2747)	18.6<br>(12.0, 28.3)	-4.8<br>(-47.1, 54.3)
**Sierra Leone**	646<br>(122, 2000)	16.9<br>(4.3, 43.5)	889<br>(419, 1584)	16.6<br>(8.7, 27.8)	-1.9<br>(-56.3, 347.1)
**Togo**	452<br>(176, 1081)	16.4<br>(8.2, 29.6)	779<br>(482, 1170)	15.4<br>(9.7, 22.4)	-6.1<br>(-50.1, 110.4)
**Eastern sub-Saharan Africa**	25963<br>(9701, 58842)	17.6<br>(9.1, 30.0)	45464<br>(30913, 68543)	17.6<br>(13.0, 25.7)	0.0<br>(-47.6, 76.7)
**Burundi**	704<br>(216, 1934)	15.8<br>(6.7, 34.0)	1228<br>(475, 2253)	17.8<br>(7.9, 32.3)	12.9<br>(-46.9, 230.6)
**Comoros**	83<br>(35, 167)	24.7<br>(11.7, 45.6)	120<br>(78, 202)	21.4<br>(13.9, 36.0)	-13.6<br>(-47.9, 38.3)
**Djibouti**	65<br>(28, 128)	19.4<br>(10.5, 29.0)	124<br>(77, 179)	15.1<br>(9.6, 22.0)	-22.4<br>(-53.9, 22.8)
**Eritrea**	454<br>(143, 1211)	21.8<br>(9.6, 44.5)	949<br>(623, 1295)	24.3<br>(17.1, 32.3)	11.3<br>(-47.6, 130.5)
**Ethiopia**	6606<br>(1869, 17916)	16.8<br>(7.0, 34.8)	10182<br>(7814, 13140)	15.4<br>(11.2, 20.5)	-8.2<br>(-64.9, 114.2)
**Kenya**	2829<br>(1569, 4055)	20.1<br>(12.6, 29.1)	6186<br>(4329, 9480)	20.7<br>(13.8, 32.2)	3.2<br>(-27.6, 48.1)
**Madagascar**	1426<br>(409, 3419)	14.1<br>(4.9, 27.8)	2658<br>(1549, 3999)	15.3<br>(9.8, 20.7)	8.9<br>(-42.2, 191.1)
**Malawi**	1477<br>(333, 4396)	17.6<br>(6.9, 36.6)	2286<br>(1053, 4166)	19.8<br>(9.3, 37.7)	12.4<br>(-58.9, 200.5)
**Mozambique**	2844<br>(1038, 7032)	24.5<br>(12.2, 42.0)	4215<br>(2181, 8227)	22.1<br>(11.7, 39.8)	-9.7<br>(-47.4, 61.5)
**Rwanda**	1168<br>(209, 3035)	20.5<br>(5.1, 40.5)	1705<br>(785, 3283)	19.6<br>(9.4, 37.6)	-4.6<br>(-62.6, 147.8)
**Somalia**	1026<br>(200, 3295)	17.6<br>(5.6, 43.7)	2042<br>(1014, 3599)	17.8<br>(9.5, 28.0)	1.1<br>(-50.2, 125.3)
**South Sudan**	667<br>(183, 1718)	15.6<br>(5.8, 31.5)	1115<br>(459, 2171)	15.8<br>(7.6, 25.1)	1.1<br>(-33.6, 73.2)
**Tanzania**	3164<br>(1242, 7494)	15.1<br>(8.7, 25.5)	6964<br>(3575, 11682)	17.3<br>(9.6, 29.0)	15.0<br>(-39.1, 115.4)
**Uganda**	2028<br>(448, 5231)	14.7<br>(4.8, 26.7)	3689<br>(1826, 6459)	15.5<br>(8.1, 28.8)	5.7<br>(-48.8, 123.5)
**Zambia**	1411<br>(582, 3403)	23.7<br>(15.1, 39.1)	1973<br>(1449, 2770)	16.9<br>(14.0, 20.2)	-28.7<br>(-58.2, 15.3)
**Central sub-Saharan Africa**	8718<br>(3250, 18723)	20.7<br>(9.6, 37.0)	16817<br>(8461, 26581)	19.7<br>(10.9, 28.7)	-5.1<br>(-31.1, 35.1)
**Angola**	1777<br>(323, 5316)	21.3<br>(5.2, 51.7)	3238<br>(1600, 4994)	17.0<br>(8.6, 25.3)	-20.0<br>(-59.1, 96.9)
**Central African Republic**	502<br>(174, 1146)	25.2<br>(10.7, 48.9)	781<br>(337, 1600)	23.7<br>(12.0, 44.0)	-5.8<br>(-29.2, 38.0)

**Congo**	362<br>(145, 761)	19.7<br>(8.9, 37.3)	622<br>(315, 966)	17.4<br>(9.0, 26.5)	-11.3<br>(-45.5, 53.9)
**DR Congo**	5878<br>(2441, 11907)	20.4<br>(10.4, 33.8)	11876<br>(5757, 19258)	20.7<br>(11.3, 30.8)	1.2<br>(-22.2, 40.4)
**Equatorial Guinea**	79<br>(25, 190)	24.7<br>(10.0, 50.6)	128<br>(69, 221)	15.9<br>(8.8, 27.4)	-35.8<br>(-73.2, 42.7)
**Gabon**	120<br>(49, 211)	15.1<br>(6.1, 25.9)	172<br>(77, 282)	12.7<br>(5.3, 21.3)	-15.6<br>(-48.6, 36.8)
